# Optimization and Testing of an SPE-LC/q-TOF Analytical Method for the Detection of PFAS Degradation Products in Water Treatment Processes

**DOI:** 10.1021/acs.est.5c01886

**Published:** 2025-10-09

**Authors:** Giulia Tomei, Elena Piva, Mubbshir Saleem, Marta Finotto, Michele Pozzebon, Ester Marotta

**Affiliations:** † Department of Chemical Sciences, 9308 University of Padova, Via Marzolo 1, 35131 Padova, Italy; ‡ dtoLABS, Via Pozzuoli, 13C/13D, 30038 Spinea, Veneto, Italy

**Keywords:** liquid chromatography−mass spectrometry, nonthermal plasma, reductive defluorination, H/F exchange products, OH/F exchange products

## Abstract

The research on novel methods for water treatment to achieve the degradation of perfluoroalkyl and polyfluoroalkyl substances (PFAS) is very active and usually considers perfluorooctanoic acid (PFOA) and perfluorooctansulfonic acid (PFOS) to test the process efficacy. Their degradation generally proceeds through the sequential formation of shorter-chain perfluoroalkyl carboxylic acids (PFCAs), which are detected and quantified by means of well-established methods. However, hydro- and hydroxy-defluorinated products of various chain length, in which one or more –F atoms have been substituted by −H atoms and/or –OH groups, can be formed as well. An analytical method was therefore developed, capable of concentrating and simultaneously analyzing PFCAs and these substitution products. Solid-phase extraction and LC-ESI/q-TOF parameters have been optimized to maximize recovery and detection of PFOS, PFOA, shorter-chain PFCAs, and various polyfluoroalkyl and polyfluorohydroxyalkyl carboxylic/sulfonic acids obtained in the treatment of solutions 1.0 × 10^–6^ M PFOA or PFOS in tap water by nonthermal plasma (NTP). The application of the developed analytical method, which was tested on real groundwater samples contaminated with PFAS and treated with NTP, is suggested for studies dealing with advanced reduction/oxidation processes for PFAS removal to verify the absence of polyfluoroalkyl and polyfluorohydroxyalkyl carboxylic/sulfonic acids.

## Graphical abstract

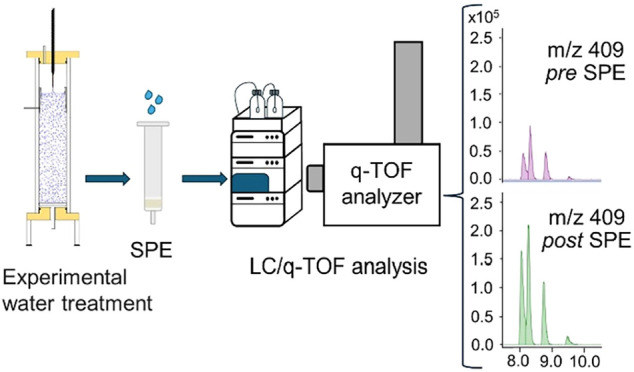

## 1 Introduction

Per- and polyfluoroalkyl substances (PFAS) are synthetic aliphatic organic compounds with peculiar physical-chemical characteristics, such as water and fat repellence, surfactant properties, and chemical and thermal stability, which made them very useful in a wide range of industrial applications and products of mass consumption.^1,2^

As a result of the extensive production and use of PFAS and of their resistance to natural degradation processes, these compounds became ubiquitous^1–6^ and have been classified as persistent organic pollutants (POPs) by Stockholm Convention in 2001.^7,8^ They are, moreover, bioaccumulative and constitute therefore a potential risk for human health, causing a wide range of pathologies;^2–4,6^ the International Agency for Research on Cancer (IARC) has recently classified perfluorooctanoic acid (PFOA) as carcinogenic for humans (Group 1) and perfluorooctanesulfonic acid (PFOS) as potential carcinogenic for humans (Group 2B).^9^

Conventional water treatments have shown no efficacy in the removal of perfluoroalkyl substances from water and also advanced oxidation processes (AOPs) that initiate contaminants degradation through the reaction with OH radicals have proven to be ineffective.^10–12^ The typical methods used for removing PFAS from water include reverse osmosis and sorption on activated carbon or ion exchange resins, followed by incineration of spent sorbent.^11,12^ However, a nondestructive removal is just a temporary solution, producing a waste that must be properly disposed of, otherwise PFAS are again released in the environment, perpetuating the cycle.^13,14^ In the literature, various methods are investigated to remove and degrade PFAS,^6,12,15–20^ among which the most promising, nowadays under evaluation for scaling-up and commercialization, are electrochemical oxidation, sonolysis, and nonthermal plasma treatment.^21^ In electrochemical oxidation, a direct electron transfer from PFAS to the anode surface takes place, forming a fluorinated organic radical which is then oxidized, probably by ^•^OH generated from water oxidation.^12,18,22^ In sonolysis, ultrasonic irradiation induces cavitation and water pyrolysis with the formation of active radicals.^23^ In nonthermal plasma, the application of an electrical discharge in the gas above the water surface generates electrons, excited species, radicals, and photons, whose combined action is capable of degrading PFAS.^15^

In the investigation on new methods for the degradation of PFAS in water, PFOA and PFOS are usually used as test compounds and generally give shorter-chain perfluoroalkyl carboxylic acids as degradation byproducts.^17–31^ Only in some cases, hydro-defluorinated products and, less frequently, hydroxy-defluorinated products have been detected, characterized by substitution of one or more −F atoms with −H atoms and/or −OH groups, respectively, and possibly by elimination of the sulfonic group and chain shortening.^17,24,25,28,32–34^ However, the fate of these products is not clear, and moreover, in most cases, target analyses are performed, not allowing ascertaining their presence (and in general the presence of products different from shorter-chain perfluoroalkyl carboxylic acids). The development of an analytical method that can concentrate and analyze these compounds is thus necessary. Considering conventional and emerging methods for PFAS measurements,^35–37^ the only technique allowing to reach the required sensitivity and selectivity is liquid chromatography coupled with mass spectrometry (LC/MS), preceded by solid-phase extraction (SPE) to concentrate the samples.

In this article, we present a method based on SPE followed by LC-ESI/q-TOF analysis that allows recovery and detection of a wide range of commonly monitored PFAS, as well as polyfluoroalkyl- and polyfluorohydroxyalkyl carboxylic/sulfonic acids. The latter, hereafter indicated as “substitution products”, were produced as “standards” through the treatment of solutions 1.0 × 10^–6^ M of PFOA and PFOS in tap water with nonthermal plasma (NTP) in a previously characterized process.^38^ To evaluate the effectiveness of the newly developed analytical method, it was tested for the analysis of real groundwater samples contaminated with PFAS and treated in a bigger NTP prototype reactor. The results obtained are presented in this paper.

## 2 Materials and Methods

### 2.1 Materials

Native PFAS primary dilution standards (EPA-533 PAR), mass-labeled (^13^C) internal standards (EPA-533 IS), and mass-labeled (^13^C) external standards (EPA-533 ES) were purchased from Wellington Laboratories. The list of PFAS contained in the mixtures with their acronyms is reported in the Supporting Information. API-TOF reference mass solution was purchased from Agilent Technologies. Ultrapure water for mobile phase was obtained by means of a Sartorius Arium Mini apparatus (Sartorius, Goettingen, Germany). Ammonium acetate (NH_4_Ac, reagent grade purity ≥ 99%), perfluorooctanoic acid (PFOA, reagent grade purity ≥ 96%), and heptadecafluorooctanesulfonic acid potassium salt (PFOS, reagent grade purity ≥ 98%) used for SPE recovery tests, ammonium hydroxide solution (28.0–30.0% NH_3_ basis), and methanol (UHPLC-MS grade ≥ 99.99%) were purchased from Sigma-Aldrich. Argon was purchased from Air Liquide with specified impurities of H_2_O (<0.5 ppm), H_2_ (<0.1 ppm), O_2_ (<0.5 ppm), CO_2_ (<0.5 ppm), CO (<0.1 ppm), and total hydrocarbons (THC, <0.1 ppm). The physical properties and chemical composition of the tap water used for the preparation of the solutions of PFAS treated by nonthermal plasma, drawn from the laboratory drinking water faucet, were described in a previous publication.^39^ The samples of groundwater contaminated by PFAS were provided by Acque Veronesi s.c.a r.l. PolyClean 302H (200 mg, 6 mL), and PolyClean 30HAW (150 mg, 6 mL) cartridges for solid-phase extraction were purchased from Interchim.

### 2.2 Experimental Setup for Nonthermal Plasma Treatment

Nonthermal plasma treatments were carried out in two radial plasma (RAP) discharge reactors, characterized by an electrode configuration constituted by a high-voltage pin electrode made in tungsten (Ø 2.5 mm) fixed at around 10 mm above the liquid surface and a stainless-steel ring partially immersed into the liquid as the counter electrode.^8^

The electrical discharge was ignited by a DC high-voltage power supply with negative polarity (Spellmann, PTV30 × 350, 30 kV, 12 mA). The input power was maintained at 4 W by applying a high voltage at −5 kV and regulating the pulse frequency at around 100 Hz by charging a high-voltage capacitor (2.1 nF) connected in parallel to the reactor. Argon was used as the plasma feed gas and was bubbled into the liquid from the bottom of the reactor. Laboratory-prepared solutions of 1.0 × 10^–6^ M PFOA and/or PFOS in tap water (30 mL) were treated in a little prototype equipped with a container made in glass, working with a flow rate of argon of 100 mL/min, as described in a previous work.^8^ PFAS-contaminated groundwater (300 mL) was treated in a novel larger prototype with a container made of plexiglass (300 mm height and internal diameter of 50 mm) (Figure 1), working with an argon flow rate of 300 mL/min.

**Figure 1:**
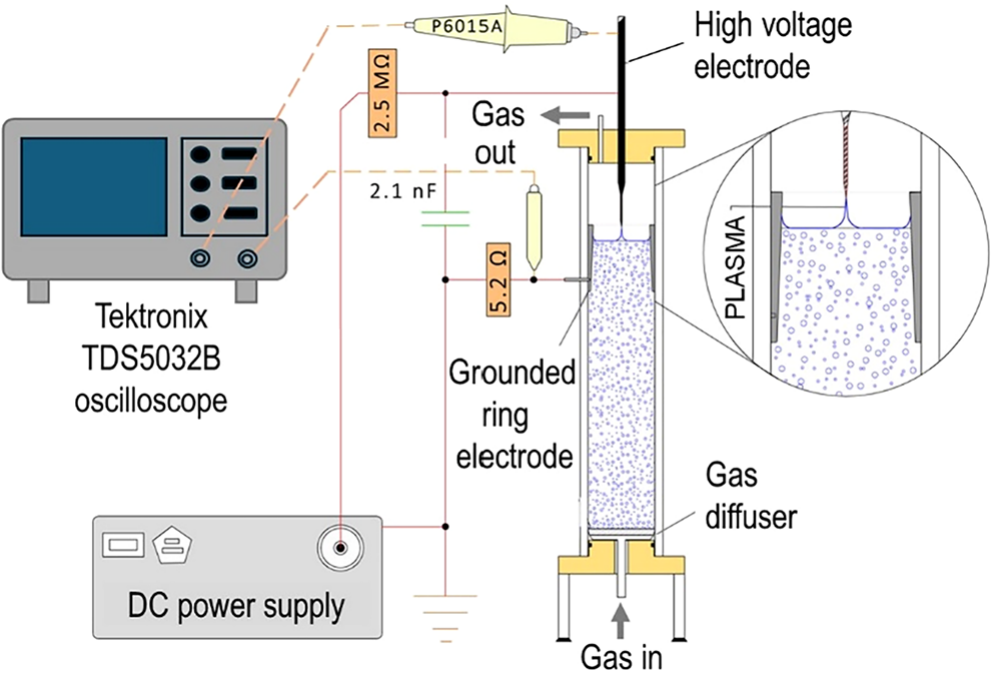
Scheme of the RAP reactor prototype used for the treatment of PFAS-contaminated groundwater. In this system, nonthermal plasma is produced on the water surface by applying a high voltage between the electrodes. Argon is bubbled into the liquid from the bottom of the reactor.

### 2.3 Sample Preparation by Solid-Phase Extraction

SPE was optimized using as a test solution 10 mL of a mixture of two solutions treated with the RAP reactor for 5 min, one initially containing PFOA 1.0 × 10^–6^ M in tap water, the other PFOS 1.0 × 10^–6^ M in tap water.

First, SPE cartridges tested were PolyClean 302H 200 mg/6 mL (Interchim). Conditioning was done with 15 mL of methanol followed by 18 mL of ultrapure water. After sample loading, a washing step with 7.5 mL of ultrapure water was performed, followed by analyte elution in 8 mL of methanol. Samples were dried and recovered with 1 mL of a 1:1 mixture of aqueous NH_4_Ac 5 mM:methanol.

For the second SPE procedure tested, PolyClean 30HAW 150 mg/6 mL cartridges (Interchim) were used as the solid phase. Conditioning consisted of flushing 5 mL of methanol, followed by 5 mL of ultrapure water. 10 mL of sample was loaded, and the cartridges were washed with 3 mL of ultrapure water. Elution was performed with 4 mL of methanol containing 5% NH_4_OH. Samples were dried and recovered with 1 mL of a 1:1 mixture of aqueous NH_4_Ac 5 mM:methanol.

In the third SPE procedure tested, PolyClean 30HAW 150 mg/6 mL cartridges (Interchim) were conditioned with 5 mL of methanol, followed by 5 mL of ultrapure water. 10 mL of sample was loaded, and the cartridges were washed with 3 mL of water. First elution was performed by flushing 4 mL of methanol, and a second elution was carried out with 4 mL of methanol containing 5% NH_4_OH and combined with the first. Samples were dried and recovered with 1 mL of a 1:1 mixture of aqueous NH_4_Ac 5 mM:methanol.

### 2.4 LC/MS Apparatus (LC/q-TOF)

Analyses were carried out with an Agilent 1290 Infinity II series HPLC chromatograph coupled with an Agilent 6546 Q-TOF mass spectrometer equipped with a Dual Jet Stream electrospray source and a q-TOF analyzer. A Poroshell 120 EC-C18 4.6 mm × 50 mm 2.7 μm column (Agilent Technologies) was used as the PFAS delay column, while the chromatographic separation was performed using a Uptisphere strategy C18-HQ 2.1 mm × 50 mm 2.2 μm column (Interchim). The eluents used consist of ammonium acetate (5 mM) in ultrapure water (A) and methanol (B). The gradient for eluent B was as follows: isocratic at 5% for 1 min, from 5 to 85% in 8 min, from 85 to 97% in 1 min, isocratic at 97% for 3 min. The flow rate was set at 0.3 mL/min, and the injection volume was 5 μL. Samples ionization was performed in negative mode (ESI–), with a spray of 2.5 kV and a source temperature of 230 °C. Sheath gas flow and temperature were respectively 12 L/min and 350 °C. The voltage of the fragmentor, i.e., the voltage applied to the exit end of the inlet capillary, was set at 90 V, that of the Skimmer at 65 V, and that of the nozzle at 0 V. The quantification of PFAS was based on calibration curves built using isotopically labeled internal standards and was performed in full scan mode on the accurate mass (<5 ppm) of the [M – H]^−^ ion. The identification of polyfluoroalkyl and polyfluorohydroxyalkyl carboxylic/sulfonic acids was based on high-resolution mass measurements and MS/MS spectra. The experimentally determined masses due to [M – H]^−^ ions were compared with theoretical exact masses calculated from the proposed molecular structures considering a maximum mass deviation of 5 ppm. By using the Tool “Show formula calculator” of the instrument software (Mass Hunter Qualitative Analysis), it was also verified that the assigned elemental compositions were the ones with the *m*/*z* values closest to the experimental *m*/*z* and the unique chemically consistent with the process under consideration. Additionally, diagnostic neutral losses and fragments were identified in the MS/MS spectra.

### 2.5 Data Reproducibility and Uncertainty Calculations

Each sample subjected to SPE was analyzed four times by repeating SPE twice and analyzing twice the solution obtained from each SPE. The results of the four MS analyses were averaged, considering the standard deviation as the uncertainty of the concentration data.

In the case of plasma-treated samples, plasma treatment was repeated twice, and for each treatment, two SPEs were done, and each one was analyzed twice, for a total of eight MS analyses. The results of these MS analyses were averaged by considering the standard deviation as the uncertainty of the concentration data.

The uncertainty associated with the SPE recoveries and with concentrations obtained as a difference of two concentrations was calculated by propagating the error according to the established propagation rules for ratio and difference calculations. The so obtained uncertainties are associated with the recovery data reported in the tables or represented as error bars in the figures reporting concentration data.

## 3 Results and Discussion

### 3.1 Optimization of LC/q-TOF Analysis and Substitution Products Identification

The LC/q-TOF method for the analysis of PFAS in water was optimized both on the mix of standards EPA-533 PAR and on the samples treated by nonthermal plasma, focusing not only on maximizing the signals intensity but also on reducing the poly- and perfluorocarboxylic acids fragmentation into the ionization source. Different source temperatures (150, 190, and 230 °C), sheath gas temperatures (200, 250, 300, and 350 °C), and voltages of the fragmentor (60, 90, and 115 V), skimmer (20, 40, and 65 V), and octapole (200, 400, and 750 V) were tested.

The source and the sheath gas temperature turned out to not influence the fragmentation, since the proportion between the signals of the parent ion [M – H]^−^ and of the fragmentation product [M – H – CO_2_]^−^ remained unchanged despite the temperature increase (Figures S1 and S2). No influence on the fragmentation was observed also by changing the Skimmer and the octapole voltages (Figures S3 and S4); thus, the final values of these parameters were set considering the highest intensities of the signals related to the parent ion [M – H]^−^: source temperature of 230 °C, sheath gas temperature of 350 °C, skimmer voltage of 65 V, and octapole voltage of 750 V.

As expected, in-source fragmentation of the carboxylic acids was instead influenced by the voltage of the fragmentor: with 60 V, the signal of short-chain PFAS (*C* < 5) was almost lost, so 60 V was discarded; comparing 90 and 115 V (Figure S5), [M – H – CO_2_]^−^ ions were detected with higher intensities at 115 V than at 90 V, while [M – H]^−^ ions were higher at 90 V than at 115 V (Figures S5 and S6). The voltage of the fragmentor at 90 V thus appeared to be the best condition and was set in the method used for the analyses. A list of all the compounds monitored in this work with their retention times and exact masses of their corresponding ions is reported in Table S1. MS and MS/MS spectra of the identified polyfluoroalkyl and polyfluorohydroxyalkyl carboxylic/sulfonic acids are reported in Figures S7–S22. Hydroxyl functionality in carboxylic acids was confirmed by the loss of CF_2_O,^40^ always following the losses of H_2_O and CO_2_. The hydro-substitution was instead characterized by HF loss, as reported in previous publications.^41,42^ The fragmentation schemes of some substitution products are reported as examples in Figures S23–S25.

The limit of detection (LOD) and the limit of quantification (LOQ) of the PFAS contained in the mix of standard EPA-533 PAR were calculated by the signal-to-noise ratio (S/N) of the deprotonated ions. LOD and LOQ were defined as S/N equal to 3 and 10, respectively. LOD and LOQ determined for PFAS contained in the mix of standard EPA-533 PAR are reported in Table 1.

**Table 1:** LOD and LOQ Calculated for PFAS Contained in the Mix of Standards EPA-533 PAR

compound	LOD (ppb)	LOQ (ppb)
PFBA	0.264	0.881
PF4OPeA	0.202	0.675
PFPeA	0.140	0.468
PF5OHxA	0.170	0.569
HFPO-DA	0.213	0.710
L-PFBS	0.070	0.235
PFHxA	0.105	0.349
PFEESA	0.088	0.294
4:2FTS	0.327	1.091
L-PFPeS	0.130	0.435
PFHpA	0.100	0.333
NaDONA	0.293	0.977
PFHxSK	0.191	0.638
PFOA	0.128	0.428
6:2FTS	0.488	1.628
L-PFHpS	0.096	0.323
PFNA	0.755	2.519
PFOSK	0.083	0.277
PFDA	0.125	0.419
8:2FTS	0.225	0.751
9Cl-PF3ONS	0.056	0.188
PFUdA	0.078	0.261
PFDoA	0.110	0.366
11Cl-PF3OUdS	0.047	0.158

### 3.2 SPE Procedure Optimization

From the literature, two main classes of products are known to be formed from PFOA and PFOS degradation induced by reductive/oxidative advanced processes (Scheme 1):^17–34^ perfluorinated carboxylic acids with a shorter alkyl chain with respect to the original compound (*C*_*n*–1_ formed from *C_n_*, *C*_*n*–2_ from *C*_*n*–1_, etc.) and polyfluorinated carboxylic/sulfonic acids of variable chain length in which one or more −F atoms are substituted by −H atoms or −OH groups, here referred to as substitution products. While PFCAs with a shorter carbon chain are sequentially formed from the higher homologue through a process initiated by an oxidation or reduction step,^25^ perfluorosulfonic acids (PFSAs) with a number of carbon atoms lower than 8 are present as impurities in the commercial PFOS sample used to prepare the solutions subjected to NTP treatment. Consequently, polyfluoro- and polyfluorohydroxy-sulfonic acids detected in the NTP-treated solutions are supposed to derive from their perfluorinated sulfonic acids analogues, since there is no evidence in the literature that sulfonic acids follow a chain shortening process analogous to that of carboxylic acids (Scheme 1).

**Scheme 1:**
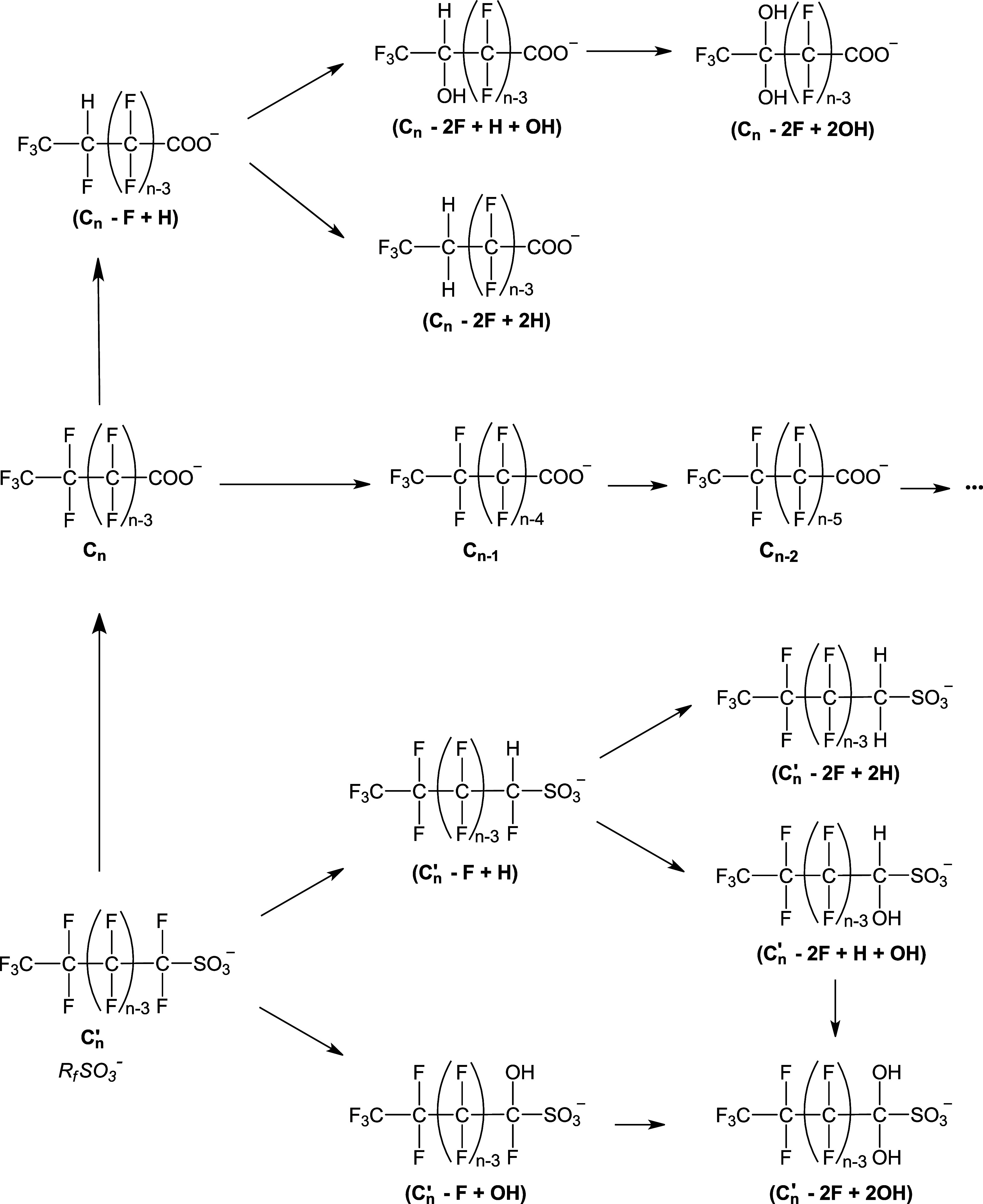
Degradation Pathway of PFCAs and PFSAs Treated by Nonthermal Plasma in Contact with the Aqueous Solution^a^ ^25^

a The formation of more than one isomer is observed in defluorination and hydro and hydroxy substitution, indicating that these reactions are not limited to a single site of the alkyl chain, as shown, for simplicity, in the scheme. For details on the reaction mechanisms, see ref 25.

Analytical methods for PFCAs are well established^43–45^ and always applied to detect and quantify these compounds in the treated water; on the contrary, the substitution products are often ignored. It is therefore important to develop a method for the preconcentration and analysis of the samples that allows detection of both these classes of degradation products. To this aim, the solid-phase extraction (SPE) procedure was optimized on laboratory-prepared solutions of PFOA and PFOS 1.0 × 10^–6^ M in tap water separately treated by nonthermal plasma in the RAP reactor for 5 min and then mixed. It was indeed observed that under these conditions, PFCAs of shorter alkyl chain and various substitution products are formed at a directly detectable concentration. 1 mL of the obtained plasma-treated solution was addressed to LC/MS analysis without any sample pretreatment to be used as the reference, while the rest of the solution was employed in the SPE optimization tests, as described below.

The first SPE procedure tested was performed using PolyClean 302H cartridges (Interchim) as the solid phase and 8 mL of methanol as the eluent. This solid phase is a modified polymer designed to retain a broad range of compounds from various matrices through hydrophilic and hydrophobic interactions. Using this procedure, as shown in the example in Figure S26, polyfluorinated acids as well as perfluorinated carboxylic and sulfonic acids with alkyl chains from 5 carbon atoms upward were efficiently concentrated and recovered. On the contrary, very low recoveries were obtained for PFBA (Figure S26). Since the interaction of this solid phase with the compounds is based only on hydrophobic and hydrophilic interactions, it is possible that PFBA was completely not retained or washed out with water prior to the elution. Thus, a different solid phase was tested.

The second SPE procedure tested was performed using PolyClean HAW cartridges (Intechim) as the solid phase and 4 mL of methanol containing NH_4_OH 5% as the eluent, according to the standard procedures used for WAX-type cartridges, commonly used for PFAS.^43^ The stationary phase of PolyClean HAW cartridges contains a modified polymer that allows both hydrophobic and weak anionic exchange interactions to enhance the cleanup of acidic compounds (p*K*_a_ < 5). This procedure allowed us to recover PFBA as well as the longer-chain per- and poly-fluorocarboxylic/sulfonic acids, but polyfluorohydroxy carboxylic/sulfonic acids were no more detectable after the purification (Figure S27).

For this reason, still using PolyClean HAW cartridges (Intechim), the elution solvent was modified and two subsequent elutions were performed and combined: the first with 4 mL of methanol and the second with 4 mL of methanol containing NH_4_OH 5%. Under these conditions, all of the investigated PFAS were effectively recovered (Figure 2). Moreover, recovery percentages were comparable in the case of isomers, allowing their relative ratio to be respected: an example is shown in Figure 2a,b for the ion at *m*/*z* 409 corresponding to a polyfluorodihydroxy carboxylic acid. This SPE procedure was, therefore, adopted for the concentration of all the following samples analyzed in this work.

**Figure 2:**
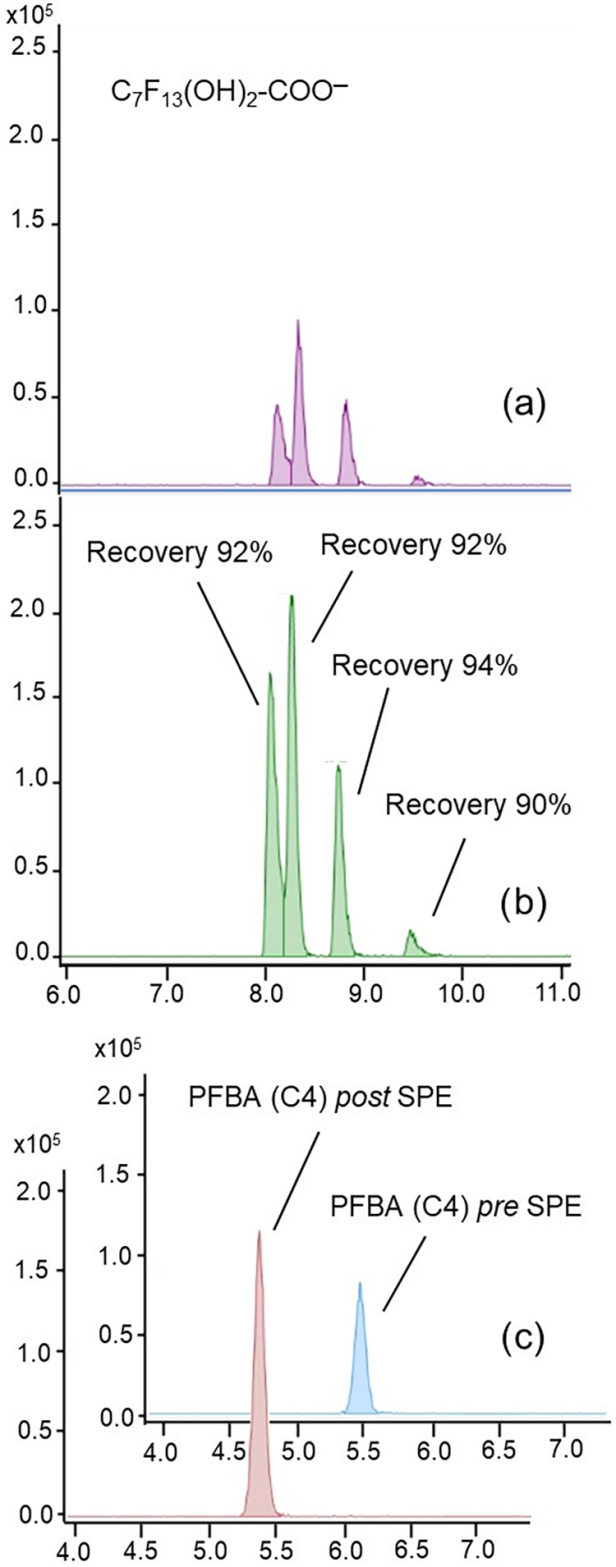
(a) EIC of the polyfluorodihydroxy carboxylic acid (PFOA – 2F + 2OH) (*m*/*z* 408.9751) in the original sample and (b) after SPE optimized procedure. (c) Comparison between EIC of PFBA (C4) before and after the SPE.

After the extraction procedure was optimized by concentrating the samples 10 times, a scale-up was performed by loading 100 mL of solution and concentrating 100 times. To stabilize the pH and increase the reproducibility of the extraction, prior to the loading into the SPE cartridge, ammonium acetate was added to the samples to reach 5 mM.

Recoveries of perfluorinated carboxylic and sulfonic acids in the scaled optimized SPE procedure were evaluated by spiking 50 ppt of the mix of standards EPA-533 PAR (native compounds) and 10 ppt of the mix of mass-labeled (^13^C) external standards EPA-533 ES in 100 mL of ultrapure water.

The use of these standard mixtures allowed testing of the optimized SPE procedure for other PFAS of environmental interest. Table 2 reports the recoveries for each compound contained in the mix of standards.

**Table 2:** Recoveries after SPE of PFAS Contained in the Mix of Standard EPA-533 PAR

compound	recoveries	compound	recoveries
PFBA	(92 ± 8)%	NaDONA	(92 ± 7)%
PF4OPeA	(89 ± 7)%	6:2FTS	(85 ± 15)%
PFPeA	(93 ± 10)%	L-PFHpS	(87 ± 4)%
L-PFBS	(97 ± 7)%	PFOA	(97 ± 6)%
PF5OHxA	(94 ± 9)%	PFNA	(85 ± 7)%
PFEESA	(98 ± 7)%	PFOSK	(69 ± 3)%
4:2FTS	(99 ± 12)%	9Cl-PF3ONS	(51 ± 2)%
PFHxA	(107 ± 10)%	PFDA	(67 ± 4)%
L-PFPeS	(94 ± 5)%	8:2FTS	(62 ± 5)%
HFPO-DA	(93 ± 8)%	PFUdA	(47 ± 3)%
PFHxSK	(93 ± 5)%	11Cl-PF3OUdS	(29 ± 4)%
PFHpA	(94 ± 7)%	PFDoA	(38 ± 2)%

Recoveries are good for most of the compounds, while they are not completely satisfactory for compounds with a number of carbon atoms higher than eight, which were not present in the solution used for SPE optimization.

Due to the lack of standard compounds, recoveries of the substitution products were determined differently with respect to the PFAS included in the EPA-533 PAR mixture: the sample obtained from mixing the solutions 1.0 × 10^–6^ M of PFOA and PFOS separately treated for 5 min within the RAP reactor was directly analyzed by LC/ESI-q-TOF. The same sample was then subjected to SPE, and recoveries were calculated with respect to the results of the direct analysis considering the dilution factor. The results are reported in Table 3; in the case of different isomers of the same molecule, the recoveries correspond to the average values found for the different isomers since, as shown in the example of Figure 2b concerning *m*/*z* 409, they were always comparable.

**Table 3:** Recoveries after SPE of the Substitution Products Formed during Plasma Treatment

compound		structure^a^	recovery
*m*/*z* 409	(PFOA – 2F + 2OH)	C_7_F_13_(OH)_2_-COO^–^	(92 ± 1)%
*m*/*z* 359	(PFHpA – 2F + 2OH)	C_6_F_11_(OH)_2_-COO^–^	(89 ± 1)%
*m*/*z* 309	(PFHxA – 2F + 2OH)	C_5_F_9_(OH)_2_-COO^–^	(89 ± 2)%
*m*/*z* 393	(PFOA – 2F + OH + H)	C_7_F_13_H(OH)-COO^–^	(81 ± 1)%
*m*/*z* 343	(PFHpA – 2F + OH + H)	C_6_F_11_H(OH)-COO^–^	(89.7 ± 0.5)%
*m*/*z* 293	(PFHxA – 2F + OH + H)	C_5_F_9_H(OH)-COO^–^	(95 ± 3)%
*m*/*z* 243	(PFPeA – 2F + OH + H)	C_4_F_7_H(OH)-COO^–^	(88 ± 6)%
*m*/*z* 395	(PFOA – F + H)	C_7_F_14_H-COO^–^	(84.4 ± 0.1)%
*m*/*z* 345	(PFHpA – F + H)	C_6_F_12_H-COO^–^	(103 ± 2)%
*m*/*z* 295	(PFHxA – F + H)	C_5_F_10_H-COO^–^	(83 ± 2)%
*m*/*z* 245	(PFPeA – F + H)	C_4_F_8_H-COO^–^	(90 ± 1)%
*m*/*z* 495	(PFOS – 2F + 2OH)	C_8_F_15_(OH)_2_-SO_3_^–^	(112 ± 2)%
*m*/*z* 497	(PFOS – F + OH)	C_8_F_16_(OH)-SO_3_^–^	(109 ± 2)%
*m*/*z* 381	(PFHxS – F + H)	C_6_F_12_H-SO_3_^–^	(90 ± 1)%
*m*/*z* 281	(PFBS – F + H)	C_4_F_8_H-SO_3_^–^	(73 ± 1)%
*m*/*z* 231	(PFPrS – F + H)	C_3_F_6_H-SO_3_^–^	(86.51 ± 0.04)%

a Generic chemical structures are reported because various isomers are observed.

The table shows that the recoveries for the substitution products are good, in most cases, higher than 81%. (PFHpA – F + H), (PFOS – 2F + 2OH), and (PFOS – F + OH) are slightly overestimated; however, it was verified that they are not detected in blank samples obtained from SPE of tap water; thus, there is no systematic error.

### 3.3 Application of the Method on a Real Water Sample Treated with Nonthermal Plasma

To verify the effectiveness of the analytical method described above, it was tested on a real PFAS-contaminated groundwater sample treated in the bigger RAP reactor prototype (Figure 1). The groundwater sample, independently analyzed by Acqueveronesi S.c.a.r.l. following the ISTISAN 2019/07 method,^46^ contained PFCAs and PFSAs with a number of carbon atoms from 8 down, as reported in correspondence with the treatment time “0 min” in Figures 3 and 4. Treatments of 15, 30, and 60 min in portions of 300 mL were performed in duplicate; treated and nontreated samples were concentrated by solid-phase extraction and analyzed by LC/q-TOF following the new optimized methodology. The analytical method allowed the simultaneous recovery and detection of PFCAs, PFSAs, and hydro- and hydroxy-defluorinated substitution products. Due to the lack of standard compounds, the concentration of the substitution products was estimated by considering that their instrumental response was comparable to that of their perfluorinated analogues. This choice was based on the similarity of *m*/*z* ratios between substitution products and perfluorinated analogues due to the observation that the signal response and the signal-to-noise ratio were more influenced by *m*/*z* ratios than by the solvent composition. The recoveries of the PFAS listed in Table 2 were evaluated based on the mix of mass-labeled (^13^C) external standards EPA-533 ES. The recoveries obtained in the groundwater for these standards were consistent with those obtained in ultrapure water, and for this reason, we assumed that the matrix effects were negligible also in the case of the substitution products, and no corrections were made. The results are presented in Figures 3 and 4, referring to fluorinated sulfonic and carboxylic acids, respectively. In these figures, the estimated concentrations of the substitution products are summed grouping them based on the substitution type (hydro- or hydroxy-) and the number of carbon atoms.

**Figure 3:**
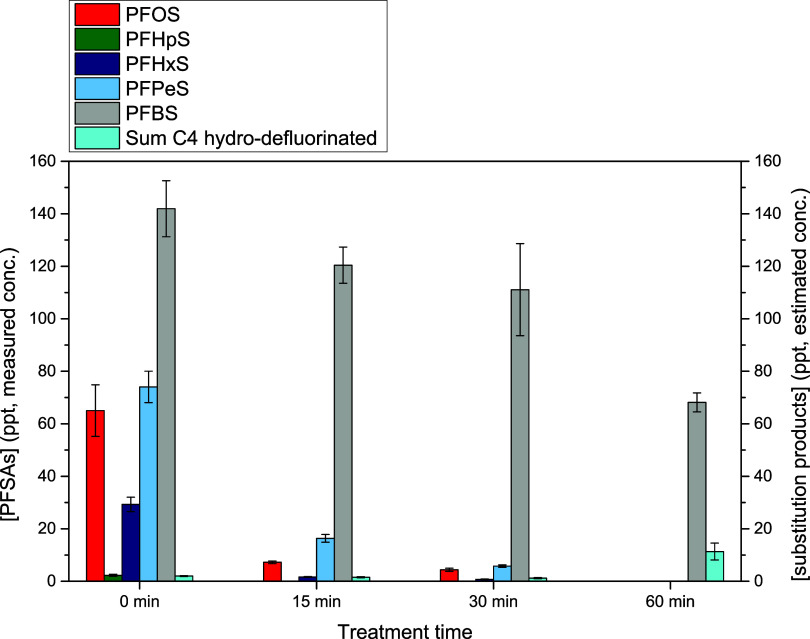
Concentration of the fluorosulfonic acids (perfluorinated on the left *y*-axis, polyfluorinated on the right *y*-axis) detected in the untreated groundwater (0 min) and after 15, 30, and 60 min of nonthermal plasma treatment.

**Figure 4:**
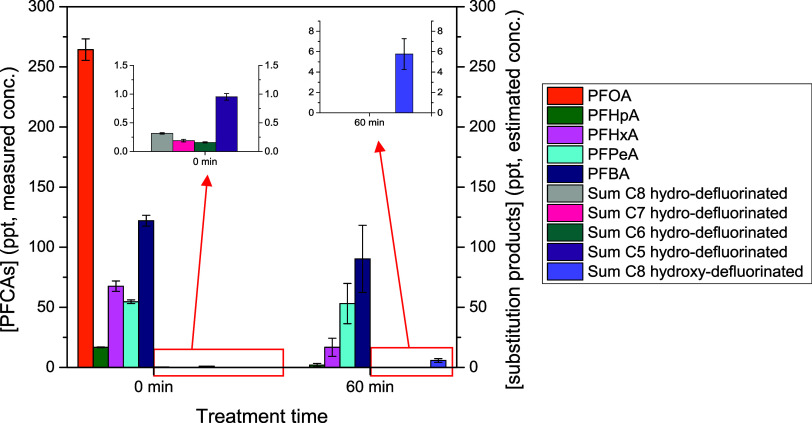
Concentration of the fluorocarboxylic acids (perfluorinated on the left *y*-axis, polyfluorinated and polyhydroxyfluorinated on the right *y*-axis) detected in the untreated groundwater (0 min) and after 60 min of nonthermal plasma treatment (perfluorinated carboxylic acids, hydro-defluorinated and hydroxy-defluorinated substitution products are distinguished based on the number of carbon atoms).

Besides PFCAs and PFSAs with a carbon chain from 8 to 4 atoms, the untreated groundwater sample contained some polyfluorinated carboxylic and sulfonic acids with the same retention time and *m*/*z* as the hydro-defluorinated products reported in Table 3. The initial concentrations of PFCAs and PFSAs and the estimated initial concentrations of the hydro-defluorination products are reported in correspondence with the treatment time “0 min” in Figures 3 and 4. A detailed list of the monitored and detected PFAS with their exact masses, attributions, and retention times is given in Table S1.

As shown in Figure 3, the most abundant PFSA in the groundwater sample was PFBS. Perfluorosulfonic acids were efficiently degraded during the treatment: after 30 min of plasma application, 93% of the initially present PFOS (perfluorooctanesulfonate) was degraded, PFHpS (perfluoroheptanesulfonate) was completely removed, 97% of PFHxS (perfluorohexanesulfonate) and 92% of PFPeS (perfluoropentanesulfonate) were, moreover, decomposed. After 60 min of treatment, all of them were under the limit of detection (PFOS < 0.8 ppt, PFHpS < 1.0 ppt, PFHxS < 1.9 ppt, PFPeS < 1.3 ppt). On the contrary, PFBS (perfluorobutanesulfonate) was degraded slowly during the treatment, reaching 52% of removal after 60 min. This is consistent with previous findings^34,47–49^ and was attributed to the fact that PFBS has a less surfactant character than longer-chain PFAS (CMC in water at 32 °C of 22 mM, with respect to PFOS, which has a CMC of 7 mM),^50^ so with more difficulty it reaches the surface of the liquid to be in direct contact with plasma. Moreover, from previous studies, it is known that electrons are the main species responsible for initiating PFAS degradation^24,25,51^ and, for perfluorosulfonic acids, a shorter perfluoroalkyl chain corresponds to a reduced reactivity with electrons (1.90 × 10^5^ M^–1^s^–1^ for PFBS, with respect to 1.95 × 10^6^ M^–1^s^–1^ for PFOS).^52^ The only detected substitution product with a sulfonate group was octafluorobutanesulfonic acid (i.e., the monohydrodefluorinated sulfonic acid with 4 carbon atoms). It was already present in the groundwater, and its concentration increased during the treatment, most probably due to its formation from the conversion of PFBS.

To interpret the results regarding PFCAs and substitution products, it must be taken into account the degradation pathway known from the literature^24,25,38^ and represented in Scheme 1. It is known that in the plasma treatment, PFSAs are converted into PFCAs and that the degradation of long-chain PFCAs gives rise to the formation of shorter-chain PFCAs through subsequent steps in which a formal loss of −CF_2_– takes place.^24,25,38,47^ Short-chain PFCAs are thus formed during the treatment from the homologues PFSAs and from the degradation of the longer-chain PFCAs but are also present in the original sample. Thus, while PFOA continuously decreased during the treatment and was efficiently removed (>99% of degradation in 60 min), PFHpA, PFHxA, PFPeA, and PFBA showed the typical trend of intermediate products, which initially increase, reaching a maximum, and then decrease (Figure S28). After 60 min, the concentrations of PFHpA and PFHxA were, respectively, 80 and 67% less than the initial one, while PFPeA and PFBA concentrations were comparable to those in the original samples.

In Scheme 1, it is shown that the substitution products are formed from the homologues PFCAs and PFSAs. One possible defluorinated isomer is drawn in the scheme, but, as mentioned in Section 3.2 and reported in Table S1, for most substitution products, more than one isomer was detected, indicating that defluorination does not occur in a preferential position of the alkyl chain, as also evidenced in a previous work in a similar plasma reactor.^25^ Polyfluorinated carboxylic acids with alkyl chain from 5 to 8 carbon atoms were detected in the untreated groundwater sample, and their amount increased during the treatment (Figure S29). Hydroxy-defluorinated products, specifically carboxylic acids with an alkyl chain from 6 to 8 carbon atoms, were instead detected only after the plasma process (Figure S30). After 60 min of plasma treatment, hydroxy-defluorinated products containing 6 and 7 carbon atoms were no longer detectable, suggesting that not formed in a significant amount and, however, decomposed, while the homologue containing 8 carbon atoms was accumulated.

From blank experiments aimed at verifying a possible contribution of adsorption or release of PFAS from the experimental apparatus, it was found that when noncontaminated tap water is treated in the RAP reactor, the release of some parts per million of the measured carboxylic acids occurs. In particular, after 60 min, the concentration of the various analytes ranged between 1.5 and 34.0 ppt, as detailed in Table S2. Subtracting these contributions from the concentration values measured (or estimated, in the case of the substitution products) in the 60 min-treated groundwater, the graph reported in Figure 4 is obtained, from which it is evident that after 60 min of treatment, most of the PFAS initially present in the groundwater have been degraded as also most of the substitution products, which are mainly formed during the treatment and are, after 60 min of treatment, all estimated to be lower than 10 ppt.

In summary, the presented SPE-LC-ESI/q-TOF method, optimized on solutions of PFOA and PFOS treated by nonthermal plasma, was employed to quantify the concentration of PFCAs and PFSAs in a groundwater sample before (few hundreds of ppt) and after its treatment by nonthermal plasma for 15, 30, and 60 min. Substitution products, present with multiple isomers, were detected as well, and their concentration was estimated considering an instrumental response similar to that of their perfluorinated homologues. The application of this method allowed us to confirm the efficacy of the employed radial plasma reactor in degrading long-chain PFCAs and PFSAs, while short-chain homologue compounds were decomposed significantly more slowly. Substitution products of different chain lengths were produced in traces, but in most cases also degraded prolonging the treatment time. The application of this method is thus suggested in all the studies dealing with the degradation of PFAS and in particular of PFCAs and PFSAs with a carbon chain length from 4 to 8, as a tool both for investigating the mechanism of the reactions involved in the process through the study of the products and for verifying the absence of substitution products at the end of the treatment. The formation of new PFAS through methods intended for their elimination must indeed be strictly avoided, especially considering that the toxicological properties of these products are unknown. In the development of new technologies for PFAS degradation, it is essential to determine whether any substitution products are generated, and if they are formed as intermediates, the ability of the system to degrade them must be ensured by applying adequate treatment times and carefully monitoring the outcomes.

## Supplementary Material



## Notes

Compositions of the PFAS standard mixtures, additional tables and figures (PDF)
