# Supplementary material for: Optimization and Testing of an SPE-LC/q-TOF Analytical Method for the Detection of PFAS Degradation Products in Water Treatment Processes
Source: Environ Sci Technol. 2025 Oct 9;59(41):22251–61. doi: 10.1021/acs.est.5c01886 (PMC12550814; doi:10.1021/acs.est.5c01886)

Supporting info

of

Optimization and testing of an SPE-LC/q-TOF analytical method for the detection of PFAS degradation products in water treatment processes

Giulia Tomei[1], Elena Piva[2], Mubbshir Saleem[1], Marta Finotto[2], Michele Pozzebon[2], Ester Marotta[1*]

[1]*Department of Chemical Sciences, University of Padova, Via Marzolo 1, 35131, Padova, Italy*

[2]*dtoLABS, Via Pozzuoli, 13C/13D, 30038 Spinea (VE), Italy*

ester.marotta@unipd.it

This file consists of 51 pages containing text, 2 Tables and 30 Figures.

Text: Details on the compositions of the PFAS standard mixtures

Tables: Table S1**.** Retention times, signals in the mass spectrum (m/z values) and identity assignment for all the PFAS monitored in the present study.

Table S2. Release of PFAS during the treatment of uncontaminated tap water for 60 min.

Figures: Figures S1-S6. Effect of the temperature of the source and of sheath gas and effect of the voltages of Skimmer, Octapole and Fragmentor voltages on peaks area of selected ions.

Figures S7-S22. EIC, MS and MS/MS spectra of the substitution products.

Figures S23-S25. Hypothetical structures and fragmentation mechanisms of some substitution products.

Figures S26-S27. Examples of EIC before and after SPE using the first two tested procedures described in the text.

Figures S28-S30. Concentration of PFCAs and substitution products in the untreated groundwater (0 min) and after 15, 30 and 60 minutes of non-thermal plasma treatment.

Text

Details on the compositions of the PFAS standard mixtures:

Native PFAS primary dilution standards (EPA-533 PAR), > 98% purity, were purchased from Wellington Laboratories: Perfluoro-n-butanoic acid (PFBA); Perfluoro-n-pentanoic acid (PFPeA); Perfluoro-n-hexanoic acid (PFHxA); Perfluoro-n-heptanoic acid (PFHpA); Perfluoro-n-octanoic acid (PFOA); Perfluoro-n-nonanoic acid (PFNA); Perfluoro-n-decanoic acid (PFDA); Perfluoroundecanoic acid (PFUnA); Perfluoro-n-dodecanoic acid (PFDoA); 2,3,3,3-Tetrafluoro-2-(1,1,2,2,3,3,3-heptafluoropropoxy)-propanoic acid (HFPO-DA); Perfluoro-4-oxapentanoic acid (PF4OPeA); Perfluoro-5-oxahexanoic acid (PF5OHxA); Perfluoro-3,6-dioxaheptanoic acid (3,6-OPFHpA); Potassium perfluoro-1-butanesulfonate (L-PFBS); Sodium perfluoro-1-pentanesulfonate (L-PFPeS); Potassium perfluorohexanesulfonate (PFHxSK); Sodium perfluoro-1-heptanesulfonate (L-PFHpS); Potassium perfluorooctanesulfonate (PFOSK); Sodium 1H,1H,2H,2H-perfluoro-1-hexanesulfonate (4:2FTS); Sodium 1H,1H,2H,2H-perfluoro-1-octanesulfonate (6:2FTS); Sodium 1H,1H,2H,2H-perfluoro-1-decanesulfonate (8:2FTS); Sodium dodecafluoro-3H-4,8-dioxanonanoate (NaDONA); Potassium 9-chlorohexadecafluoro-3-oxanonane-1-sulfonate (9Cl-PF3ONS); Potassium 11-chloroeicosafluoro-3-oxaundecane-1-sulfonate (11Cl-PF3OUdS); Potassium perfluoro(2-ethoxyethane)sulfonate (PFEESA).

Mass-labelled (^{13}C) internal standards (EPA-533 IS), ⩾99% purity, used as internal standard in the LC/MS analyses of the samples were also from Wellington Laboratories: Perfluoro-n-[1,2-$^{13}C_2$]octanoic acid (M2PFOA); Sodium perfluoro-1-[1,2,3,4-$^{13}C_4$]octanesulfonate (MPFOS).

Mass-labelled (^{13}C) external standards (EPA-533 ES), chemical purity > 98%, isotopic purity ⩾99% were purchased from Wellington Laboratories and were used to determine the recovery percentages obtained through the pre-concentration of the samples by solid-phase extraction: Perfluoro-n-[$^{13}C_4$]butanoic acid (MPFBA); Perfluoro-n-[$^{13}C_5$]pentanoic acid (M5PFPeA); Perfluoro-n-[1,2,3,4,6-$^{13}C_5$]hexanoic acid (M5PFHxA); Perfluoro-n-[1,2,3,4-$^{13}C_4$]heptanoic acid (M4PFHpA); Perfluoro-n-[$^{13}C_8$]octanoic acid (M8PFOA); Perfluoro-n-[$^{13}C_9$]nonanoic acid (M9PFNA); Perfluoro-n-[1,2,3,4,5,6-$^{13}C_6$]decanoic acid (M6PFDA); Perfluoro-n-[1,2,3,4,5,6,7-$^{13}C_7$]undecanoic acid (M7PFUdA); Perfluoro-n-[1,2-$^{13}C_2$]dodecanoic acid (MPFDoA); 2,3,3,3-Tetrafluoro-2-(1,1,2,2,3,3,3-heptafluoropropoxy)-$^{13}C_3$-propanoic acid (M3HFPO-DA); Sodium perfluoro-1-[2,3,4-$^{13}C_3$]butanesulfonate (M3PFBS); Sodium perfluoro-1-[1,2,3-$^{13}C_3$]hexanesulfonate (M3PFHxS); Sodium perfluoro-1-[$^{13}C_8$]octanesulfonate (M8PFOS); Sodium 1H,1H,2H,2H-perfluoro-1-[1,2-$^{13}C_2$]hexanesulfonate (M2-4:2FTS); Sodium

1H,1H,2H,2H-perfluoro-1-[1,2-$^{13}C_2$]octanesulfonate (M2-6:2FTS); Sodium 1H,1H,2H,2H-perfluoro-1-[1,2-$^{13}C_2$]decanesulfonate (M2-8:2FTS).

Tables

Table S1. Retention times, signals in the mass spectrum (m/z values) and identity assignment for all the PFAS monitored in the present study.

EPA-533 PAR mix	Neutral (M)	Ion	m/z values	RT (min)
PFBA	$C_4F_7O_2H$	$[M-H]^-$<br>$[M-H-CO_2]^-$	212.9792<br>168.9894	5.37
PF4OPeA	$C_4F_7O_3H$	$[M-H]^-$	228.9741	6.96
PFPeA	$C_5F_9O_2H$	$[M-H]^-$<br>$[M-H-CO_2]^-$	262.9765<br>218.9862	8.30
PF5OHxA	$C_5F_9O_3H$	$[M-H]^-$	278.9709	8.67
HFPO-DA	$C_6F_{11}O_3H$	$[M-H-CO_2]^-$	284.9778	9.25
L-PFBS	$C_4F_9SO_3K$	$[M-H]^-$	298.9430	8.62
PFHxA	$C_6F_{11}O_2H$	$[M-H]^-$<br>$[M-H-CO_2]^-$	312.9744<br>268.9830	9.33
PFEESA	$C_4F_9SO_4K$	$[M-H]^-$	314.9379	8.99
4:2FTS	$C_6H_4F_9SO_3Na$	$[M-H]^-$	326.9743	9.26
L-PFPeS	$C_5F_{11}SO_3Na$	$[M-H]^-$	348.9398	9.42
PFHpA	$C_7F_{13}O_2H$	$[M-H]^-$<br>$[M-H-CO_2]^-$	362.9707<br>318.9798	9.70
NaDONA	$C_7HF_{12}O_4Na$	$[M-H]^-$	376.9689	10.00
PFHxSK	$C_6F_{13}SO_3K$	$[M-H]^-$	398.9366	9.99

PFOA	$C_8F_{15}O_2H$	$[M-H]^-$<br>$[M-H-CO_2]^-$	412.9672<br>368.9766	10.44
6:2FTS	$C_8H_4F_{13}SO_3Na$	$[M-H]^-$	426.9679	10.42
L-PFHpS	$C_7F_{15}SO_3Na$	$[M-H]^-$	448.9334	10.43
PFNA	$C_9F_{17}O_2H$	$[M-H]^-$<br>$[M-H-CO_2]^-$	462.9632<br>418.9734	10.83
PFOSK	$C_8F_{17}SO_3K$	$[M-H]^-$	498.9302	10.82
PFDA	$C_{10}F_{19}O_2H$	$[M-H]^-$<br>$[M-H-CO_2]^-$	512.9600<br>468.9702	11.17
8:2FTS	$C_{10}H_4F_{17}SO_3Na$	$[M-H]^-$	526.9625	11.18
9Cl-PF3ONS	$C_8ClF_{16}O_4SH$	$[M-H]^-$	530.8956	10.99
PFUdA	$C_{11}F_{21}O_2H$	$[M-H]^-$<br>$[M-H-CO_2]^-$	562.9568<br>518.9670	11.44
PFDoA	$C_{12}F_{23}O_2H$	$[M-H]^-$<br>$[M-H-CO_2]^-$	612.9532<br>568.9638	11.68
11Cl-PF3OUdS	$C_{10}ClF_{20}O_4SH$	$[M-H]^-$	630.8892	11.53
**Poly-fluorinated byproducts**	**Neutral (M)**		**m/z values**	**RT (min)**
m/z 409 (PFOA − 2F + 2OH)	$C_8F_{13}O_4H_3$	$[M-H]^-$<br>$[M-H-H_2O]^-$	408.9751<br>390.9645	8.0, 8.33, 8.8, 9.5

m/z 359 (PFHpA − 2F + 2OH)	$C_7F_{11}O_4H_3$	$[M-H]^-$<br>$[M-H-H_2O]^-$	358.9783<br>340.9677	7.0, 7.5, 8.9
m/z 309 (PFHxA − 2F + 2OH)	$C_6F_9O_4H_3$	$[M-H]^-$<br>$[M-H-H_2O]^-$	308.9815<br>290.9709	3.7, 7.3
m/z 393 (PFOA − 2F + OH + H)	$C_8F_{13}O_3H_3$	$[M-H]^-$	392.9802	8.8, 9.1, 9.2, 9.5, 9.9
m/z 343 (PFHpA − 2F + OH + H)	$C_7F_{11}O_3H_3$	$[M-H]^-$	342.9834	8.1, 8.2, 8.8, 9.3
m/z 293 (PFHxA − 2F + OH + H)	$C_6F_9O_3H_3$	$[M-H]^-$	292.9866	7.2, 8.2
m/z 243 (PFPeA − 2F + OH + H)	$C_5F_7O_3H_3$	$[M-H]^-$	242.9898	2.7, 4.8
m/z 395 (PFOA − F + H)	$C_8F_{14}O_2H_2$	$[M-H]^-$	394.9758	9.3, 9.5, 9.6, 9.8, 10.1, 10.15, 10.2
m/z 345 (PFHpA − F + H)	$C_7F_{12}O_2H_2$	$[M-H]^-$	344.9790	8.9, 9.1, 9.4, 9.6, 9.7
m/z 295 (PFHxA − F + H)	$C_6F_{10}O_2H_2$	$[M-H]^-$	294.9822	8.1, 8.5, 8.7, 8.9
m/z 245 (PFPeA − F + H)	$C_5F_8O_2H_2$	$[M-H]^-$	244.9854	6.3, 6.9, 7.1, 7.3
m/z 495 (PFOS − 2F + 2OH)	$C_8F_{15}SO_5H_3$	$[M-H]^-$	494.9389	8.9, 9.0, 9.2, 9.6
m/z 497 (PFOS − F + OH)	$C_8F_{16}SO_4H_2$	$[M-H]^-$	496.9346	9.4, 9.6, 9.8, 10.0, 10.5
m/z 381 (PFHxS − F + H)	$C_6F_{12}SO_3H_2$	$[M-H]^-$	380.9460	9.0, 9.2, 9.4, 9.7
m/z 281 (PFBS − F + H)	$C_4F_8SO_3H_2$	$[M-H]^-$	280.9524	7.1
m/z 231 (PFPrS − F + H)	$C_3F_6SO_3H_2$	$[M-H]^-$	230.9556	4.8

Table S2. Release of PFAS during the treatment of uncontaminated tap water for 60 min.

Treatment time (min)	***Perfluorocarboxylic acids (ppt)***				
	**PFBA**	**PFPeA**	**PFHxA**	**PFHpA**	**PFOA**
**0**	1.7 ± 0.1	n.d.	4.3 ± 0.1	n.d.	n.d.
**60**	23.2 ± 2.4	12.8 ± 2.8	5.8 ± 0.8	1.5 ± 0.3	3.7 ± 0.6

Treatment time (min)	***Hydro-de-fluorination products (ppt)***				***Hydroxy-de-fluorination products (ppt)***	
	**C8-Carboxylic**	**C7-Carboxylic**	**C6-Carboxylic**	**C5-Carboxylic**	**C7-Carboxylic**	**C6-Carboxylic**
**0**	n.d.	n.d.	n.d.	n.d.	n.d.	n.d.
**60**	5.6 ± 1.0	16.6 ± 7.2	25.4 ± 12.6	34.0 ± 16.4	1.5 ± 1.7	2.7 ± 3.1

Figures

Figures S1-S6. Effect of the temperature of the source and of sheath gas and effect of the voltages of Skimmer, Octapole and Fragmentor voltages on peaks area of selected ions.

Figure S1. Effect of the ESI source temperature (with Sheath gas temperature 350°C, Skimmer Voltage 65 V, Octapole Voltage 750 V, Fragmentor 115 V) on the peak area of the signals relative to (a) PFOA, (b) PFBA, (c) (PFOA – 2F + 2OH) (m/z 409, RT = 8.01 min) and (d) (PFHxA - F + H) (m/z 295, RT = 8.84 min).

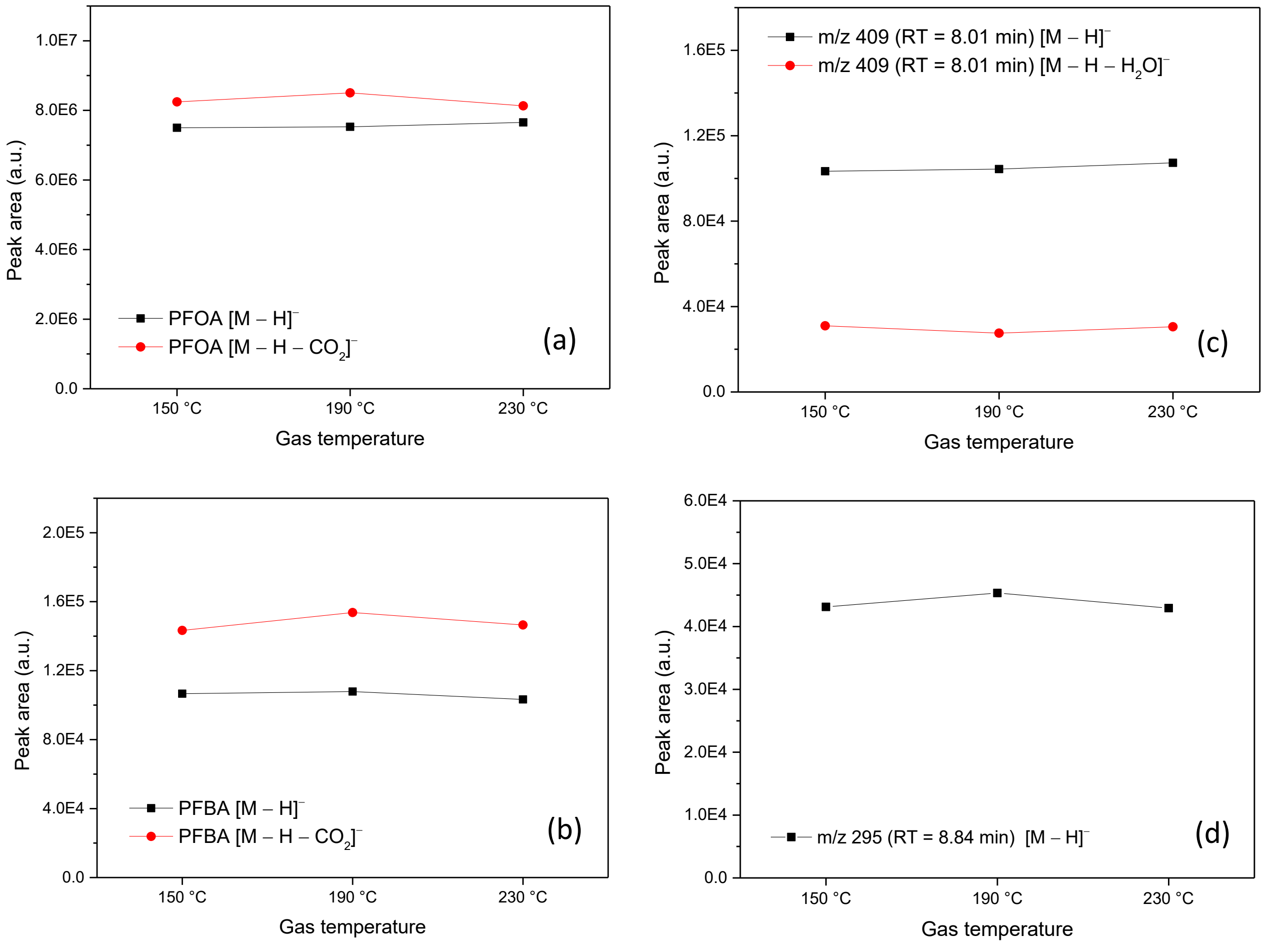

Figure S2. Effect of the Sheath gas temperature (with ESI source temperature 230°C, Skimmer Voltage 65 V, Octapole Voltage 750 V, Fragmentor 115 V) on the peak area of the signals relative to (a) PFOA, (b) PFBA, (c) (PFOA − 2F + 2OH) (m/z 409, RT = 8.01 min) and (d) (PFHxA - F + H) (m/z 295, RT = 8.84 min).

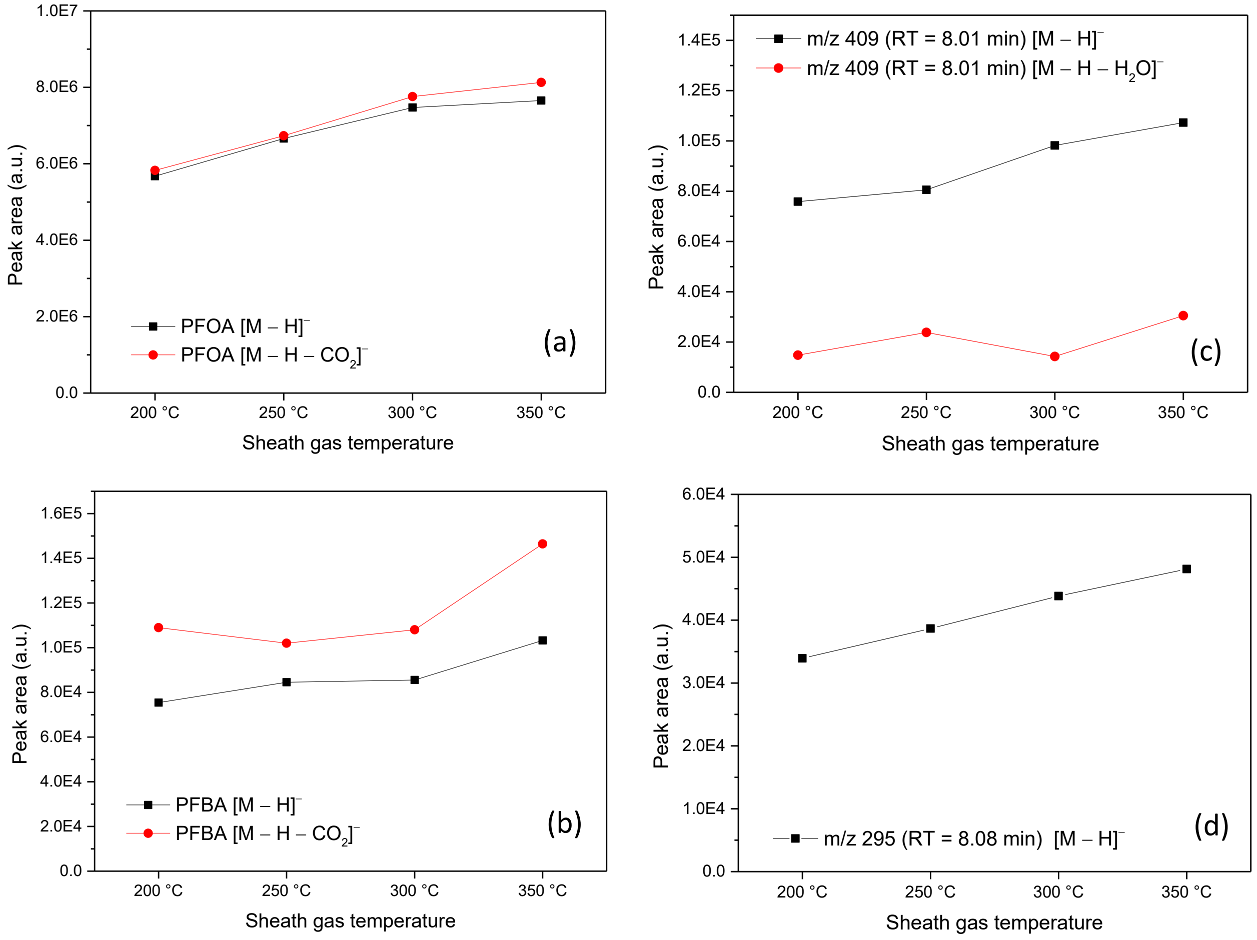

Figure S3. Effect of the Skimmer voltage (with ESI source temperature 230°C, Sheath gas temperature 350°C, Octapole Voltage 750 V, Fragmentor 115 V) on the peak area of the signals relative to (a) PFOA, (b) PFHxA, (c) PFBA, (d) (PFOA – 2F + 2OH) (m/z 409, RT = 8.01 min) and (e) (PFHxA - F + H) (m/z 295, RT = 8.84 min).

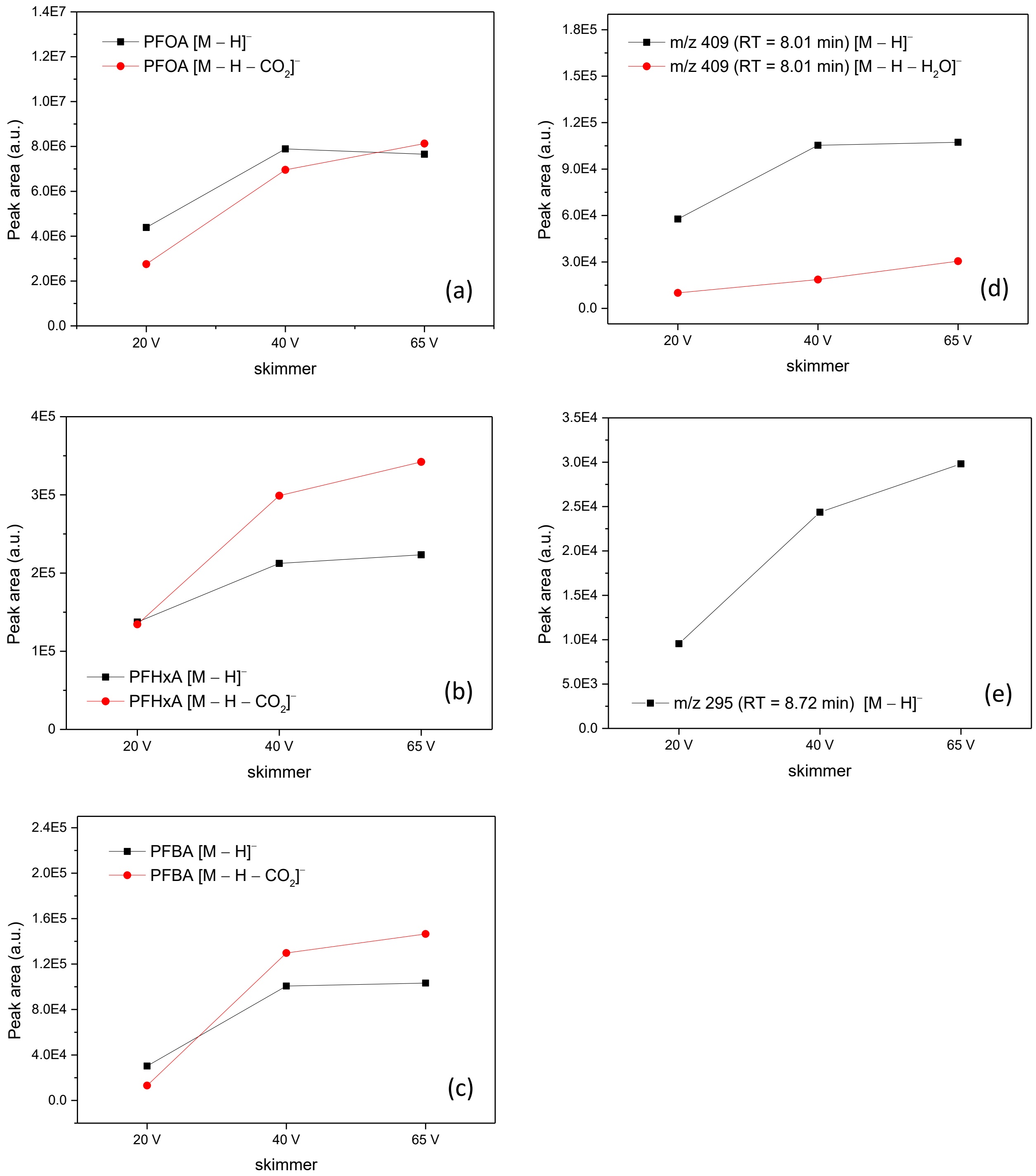

Figure S4. Effect of the Octapole voltage (with ESI source temperature 230°C, Sheath gas temperature 350°C, Skimmer Voltage 65 V, Fragmentor 115 V) on the peak area of the signals relative to (a) PFOA, (b) PFHxA, (c) PFBA, (d) (PFOA – 2F + 2OH) (m/z 409, RT = 8.01 min) and (e) (PFHxA - F + H) (m/z 295, RT = 8.84 min).

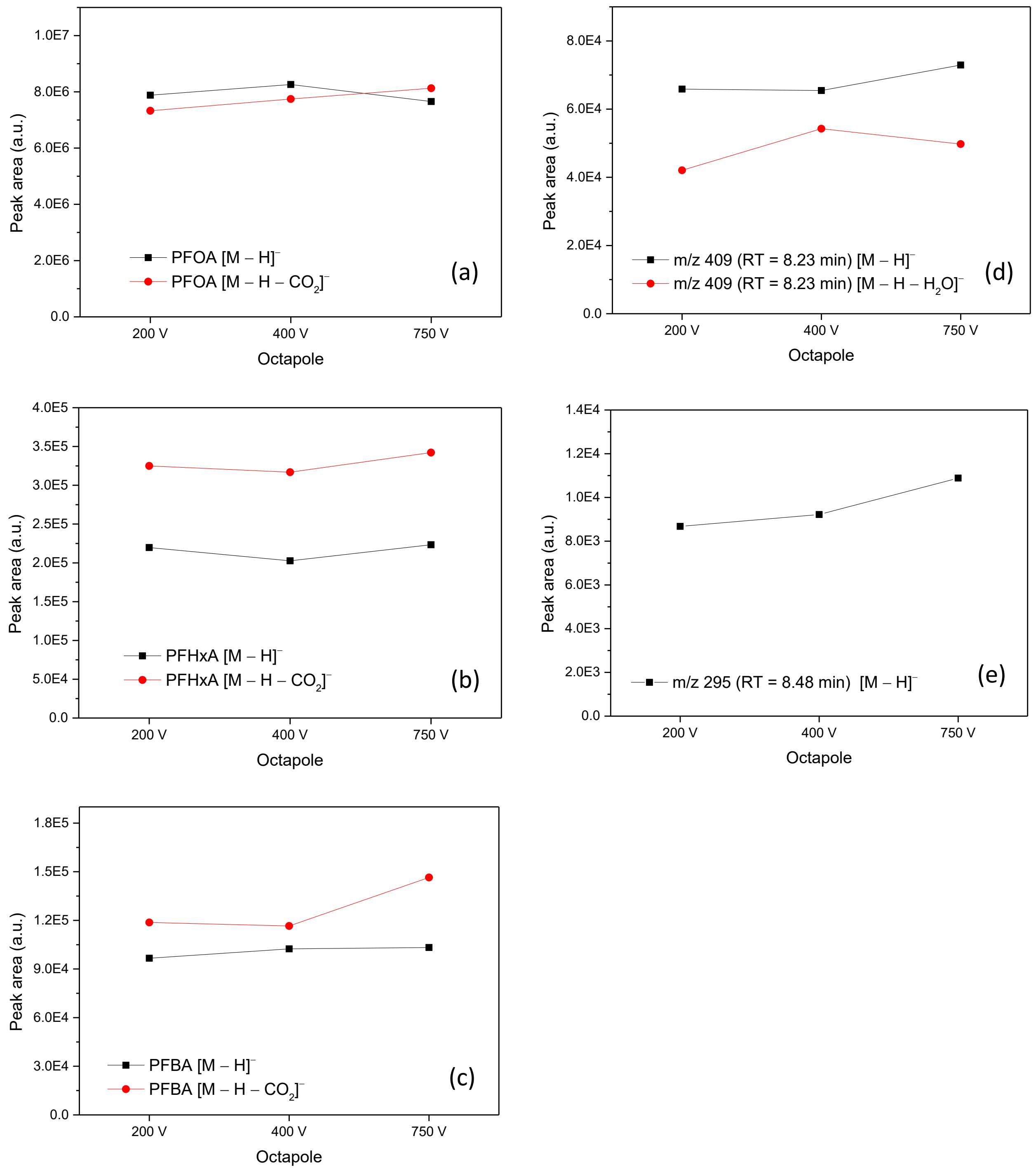

Figure S5. Effect of the Fragmentor voltage (with ESI source temperature 230°C, Sheath gas temperature 350°C, Skimmer Voltage 65 V, Octapole Voltage 750 V) on the peak area of the signals relative to (a) PFOA, (b) PFHxA, (c) PFBA, (d) (PFOA – 2F + 2OH) (m/z 409, RT = 8.01 min) and (e) (PFHxA - F + H) (m/z 295, RT = 8.84 min).

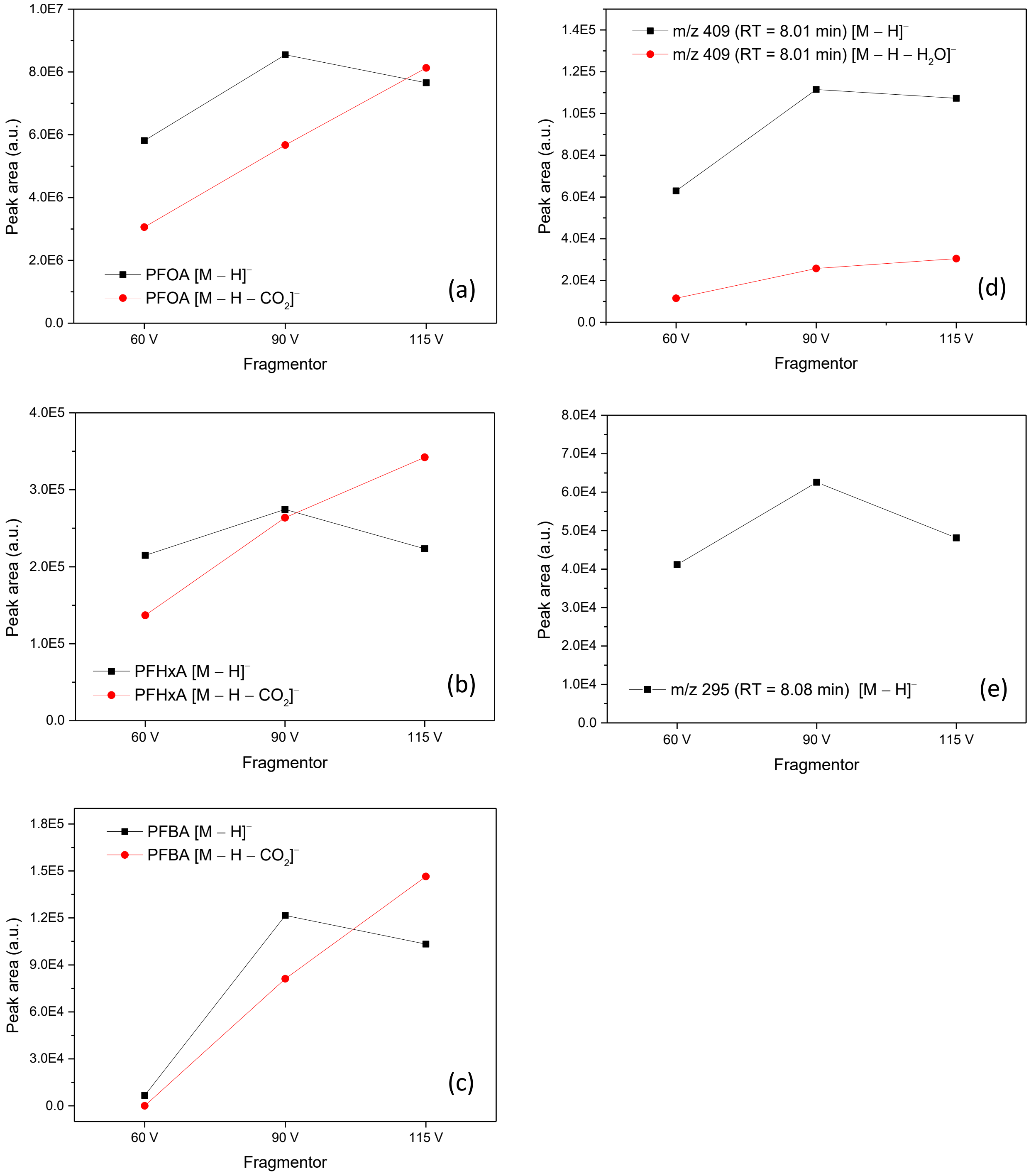

Figure S6. Comparison of the area of the peaks corresponding to deprotonated and decarboxylated PFHpA obtained by setting the voltage of the Fragmentor to 90 and 115 V: (a) chromatograms and (b) bar graph.

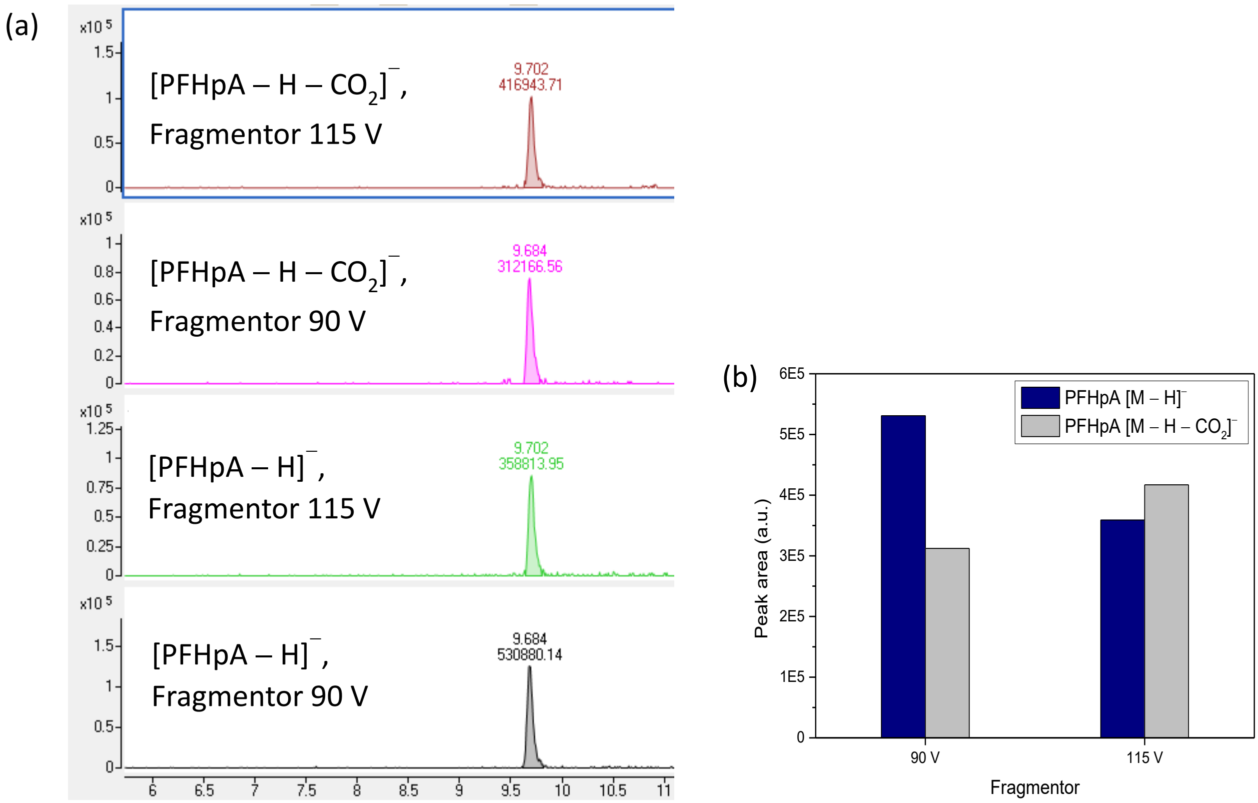

Figures S7-S22. EIC relative to [M – H]$^-$, MS and available MS/MS spectra of the substitution products detected in the analysis of the mixture of the solutions treated with the RAP reactor for 5 minutes, one initially containing PFOA 1.0·10^{-6} M in tap water, the other PFOS 1.0·10^{-6} M in tap water.

Fragmentation energy in MS/MS spectra was 10 V except where differently specified.

Figure S7. EIC, MS and MS/MS spectra of m/z 408.9751

$C_7F_{13}(OH)_2\text{-}COO^-$

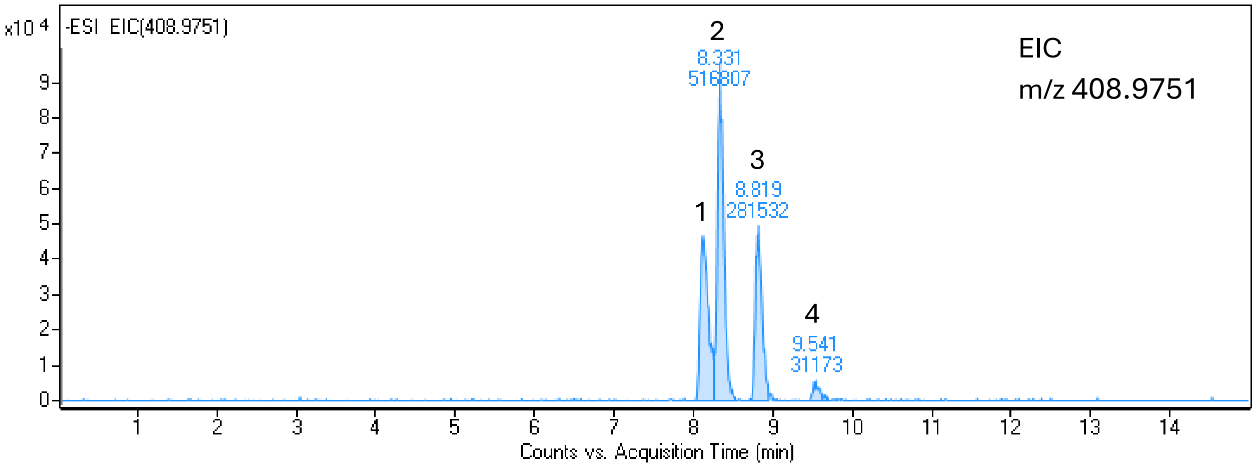

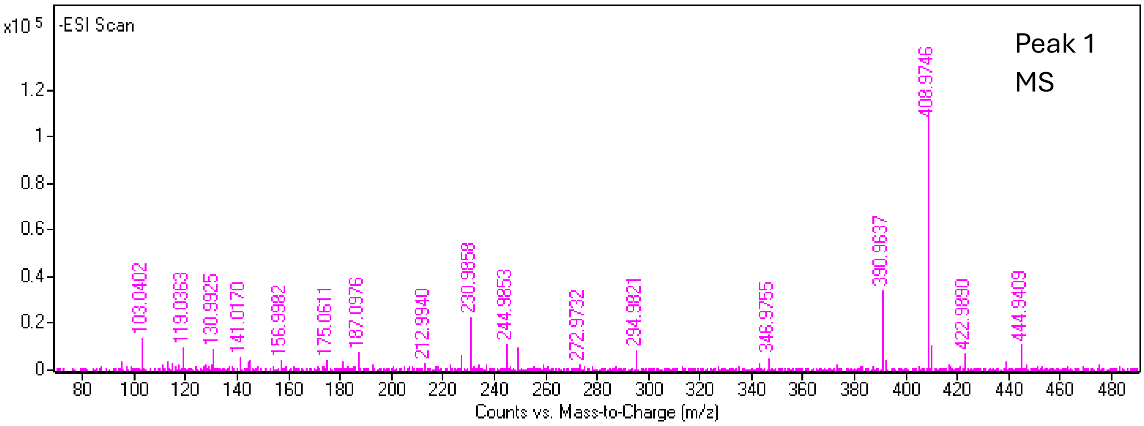

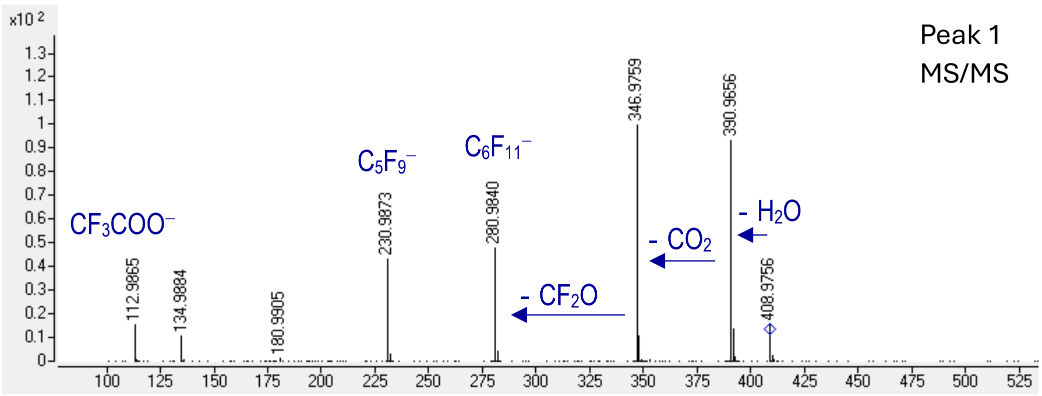

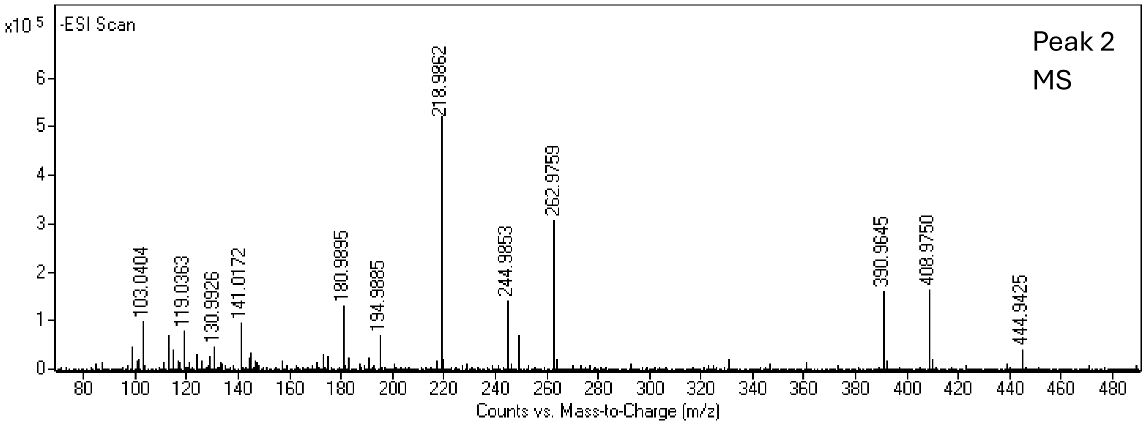

x10 5
-ESI Scan
Peak 2
MS
103.0404
119.0363
130.9926
141.0172
180.9895
194.9885
218.9862
244.9853
262.9759
390.9645
408.9750
444.9425
Counts vs. Mass-to-Charge (m/z)

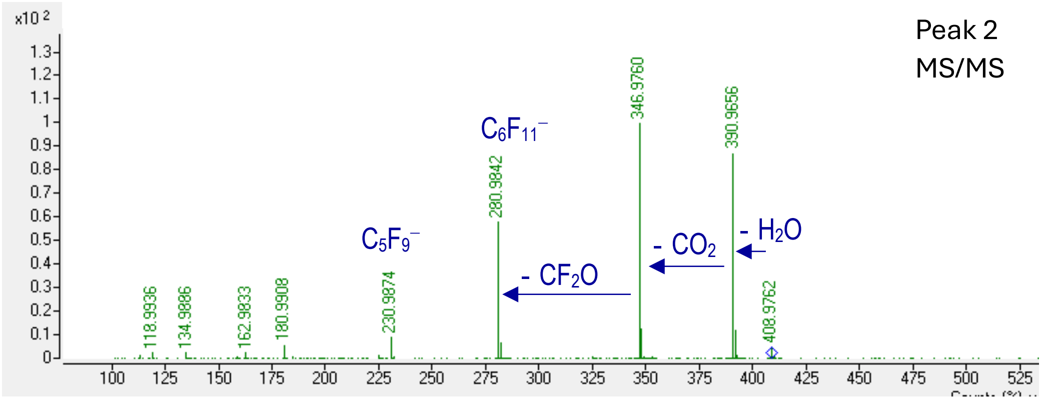

x10 2
Peak 2
MS/MS
118.9936
134.9886
162.9833
180.9908
230.9874
280.9842
346.9760
390.9656
408.9762
$C_5F_9^-$
$C_6F_{11}^-$
$- CF_2O$
$- CO_2$
$- H_2O$

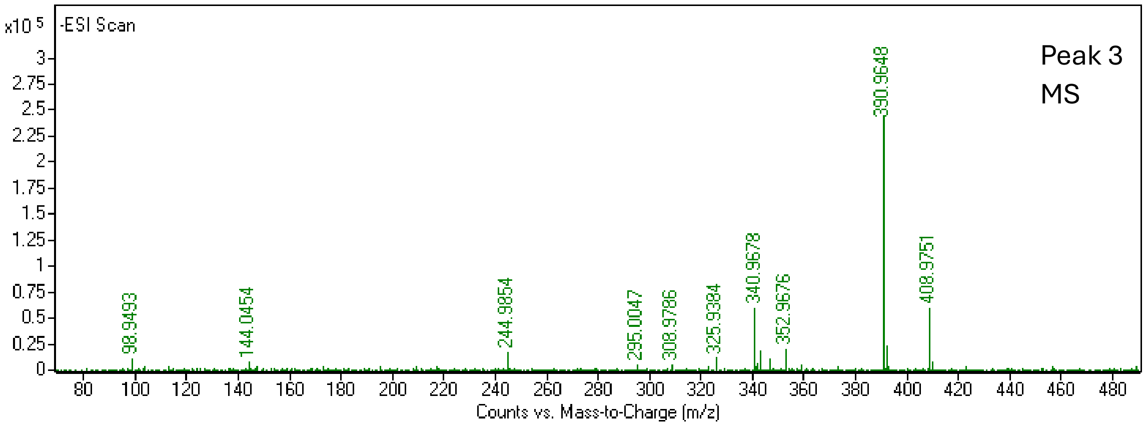

x10 5
-ESI Scan
Peak 3
MS
98.9493
144.0454
244.9854
295.0047
308.9786
325.9384
340.9678
352.9676
390.9648
408.9751
Counts vs. Mass-to-Charge (m/z)

Figure S8. EIC, MS and MS/MS spectra of m/z 358.9783

$C_6F_{11}(OH)_2\text{-}COO^-$

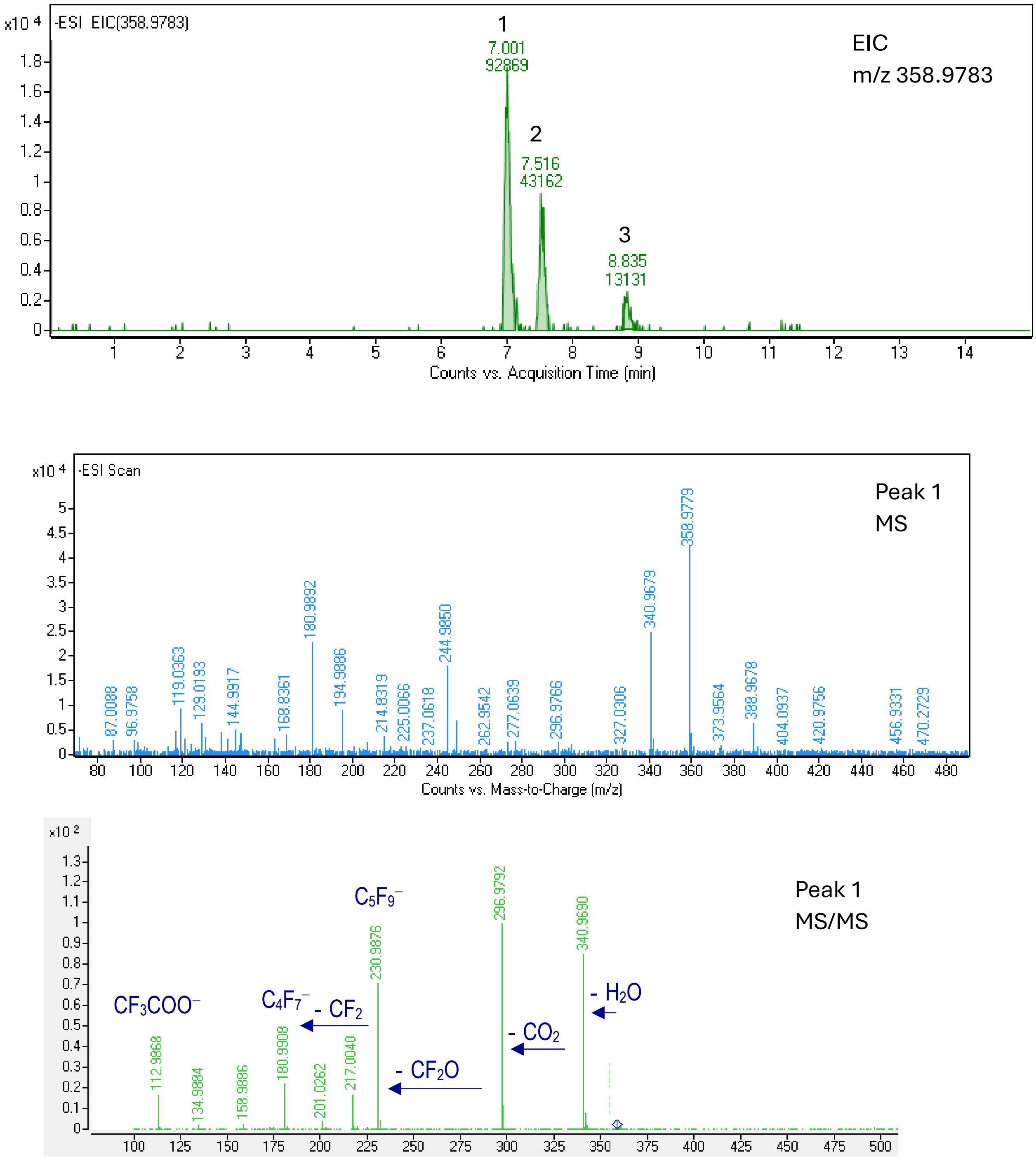

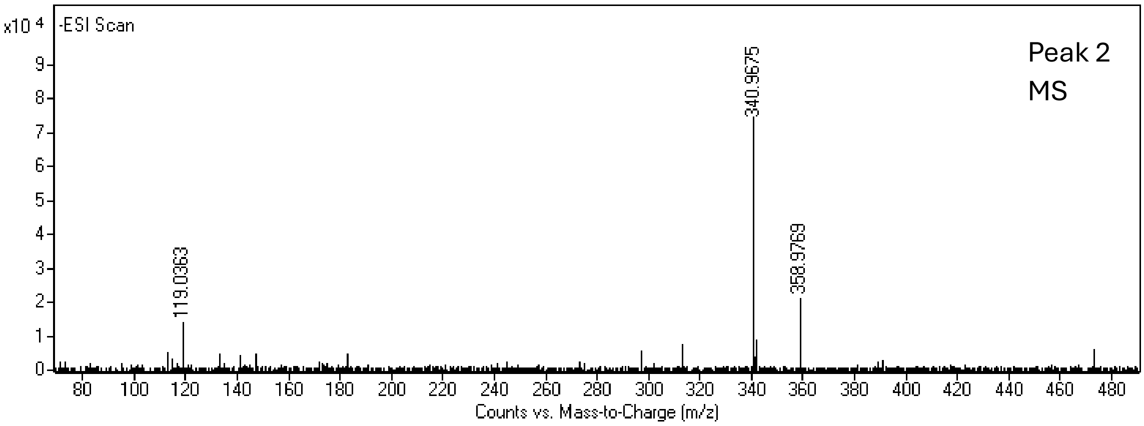

x10 4
-ESI Scan
Peak 2
MS
340.9675
358.9769
119.0363
Counts vs. Mass-to-Charge (m/z)

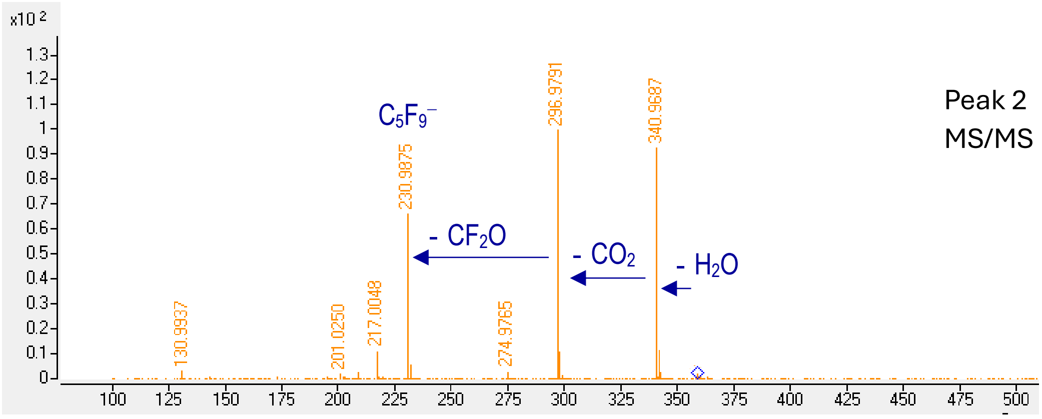

x10 2
Peak 2
MS/MS
$C_5F_9^-$
296.9791
340.9687
230.9875
- CF_2O
- CO_2
- H_2O
130.9937
201.0250
217.0048
274.9765

Figure S9. EIC, MS and MS/MS spectra of m/z 308.9815

$C_5F_9(OH)_2\text{-}COO^-$

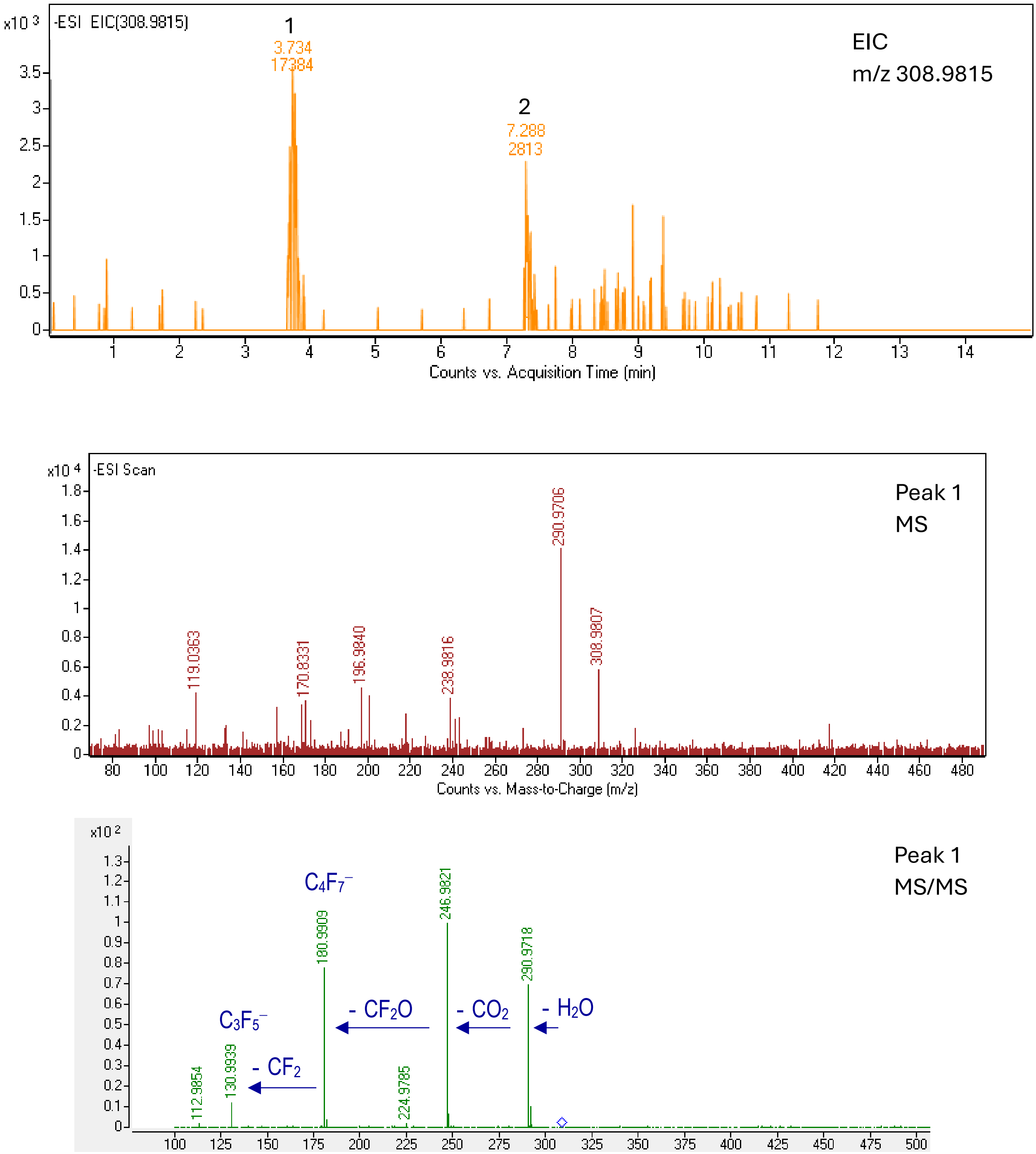

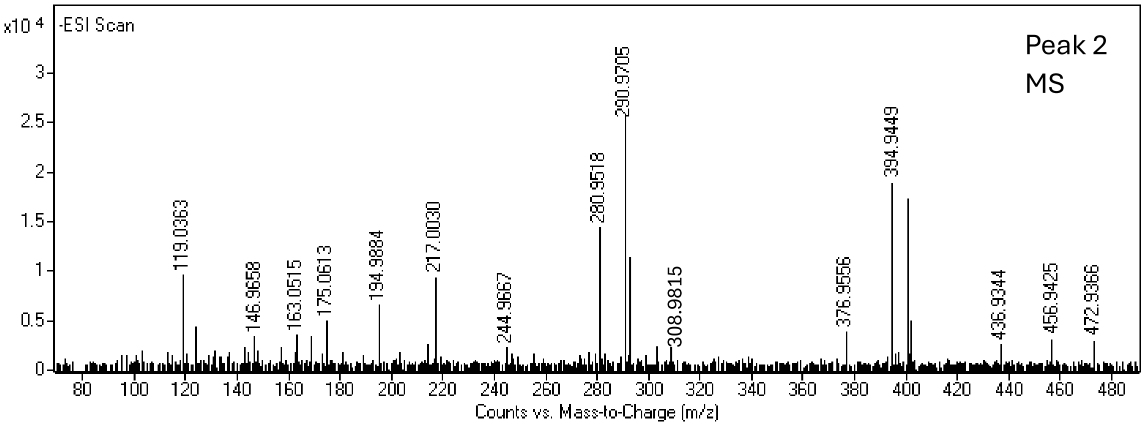

x10 4
-ESI Scan
Peak 2
MS
119.0363
146.9658
163.0515
175.0613
194.9884
217.0030
244.9667
280.9518
290.9705
308.9815
376.9556
394.9449
436.9344
456.9425
472.9366
Counts vs. Mass-to-Charge (m/z)

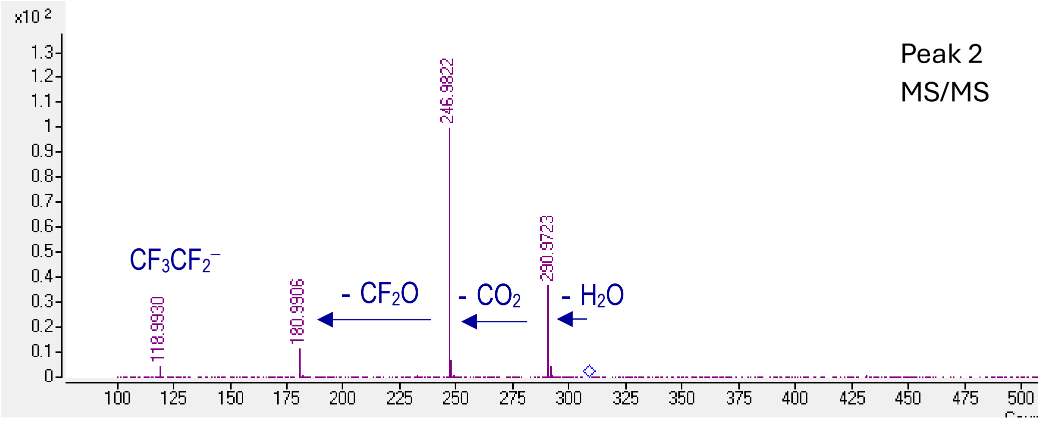

x10 2
Peak 2
MS/MS
CF3CF2−
118.9930
180.9906
246.9822
290.9723
- CF2O
- CO2
- H2O

Figure S10. EIC, MS and MS/MS spectra of m/z 392.9802

$C_7F_{13}H(OH)\text{-}COO^-$

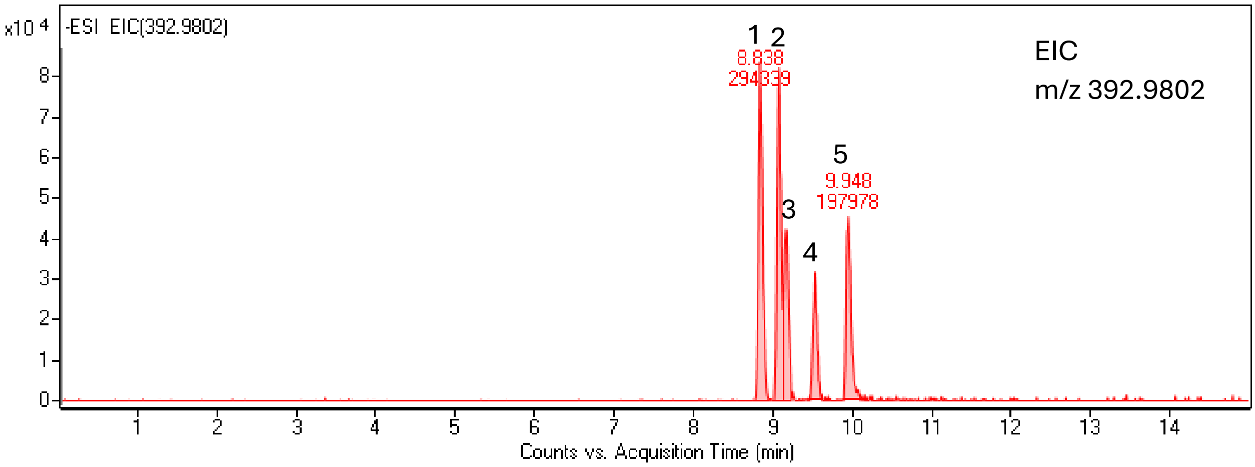

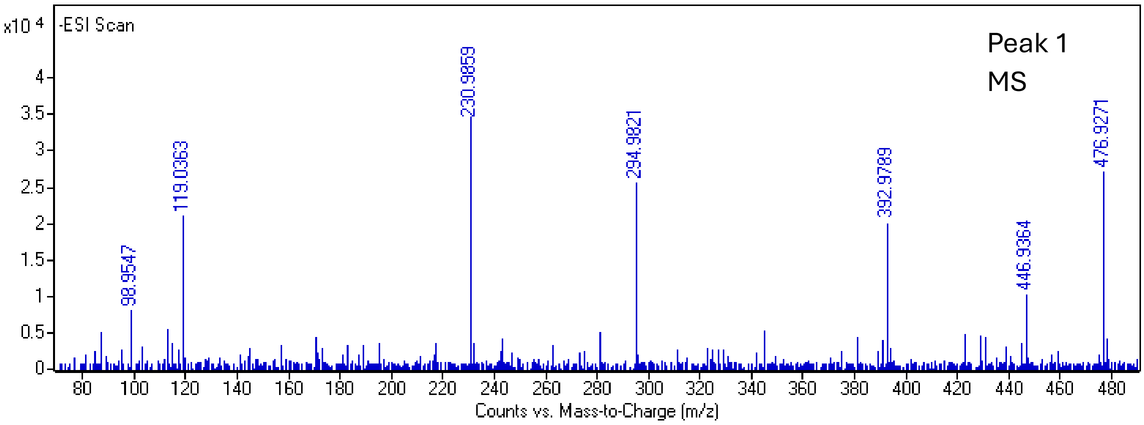

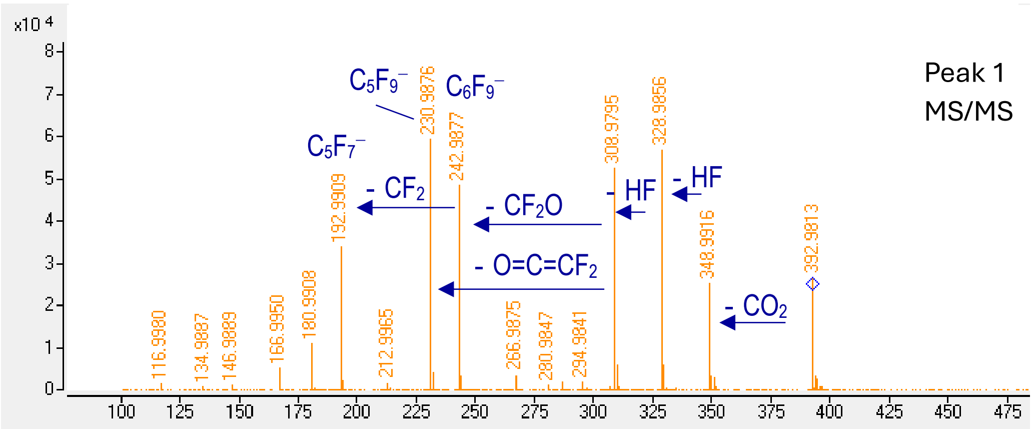

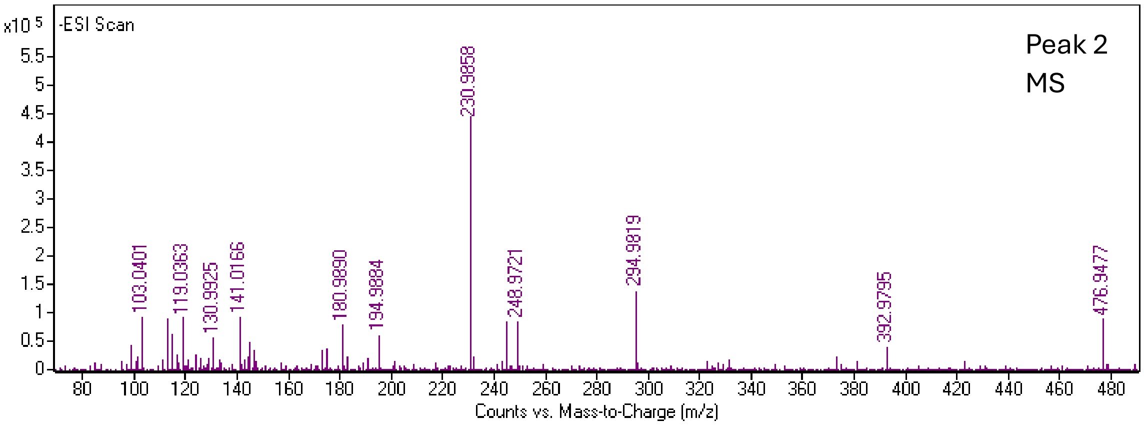
x10 5
-ESI Scan
Peak 2
MS
230.9858
294.9819
103.0401
119.0363
130.9925
141.0166
180.9890
194.9884
248.9721
392.9795
476.9477
Counts vs. Mass-to-Charge (m/z)

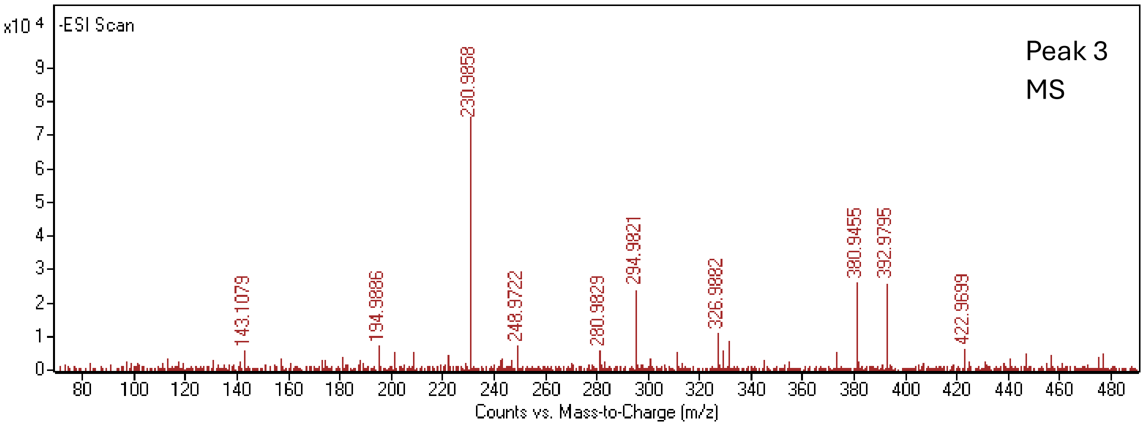
x10 4
-ESI Scan
Peak 3
MS
230.9858
143.1079
194.9886
248.9722
280.9829
294.9821
326.9882
380.9455
392.9795
422.9639
Counts vs. Mass-to-Charge (m/z)

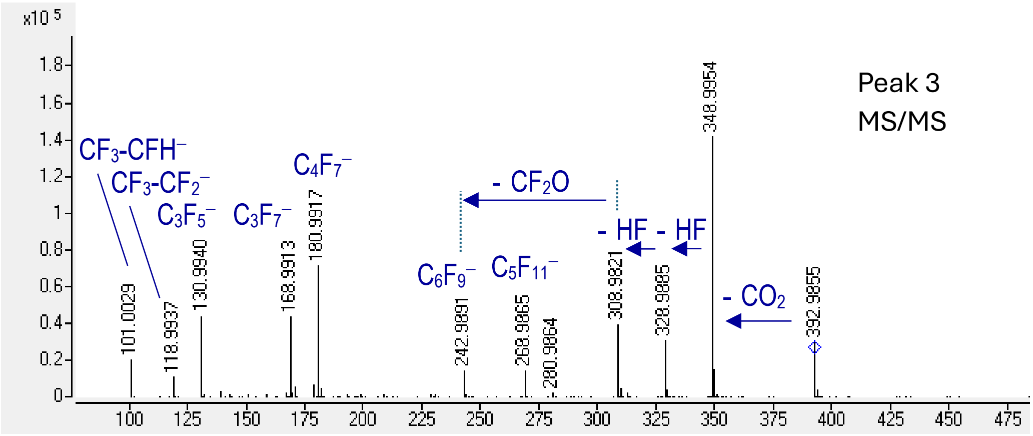
x10 5
Peak 3
MS/MS
348.9954
CF3-CFH−
CF3-CF2−
C3F5−
C3F7−
C4F7−
- CF2O
- HF - HF
C6F9−
C5F11−
- CO2
101.0029
118.9937
130.9940
168.9913
180.9917
242.9891
268.9865
280.9864
308.9821
328.9885
392.9855

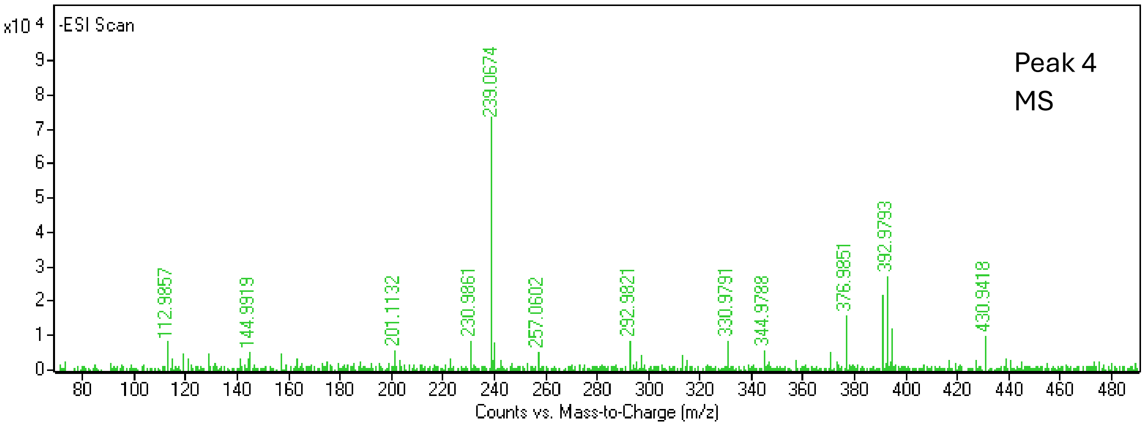
x10 4
-ESI Scan
Peak 4
MS
112.9857
144.9919
201.1132
230.9861
239.0674
257.0602
292.9821
330.9791
344.9788
376.9851
392.9793
430.9418
Counts vs. Mass-to-Charge (m/z)

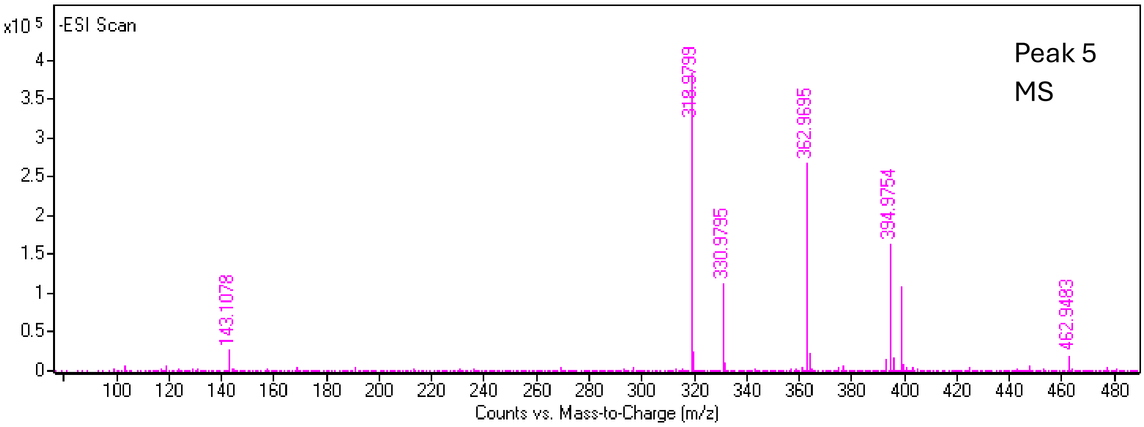
x10 5
-ESI Scan
Peak 5
MS
143.1078
318.9799
330.9795
362.9695
394.9754
462.9483
Counts vs. Mass-to-Charge (m/z)

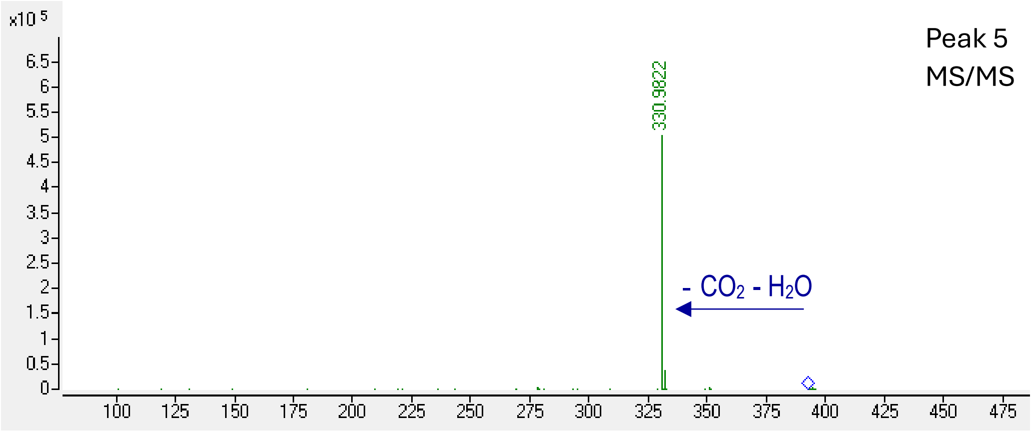
x10 5
Peak 5
MS/MS
330.9822
- CO2 - H2O

Figure S11. EIC, MS spectra and MS/MS spectra of m/z 342.9834

$C_6F_{11}H(OH)\text{-}COO^-$

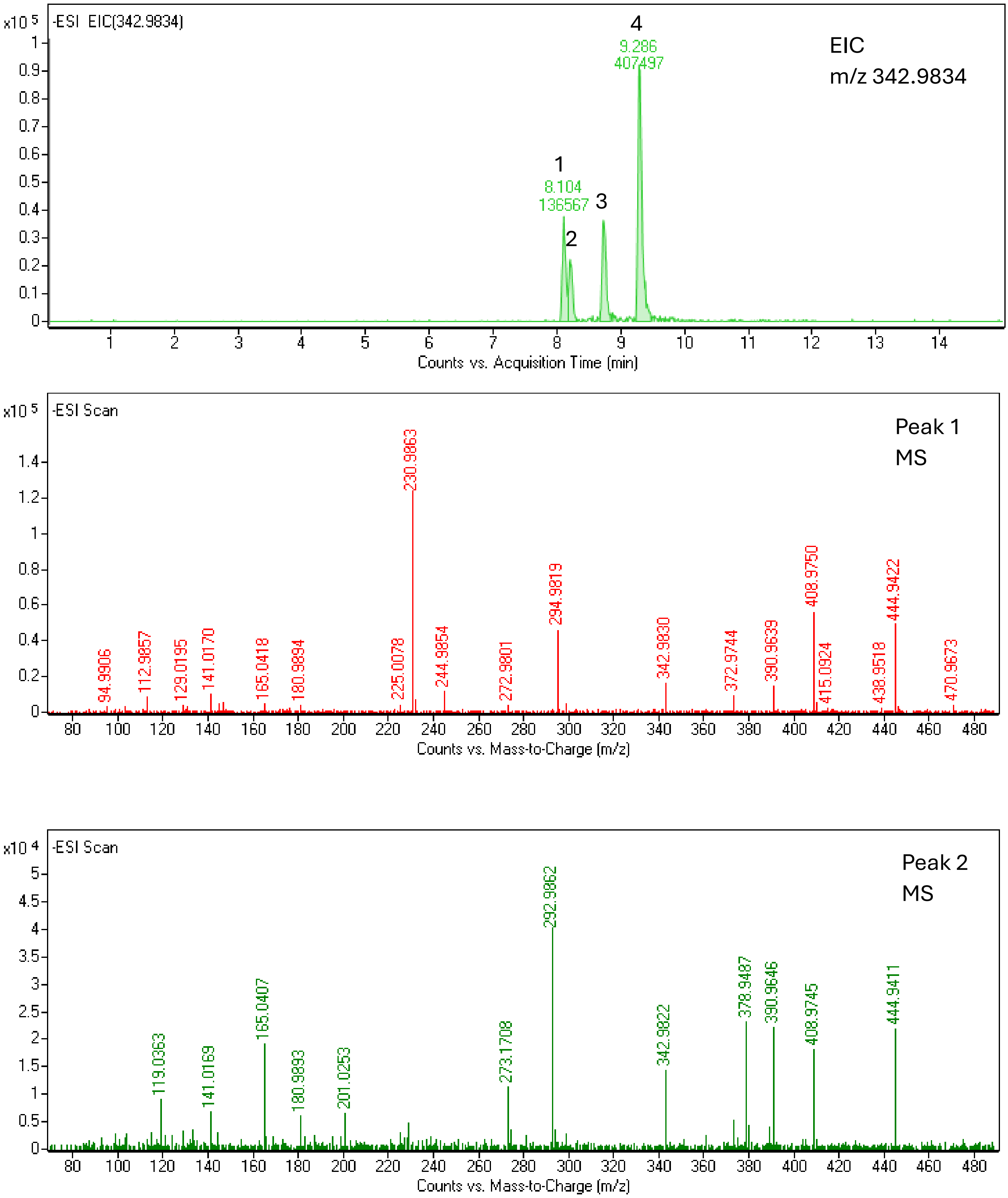

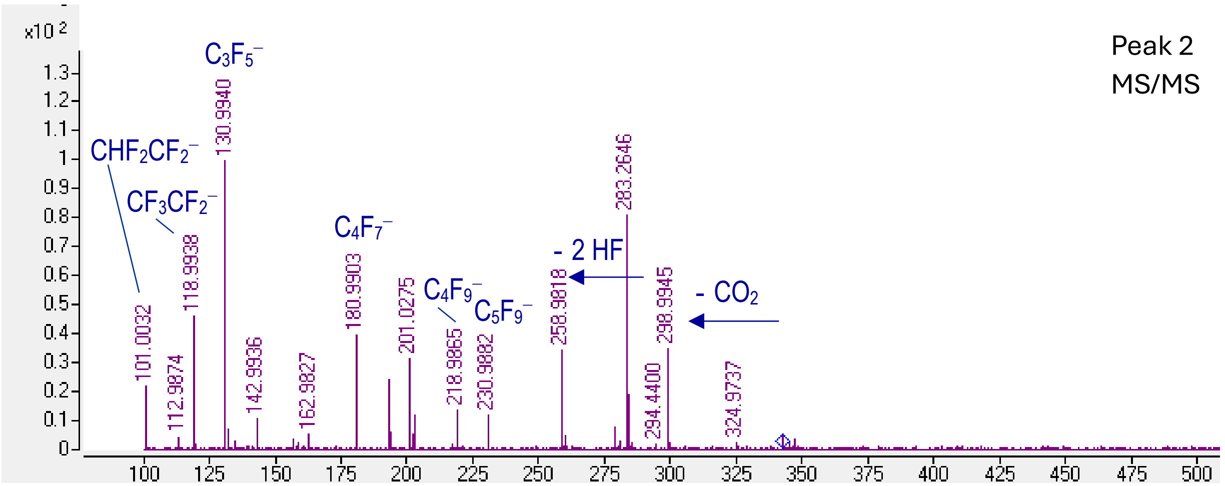
Peak 2
MS/MS
x10 2
$C_3F_5^-$
$CHF_2CF_2^-$
$CF_3CF_2^-$
$C_4F_7^-$
$C_4F_9^-$
$C_5F_9^-$
- 2 HF
- CO_2
101.0032
112.9874
118.9938
130.9940
142.9936
162.9827
180.9903
201.0275
218.9865
230.9882
258.9818
283.2646
294.4400
298.9945
324.9737

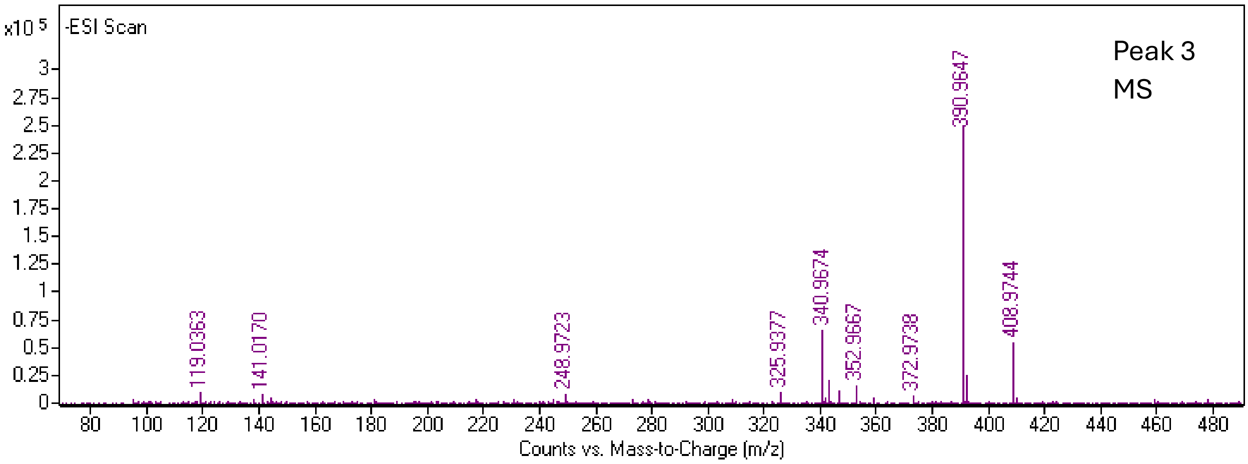
x10 5
-ESI Scan
Peak 3
MS
119.0363
141.0170
248.9723
325.9377
340.9674
352.9667
372.9738
390.9647
408.9744
Counts vs. Mass-to-Charge (m/z)

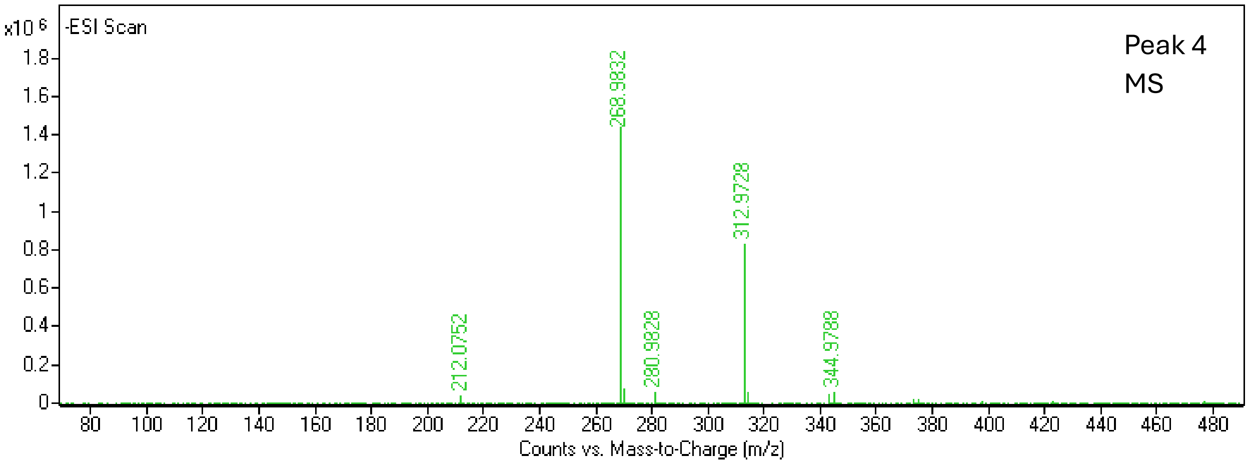
x10 6
-ESI Scan
Peak 4
MS
212.0752
268.9832
280.9828
312.9728
344.9788
Counts vs. Mass-to-Charge (m/z)

Figure S12. EIC, MS and MS/MS spectra of m/z 292.9866

$C_5F_9H(OH)\text{-}COO^-$

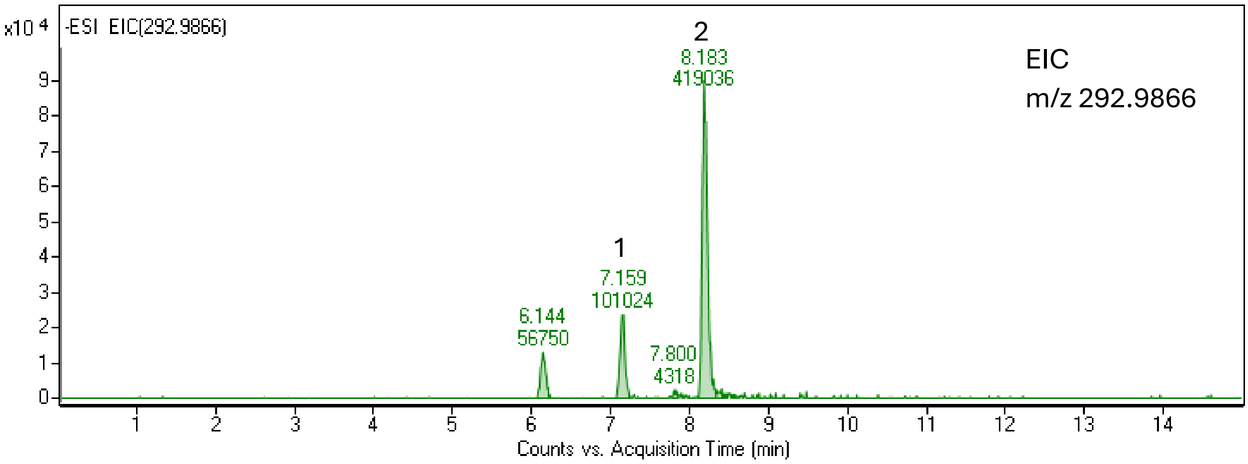

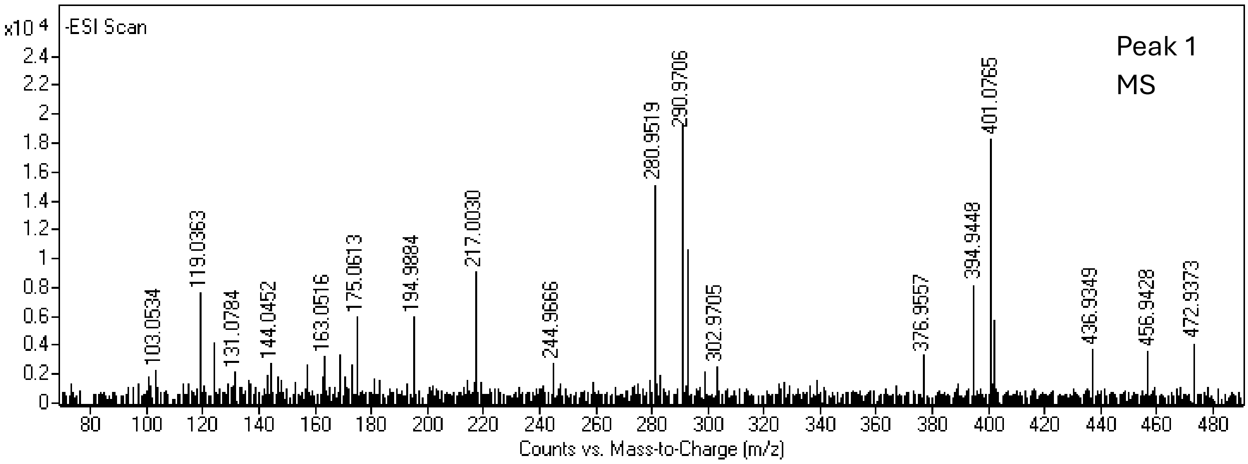

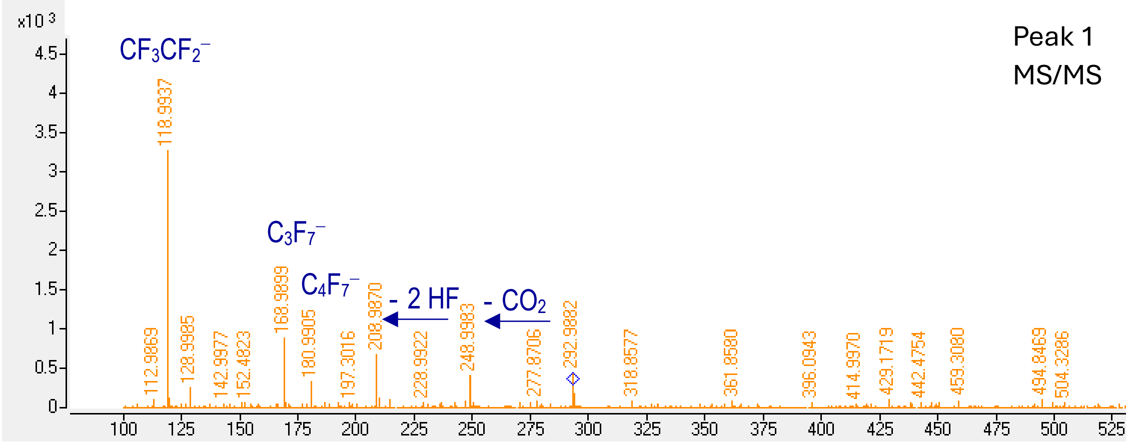

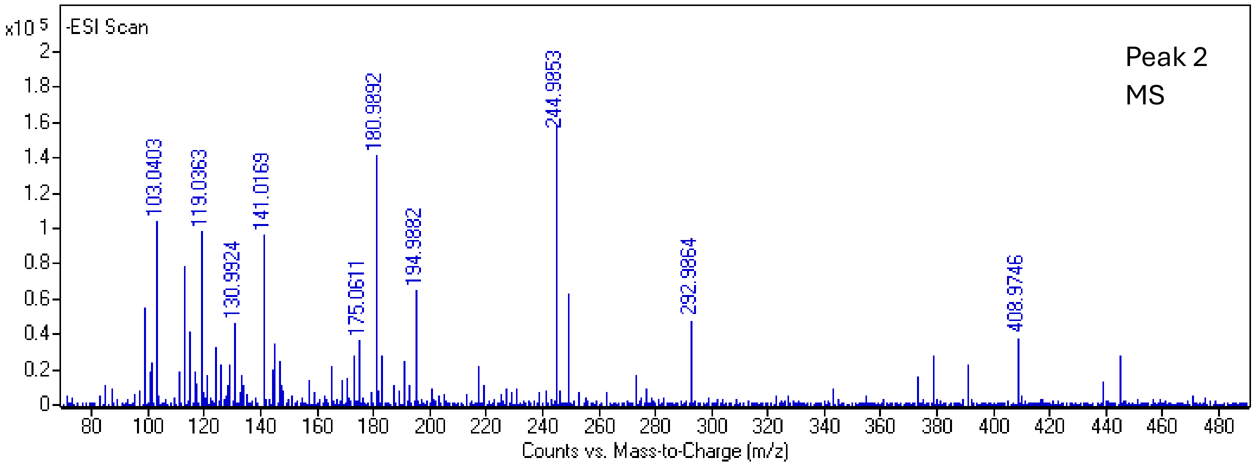
x10 5
-ESI Scan
Peak 2
MS
103.0403
119.0363
130.9924
141.0169
175.0611
180.9892
194.9882
244.9853
292.9864
408.9746
Counts vs. Mass-to-Charge (m/z)

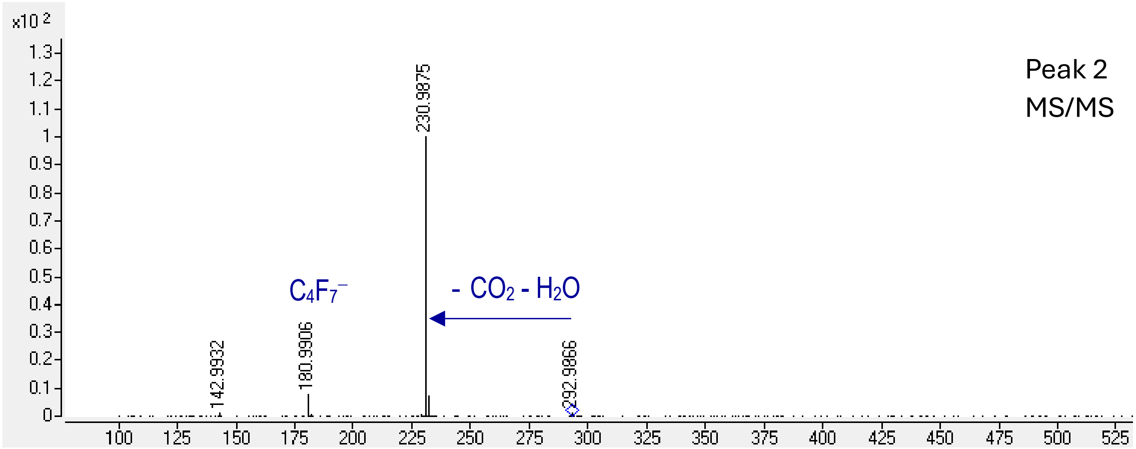
x10 2
Peak 2
MS/MS
230.9875
$C_4F_7^-$
- CO_2 - H_2O
142.9932
180.9906
292.9866

Figure S13. (a) EIC, MS and MS/MS spectra of m/z 242.9898

$C_4F_7H(OH)\text{-}COO^-$

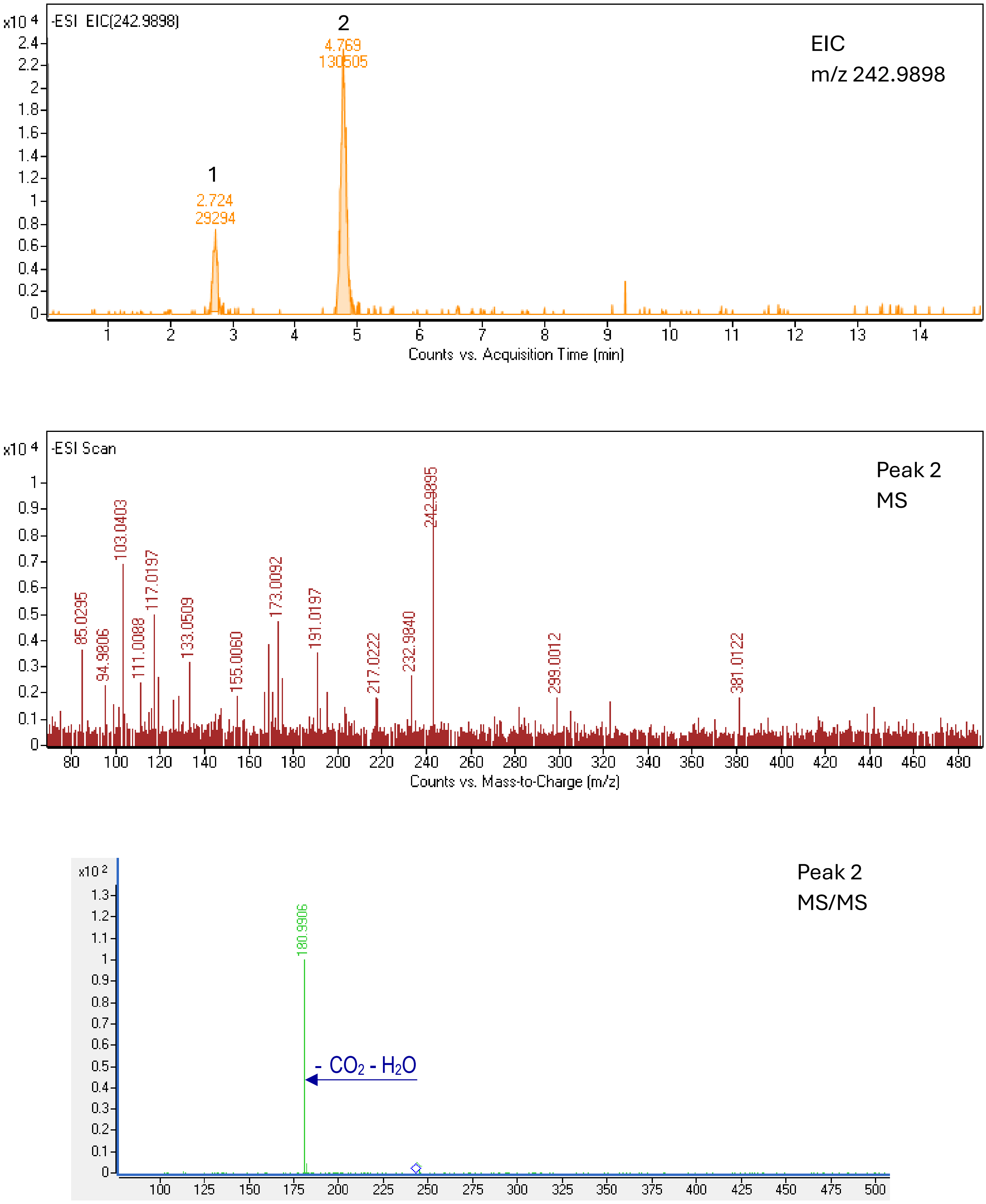

Figure S14. EIC, MS and MS/MS spectra of m/z 394.9758

$C_7F_{14}H\text{-}COO^-$

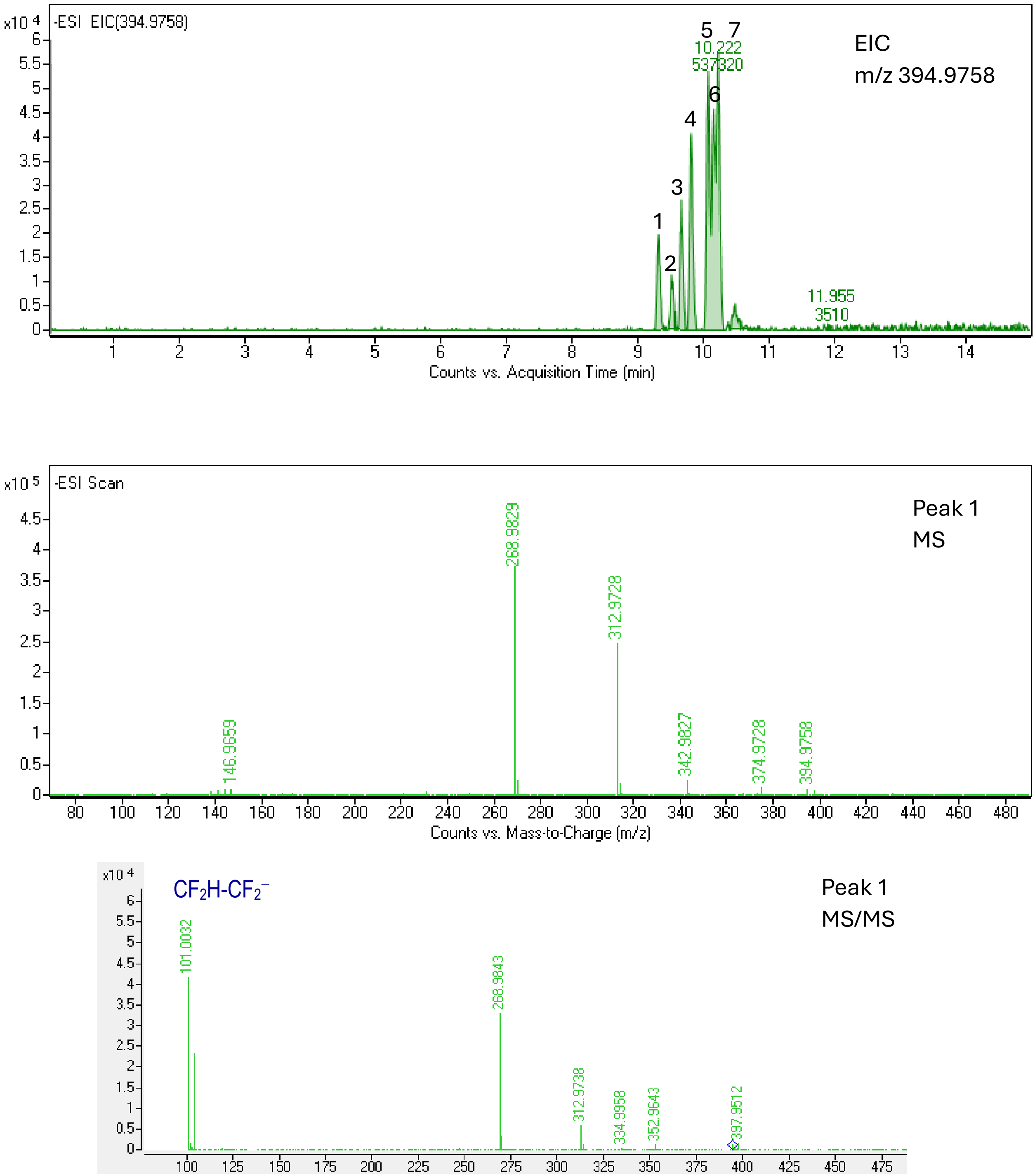

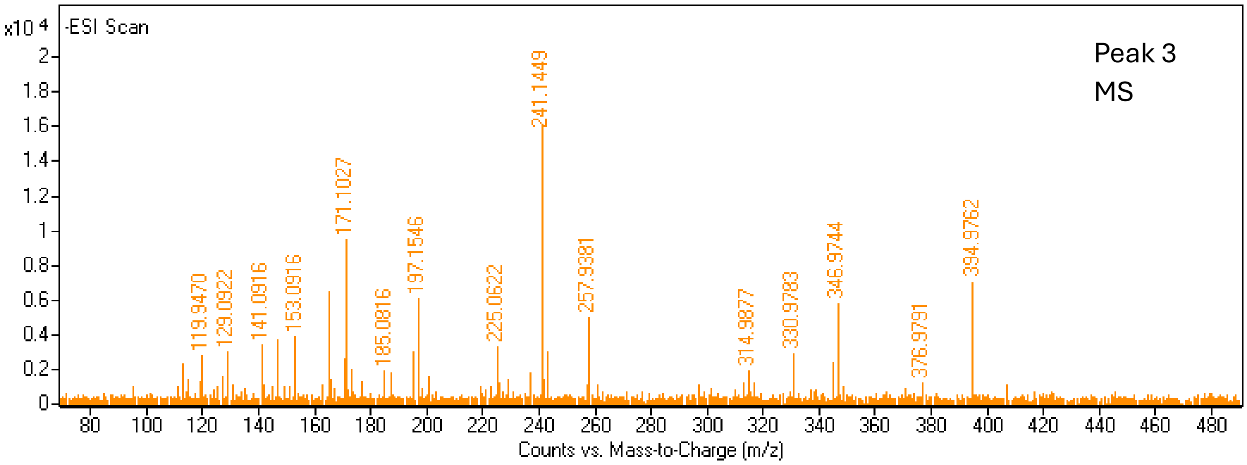
x10 4
-ESI Scan
Peak 3
MS
119.9470
129.0922
141.0916
153.0916
171.1027
185.0816
197.1546
225.0622
241.1449
257.9381
314.9877
330.9783
346.9744
376.9791
394.9762
Counts vs. Mass-to-Charge (m/z)

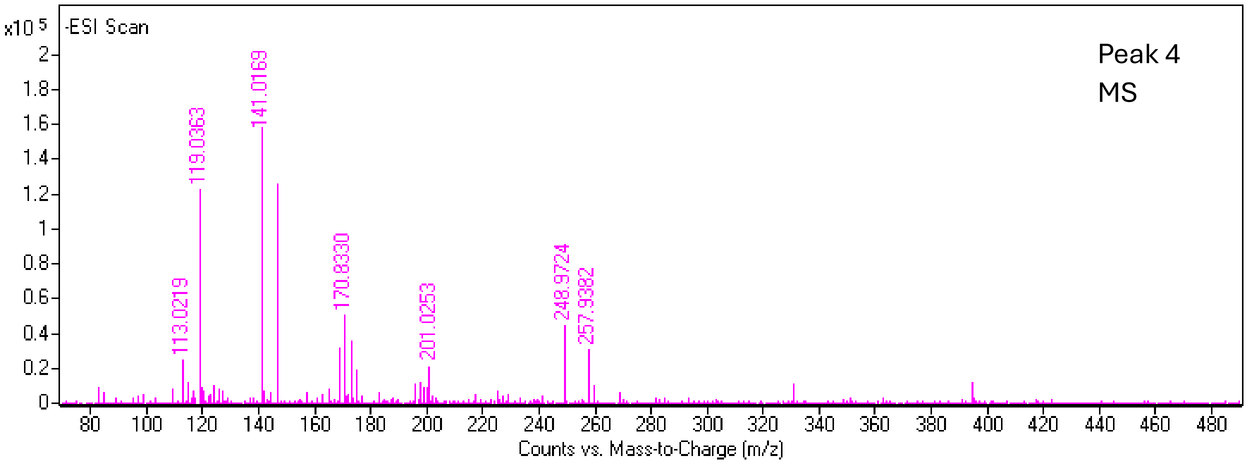
x10 5
-ESI Scan
Peak 4
MS
113.0219
119.0363
141.0169
170.8330
201.0253
248.9724
257.9382
Counts vs. Mass-to-Charge (m/z)

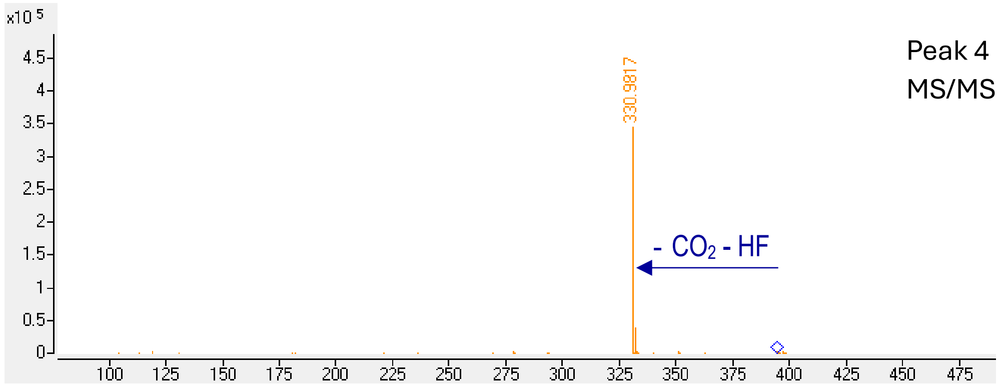
x10 5
Peak 4
MS/MS
330.9817
- CO2 - HF

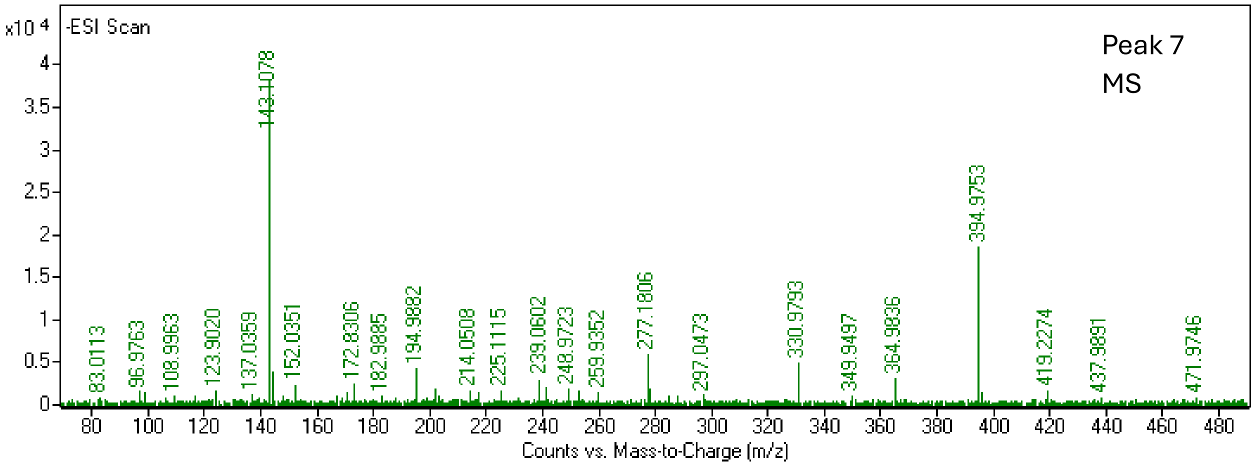

x10 4
-ESI Scan
Peak 7
MS
143.1078
394.9753
83.0113
96.9763
108.9963
123.9020
137.0359
152.0351
172.8306
182.9885
194.9882
214.0508
225.1115
239.0602
248.9723
259.9352
277.1806
297.0473
330.9793
349.9497
364.9836
419.2274
437.9891
471.9746
Counts vs. Mass-to-Charge (m/z)

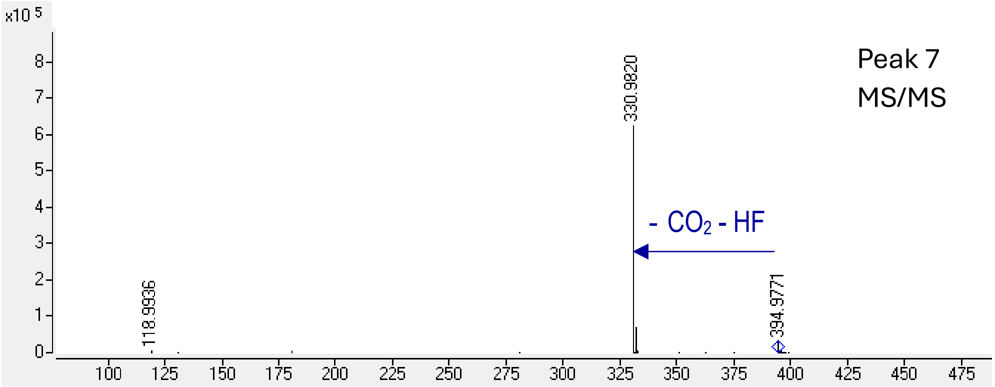

x10 5
Peak 7
MS/MS
330.9820
- CO2 - HF
118.9936
394.9771

Figure S15. EIC, MS and MS/MS spectra of m/z 344.9790

$C_6F_{12}H\text{-}COO^-$

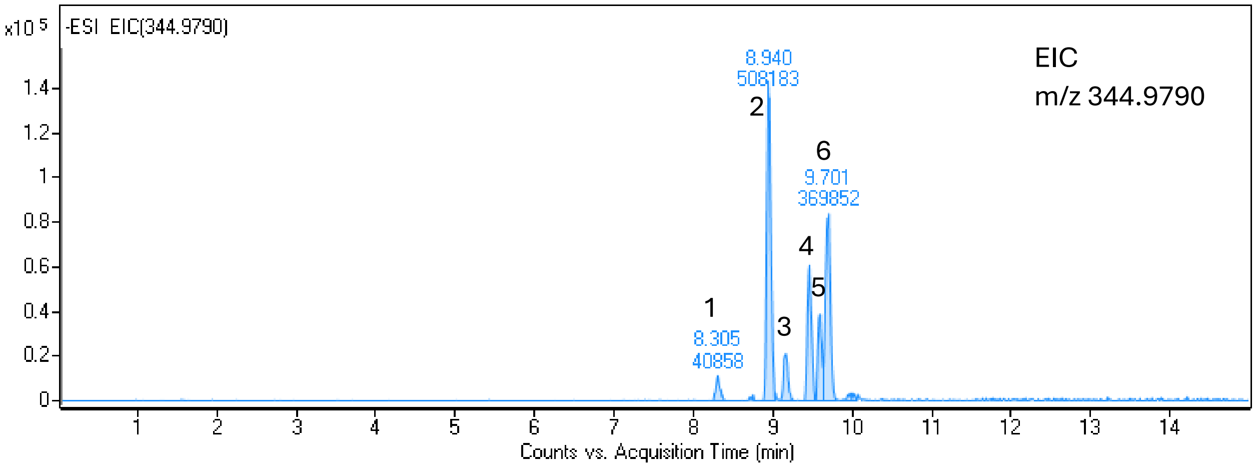

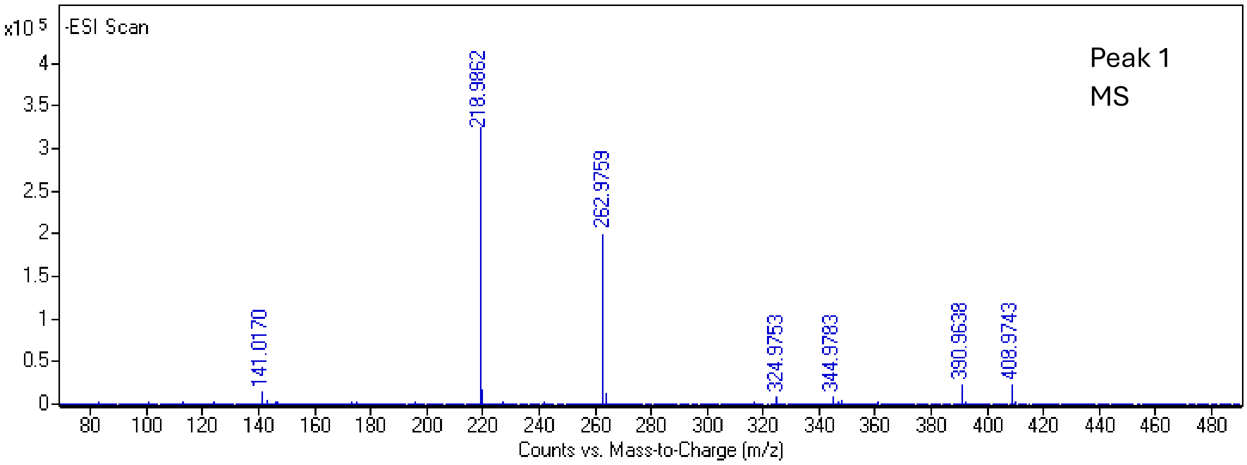

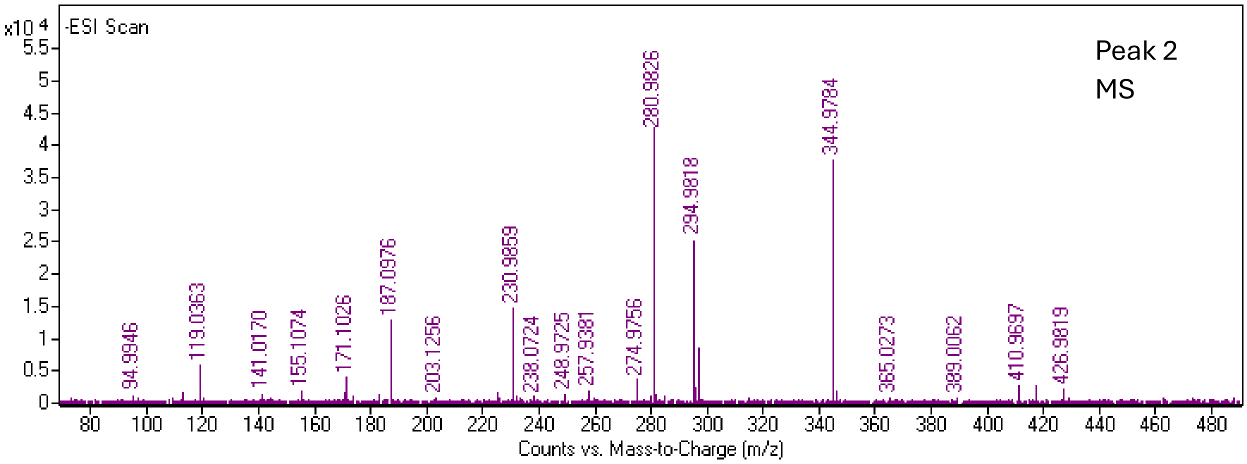

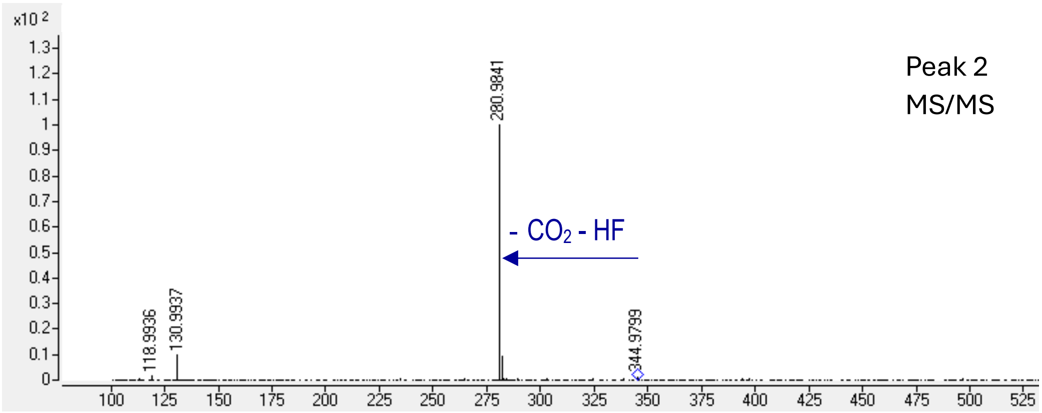
Peak 2
MS/MS
x10 2
- CO2 - HF
280.9841
118.9936
130.9937
344.9799

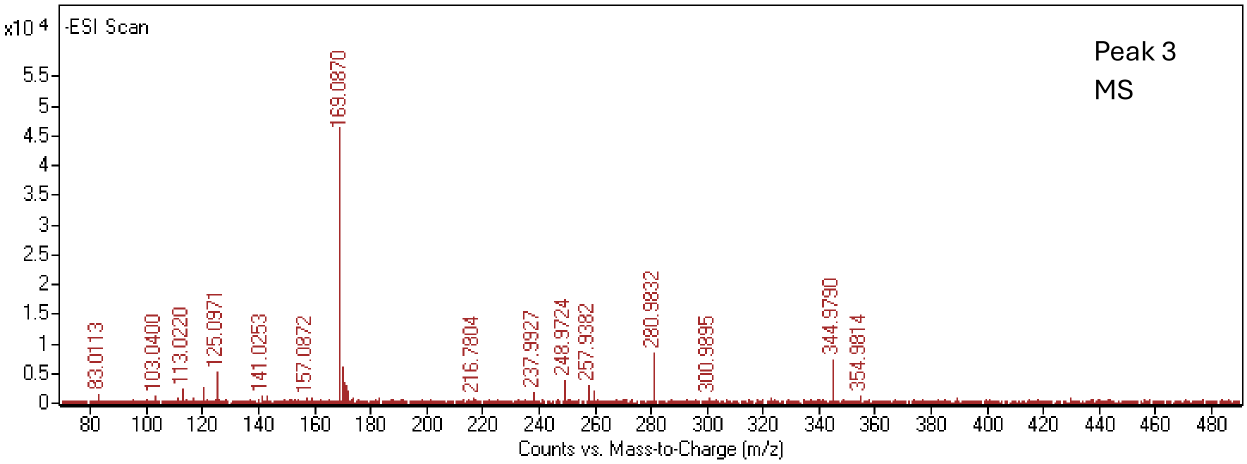
Peak 3
MS
x10 4
-ESI Scan
169.0870
83.0113
103.0400
113.0220
125.0971
141.0253
157.0872
216.7804
237.9927
248.9724
257.9382
280.9832
300.9895
344.9790
354.9814
Counts vs. Mass-to-Charge (m/z)

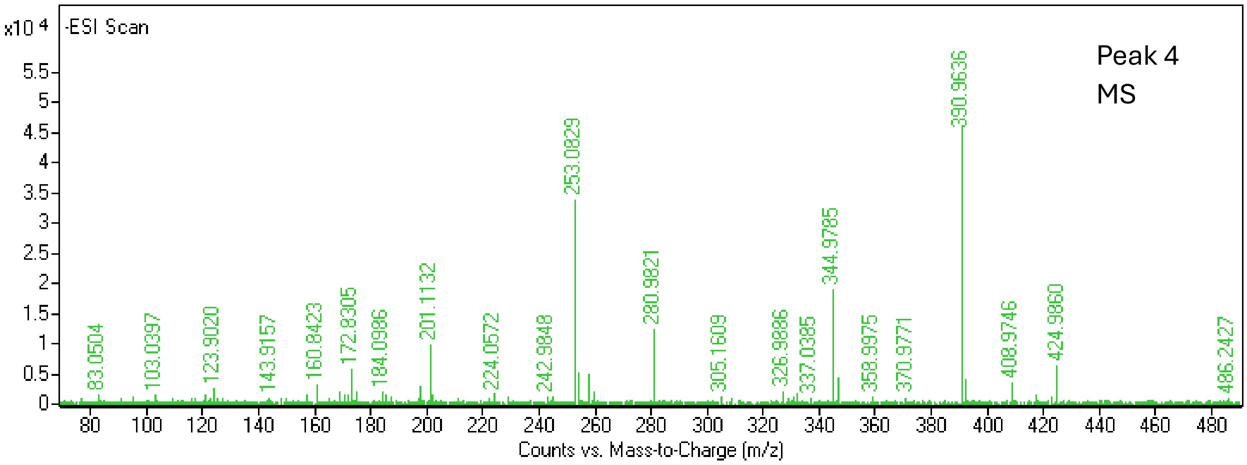
Peak 4
MS
x10 4
-ESI Scan
390.9636
253.0829
83.0504
103.0397
123.9020
143.9157
160.8423
172.8305
184.0986
201.1132
224.0572
242.9848
280.9821
305.1609
326.9886
337.0385
344.9785
358.9975
370.9771
408.9746
424.9860
486.2427
Counts vs. Mass-to-Charge (m/z)

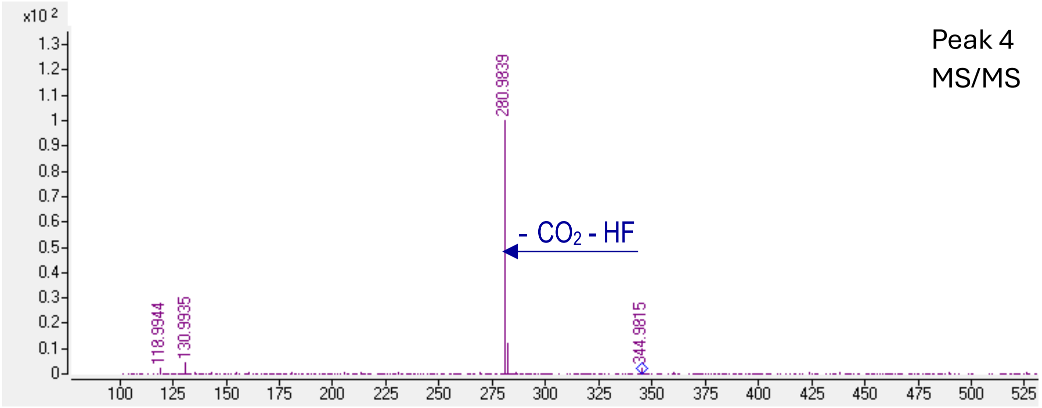
Peak 4
MS/MS
x10 2
- CO2 - HF
280.9839
118.9944
130.9935
344.9815

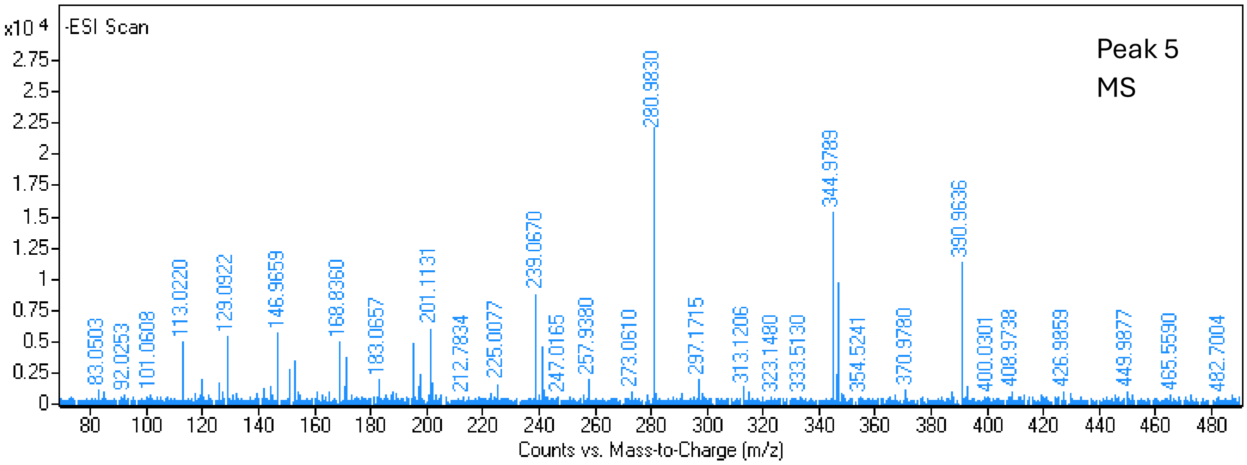
Peak 5
MS
-ESI Scan
x10 4
Counts vs. Mass-to-Charge (m/z)
280.9830
344.9789
390.9636
239.0670
201.1131
168.8360
146.9659
129.0922
113.0220

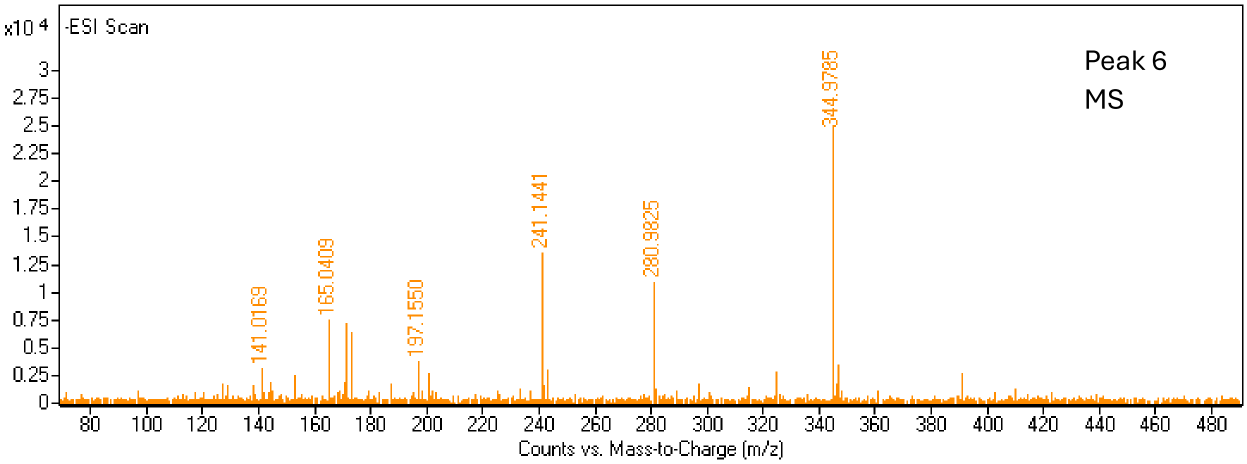
Peak 6
MS
-ESI Scan
x10 4
Counts vs. Mass-to-Charge (m/z)
344.9785
241.1441
280.9825
165.0409
197.1550
141.0169

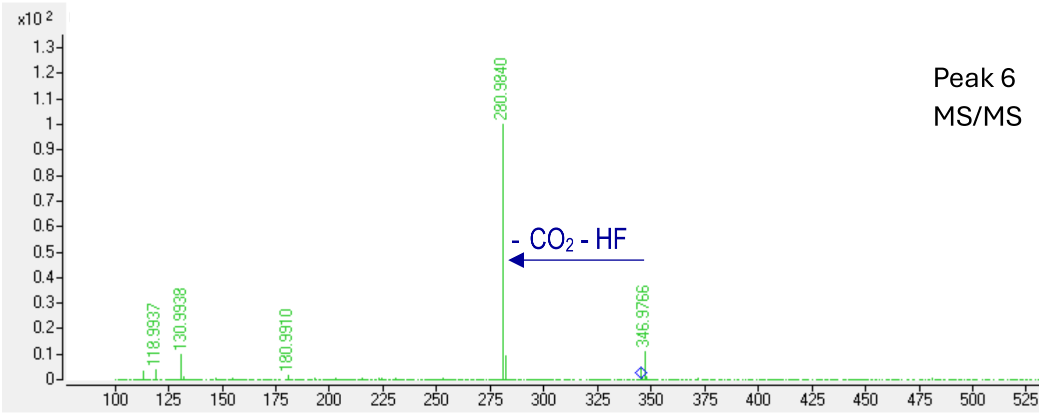
Peak 6
MS/MS
x10 2
280.9840
- CO2 - HF
346.9766
118.9937
130.9938
180.9910

Figure S16. EIC, MS and MS/MS spectra of m/z 294.9822

$C_5F_{10}H\text{-}COO^-$

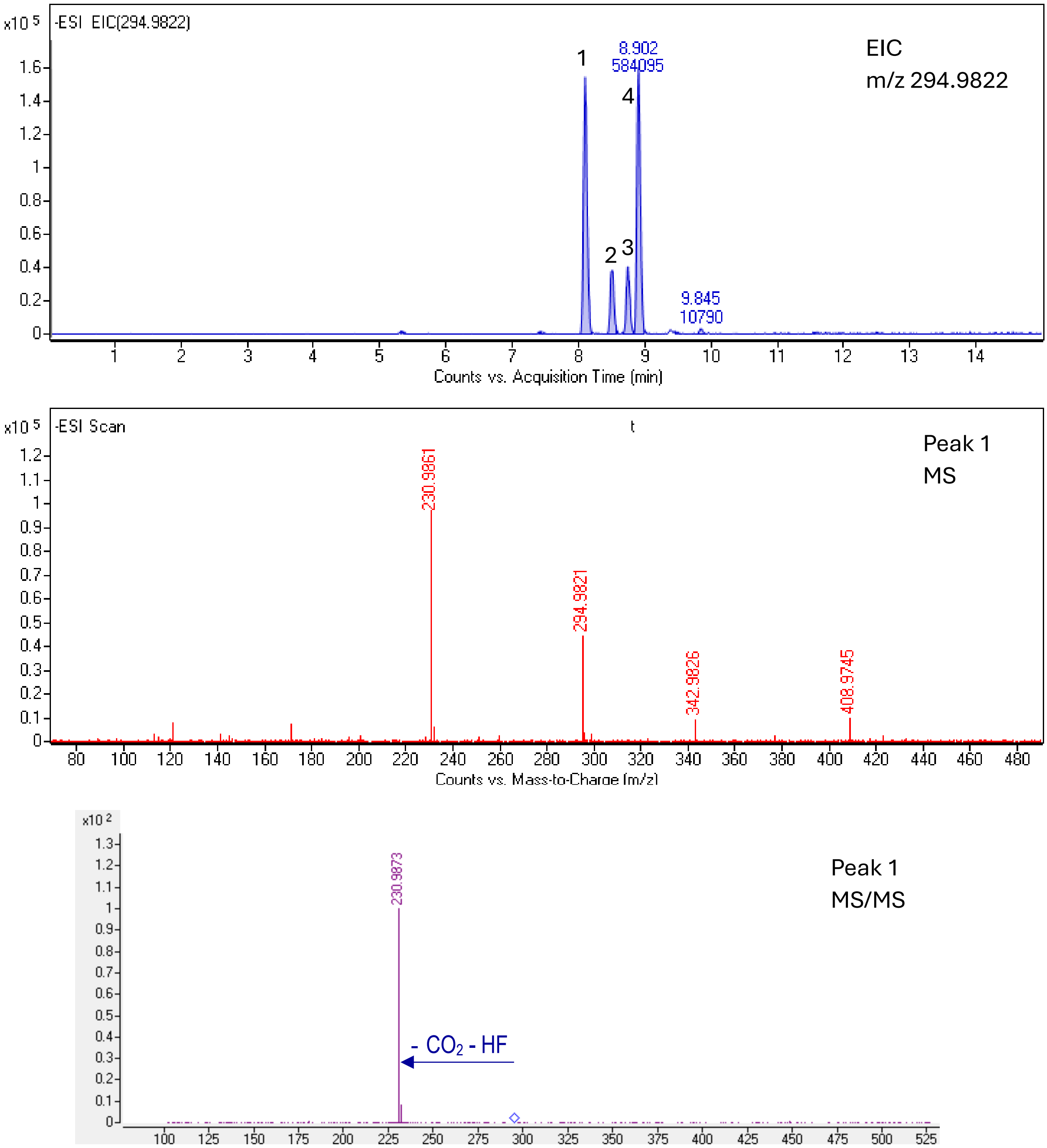

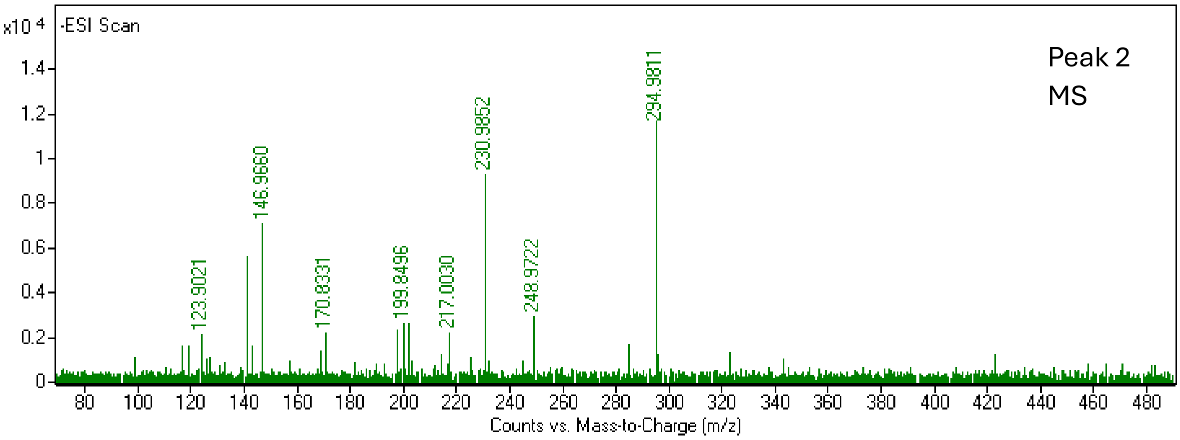

Peak 2
MS
-ESI Scan
x10 4
123.9021
146.9660
170.8331
199.8496
217.0030
230.9852
248.9722
294.9811
Counts vs. Mass-to-Charge (m/z)

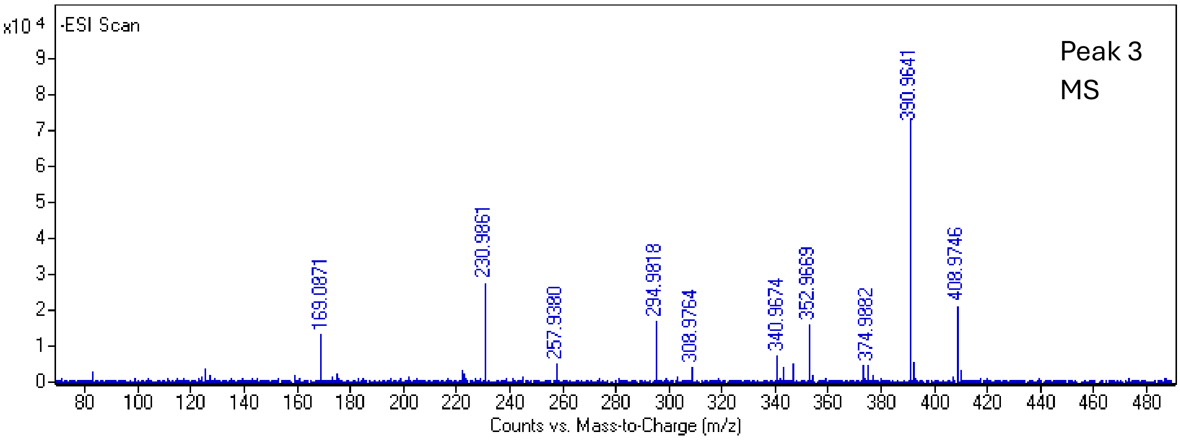

Peak 3
MS
-ESI Scan
x10 4
169.0871
230.9861
257.9380
294.9818
308.9764
340.9674
352.9669
374.9882
390.9641
408.9746
Counts vs. Mass-to-Charge (m/z)

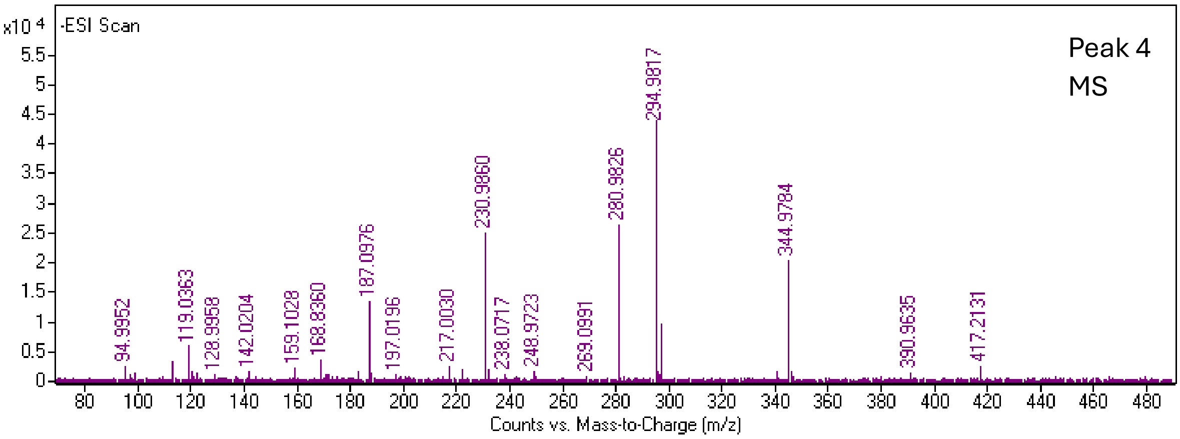

Peak 4
MS
-ESI Scan
x10 4
94.9952
119.0363
128.9958
142.0204
159.1028
168.8360
187.0976
197.0196
217.0030
230.9860
238.0717
248.9723
269.0991
280.9826
294.9817
344.9784
390.9635
417.2131
Counts vs. Mass-to-Charge (m/z)

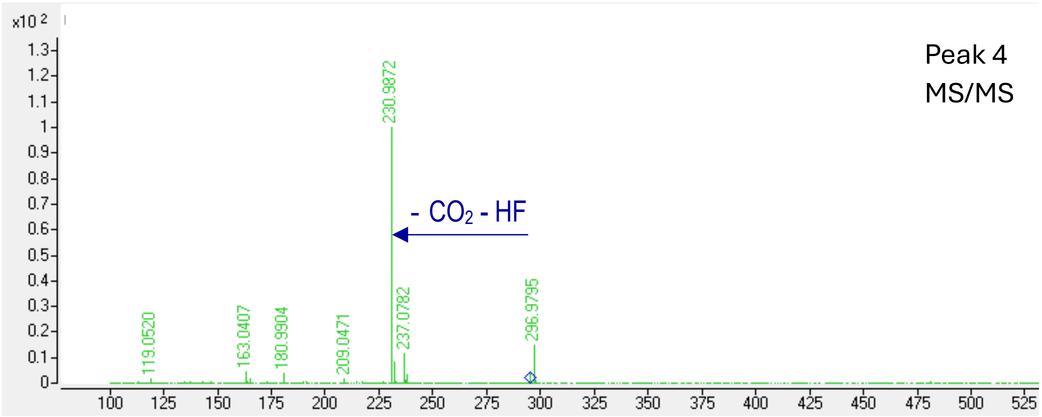

Peak 4
MS/MS
x10 2
- CO2 - HF
119.0520
163.0407
180.9904
209.0471
230.9872
237.0782
296.9795

Figure S17. EIC, MS and MS/MS spectra of 244.9854

$C_4F_8H\text{-}COO^-$

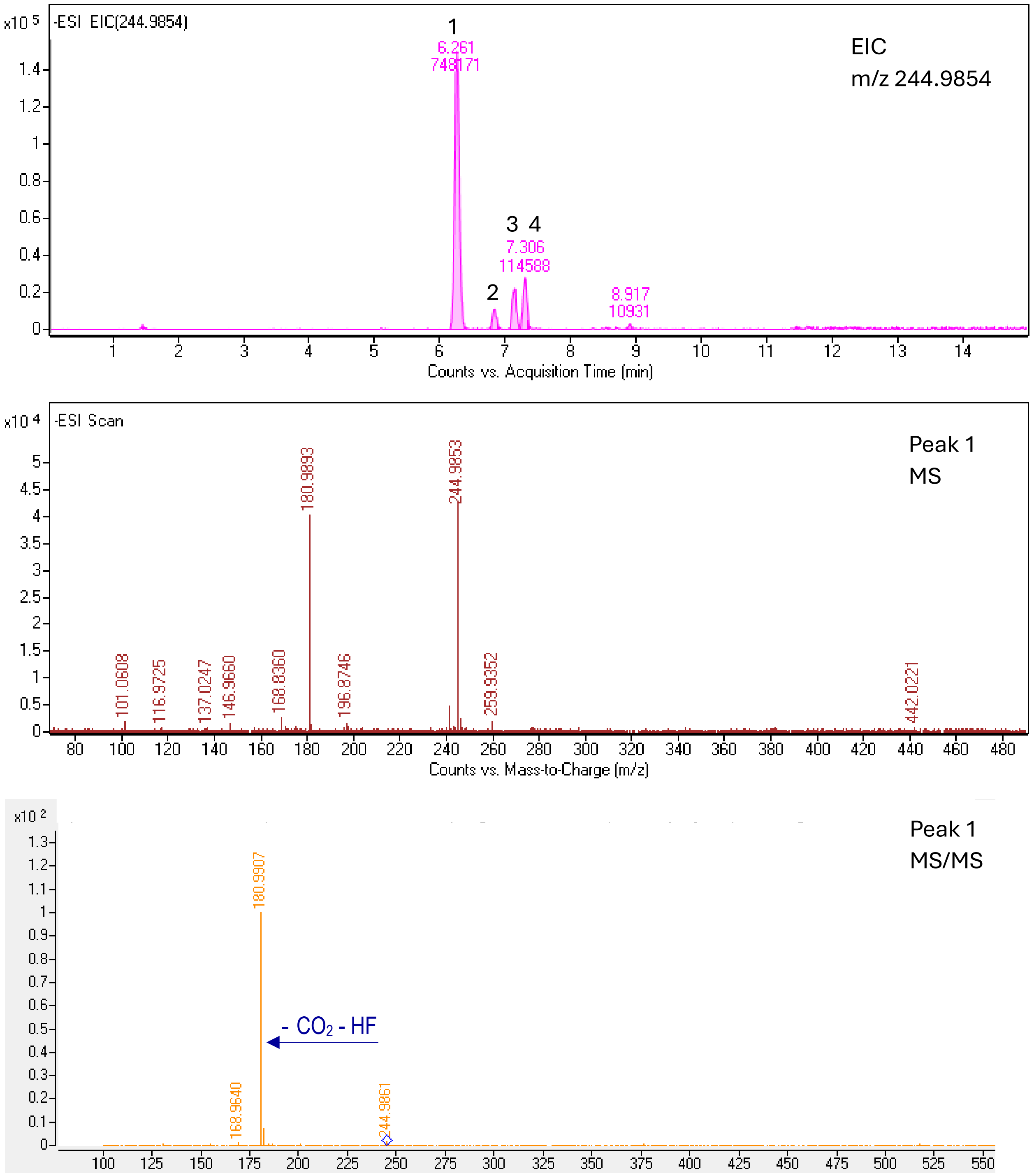

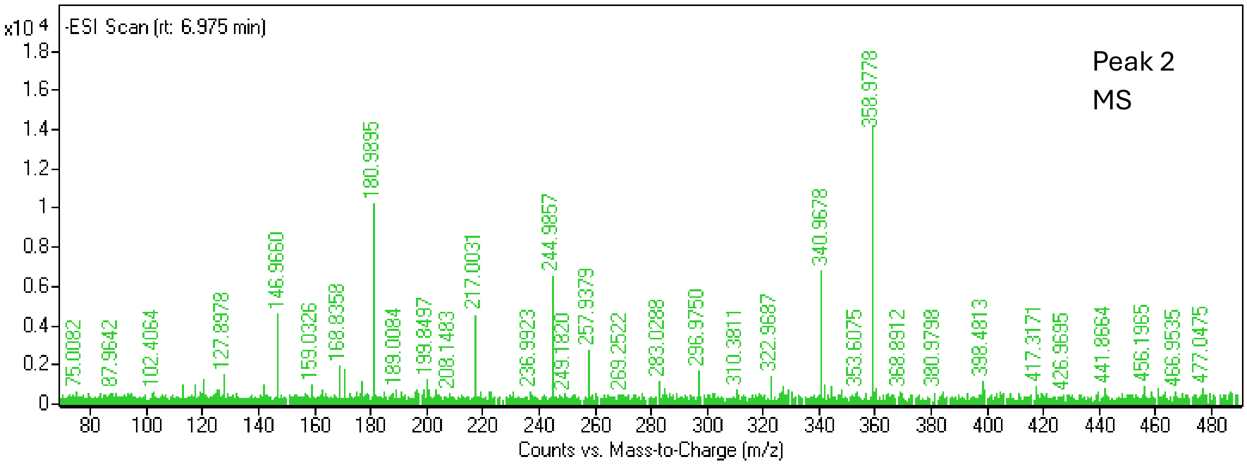
x10 4
-ESI Scan (rt: 6.975 min)
Peak 2
MS
75.0082
87.9642
102.4064
127.8978
146.9660
159.0326
168.8358
180.9895
189.0084
199.8497
208.1483
217.0031
236.9923
244.9857
249.1820
257.9379
269.2522
283.0288
296.9750
310.3811
322.9687
340.9678
353.6075
358.9778
368.8912
380.9798
398.4813
417.3171
426.9695
441.8664
456.1965
466.9535
477.0475
Counts vs. Mass-to-Charge (m/z)

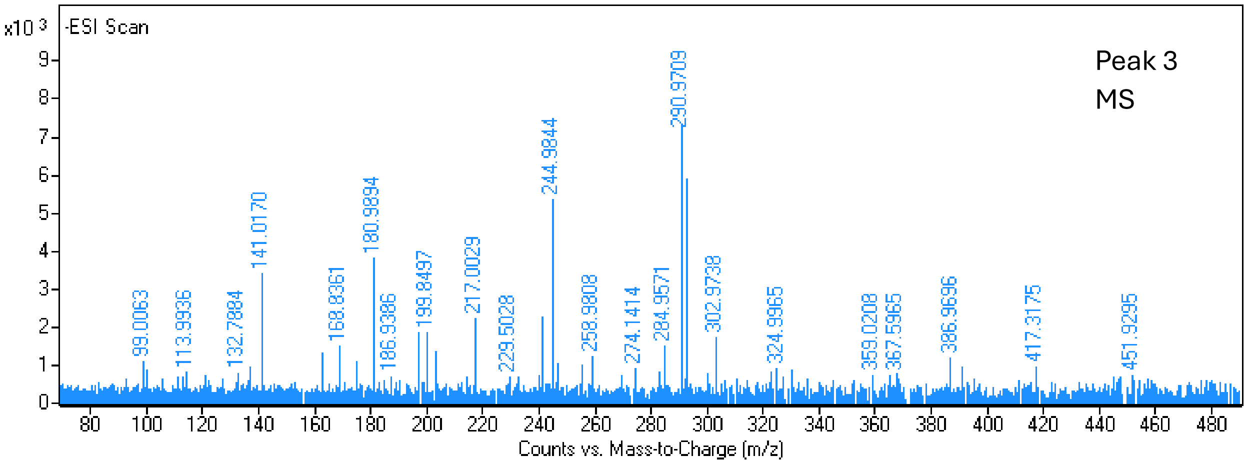
x10 3
-ESI Scan
Peak 3
MS
99.0063
113.9936
132.7884
141.0170
168.8361
180.9894
186.9386
199.8497
217.0029
229.5028
244.9844
258.9808
274.1414
284.9571
290.9709
302.9738
324.9965
359.0208
367.5965
386.9696
417.3175
451.9295
Counts vs. Mass-to-Charge (m/z)

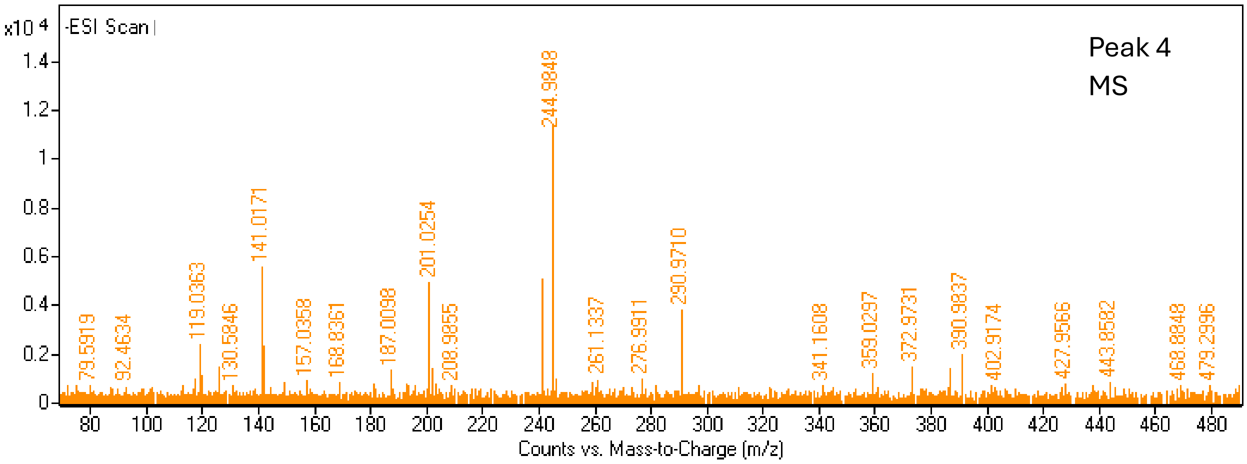
x10 4
-ESI Scan
Peak 4
MS
79.5919
92.4634
119.0363
130.5846
141.0171
157.0358
168.8361
187.0098
201.0254
208.9855
244.9848
261.1337
276.9911
290.9710
341.1608
359.0297
372.9731
390.9837
402.9174
427.9566
443.8582
468.8848
479.2996
Counts vs. Mass-to-Charge (m/z)

Figure S18. EIC, MS and MS/MS spectra of 494.9389

$C_8F_{15}H(OH)\text{-}SO_3^-$

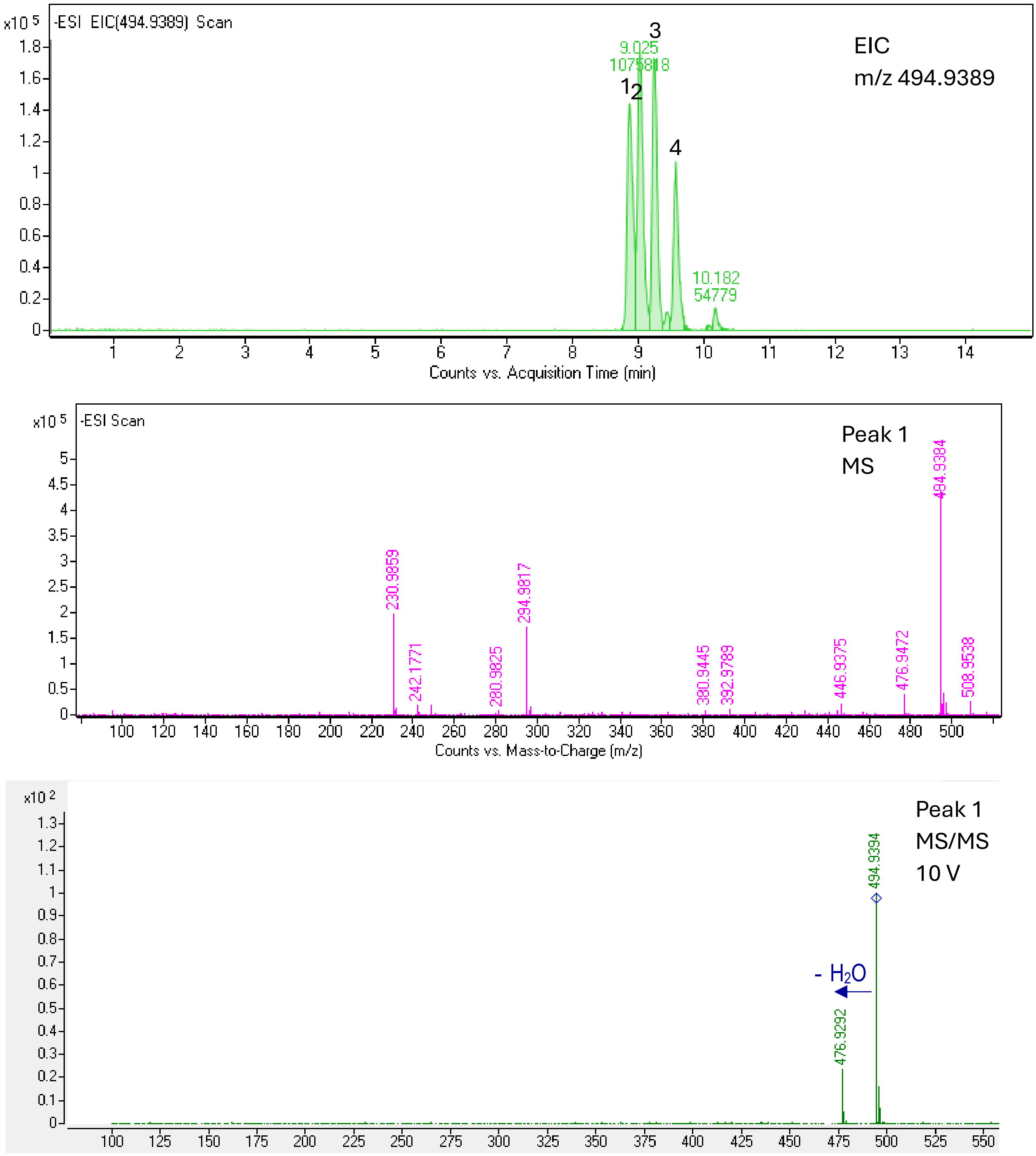

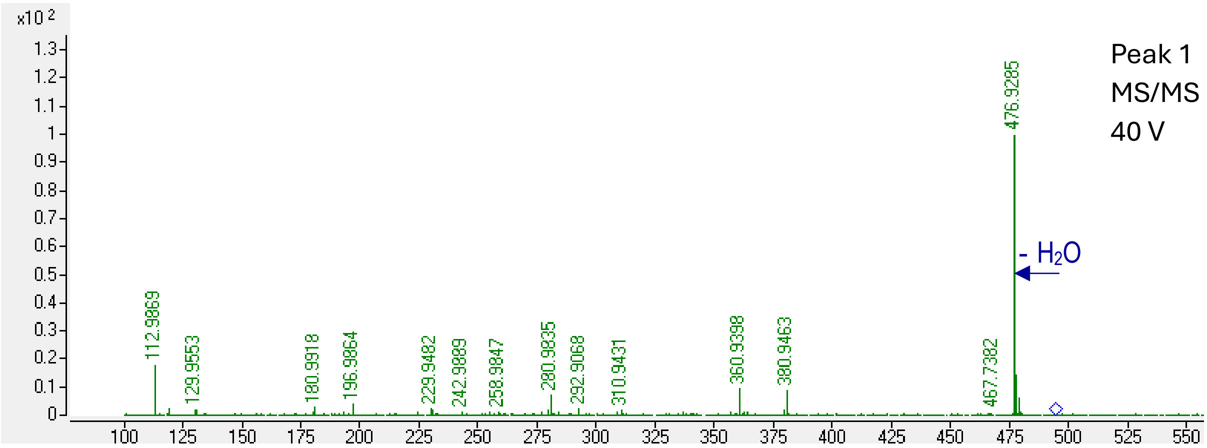
x10 2
Peak 1
MS/MS
40 V
- H_2O
112.9869
129.9553
180.9918
196.9864
229.9482
242.9889
258.9847
280.9835
292.9068
310.9431
360.9398
380.9463
467.7382
476.9285

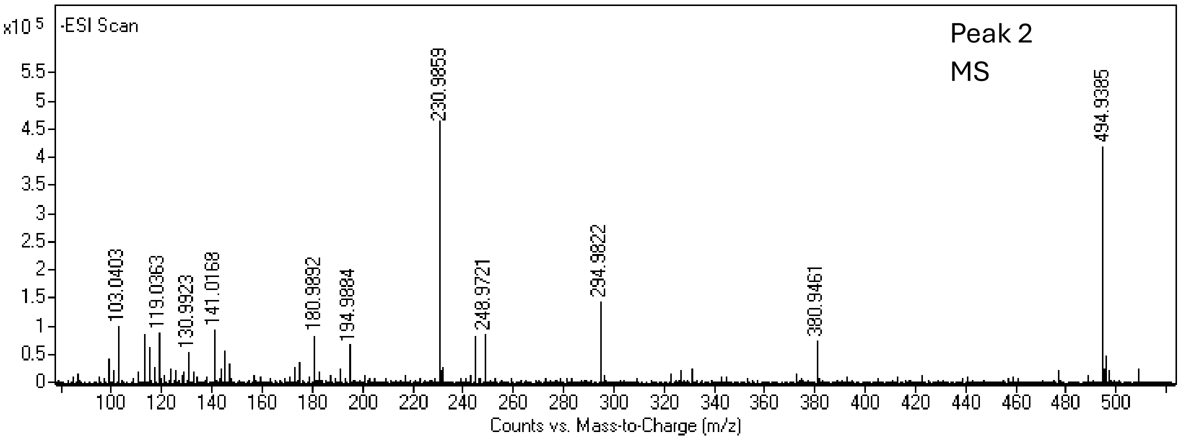
x10 5
-ESI Scan
Peak 2
MS
103.0403
119.0363
130.9923
141.0168
180.9892
194.9884
230.9859
248.9721
294.9822
380.9461
494.9385
Counts vs. Mass-to-Charge (m/z)

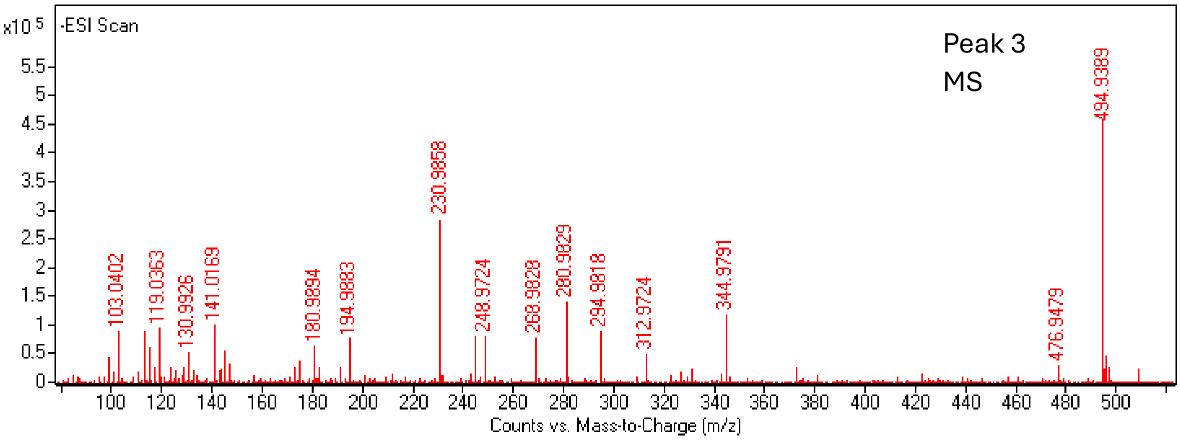
x10 5
-ESI Scan
Peak 3
MS
103.0402
119.0363
130.9926
141.0169
180.9894
194.9883
230.9858
248.9724
268.9828
280.9829
294.9818
312.9724
344.9791
476.9479
494.9389
Counts vs. Mass-to-Charge (m/z)

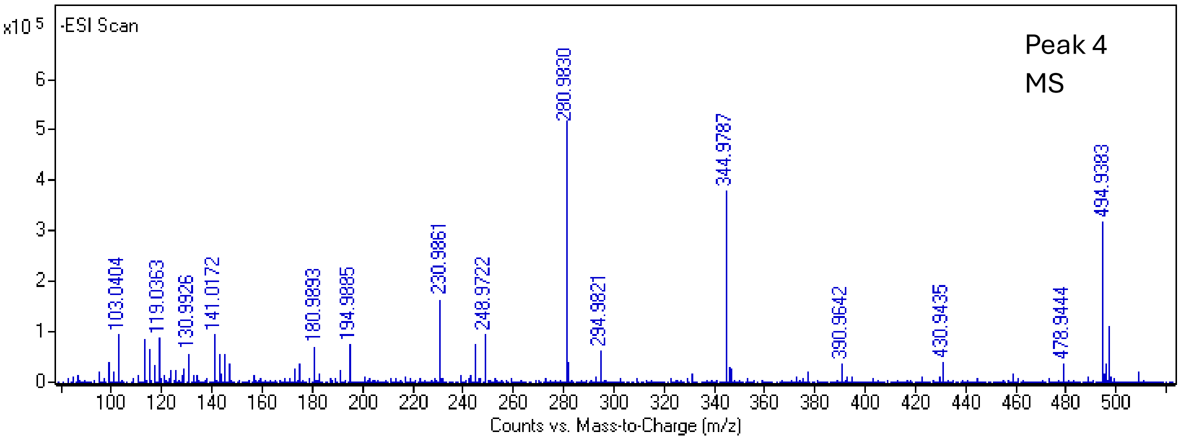
x10 5
-ESI Scan
Peak 4
MS
103.0404
119.0363
130.9926
141.0172
180.9893
194.9885
230.9861
248.9722
280.9830
294.9821
344.9787
390.9642
430.9435
478.9444
494.9383
Counts vs. Mass-to-Charge (m/z)

Figure S19. EIC, MS and MS/MS spectra of 496.9346

$C_8F_{16}(OH)\text{-}SO_3^-$

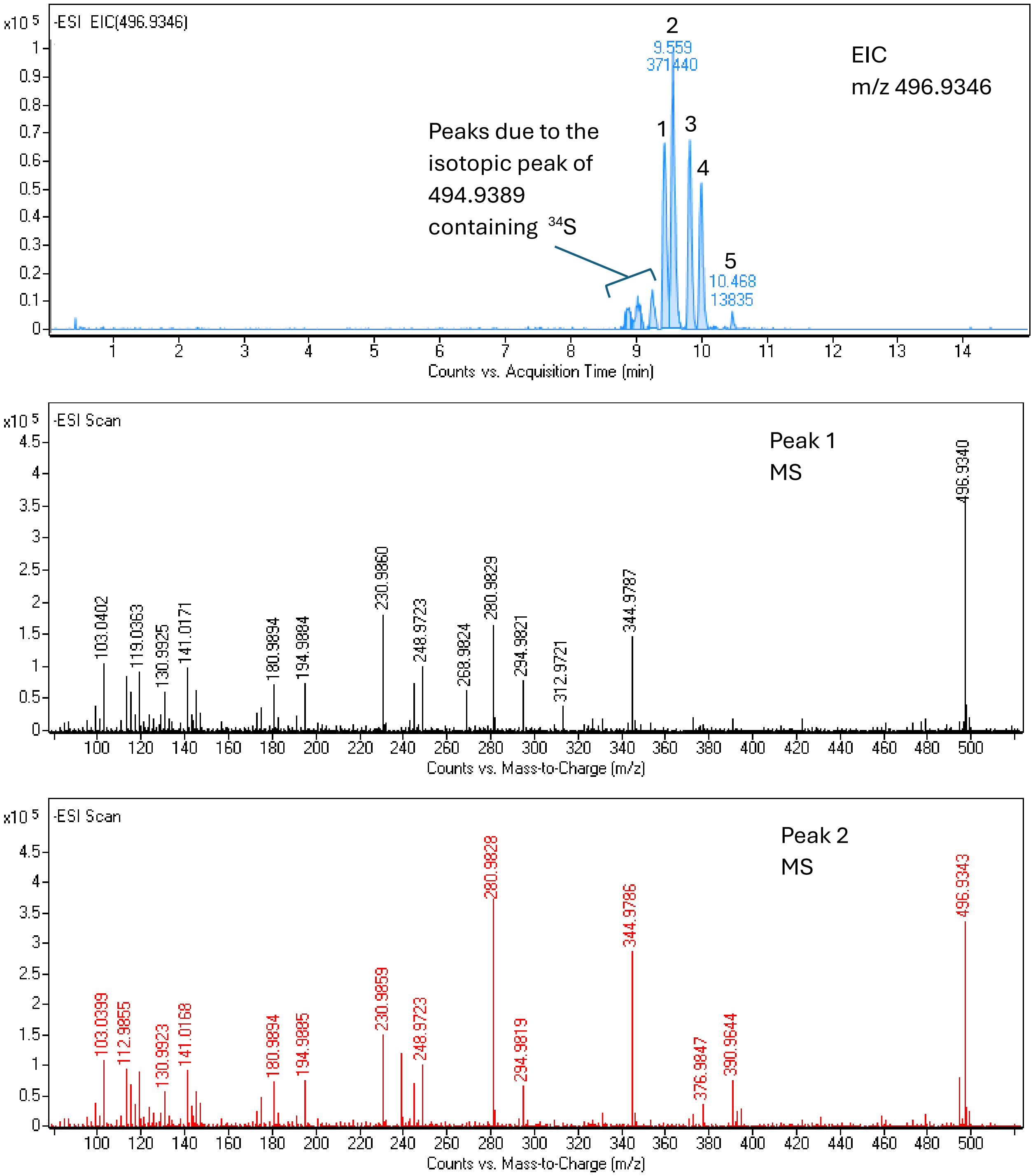

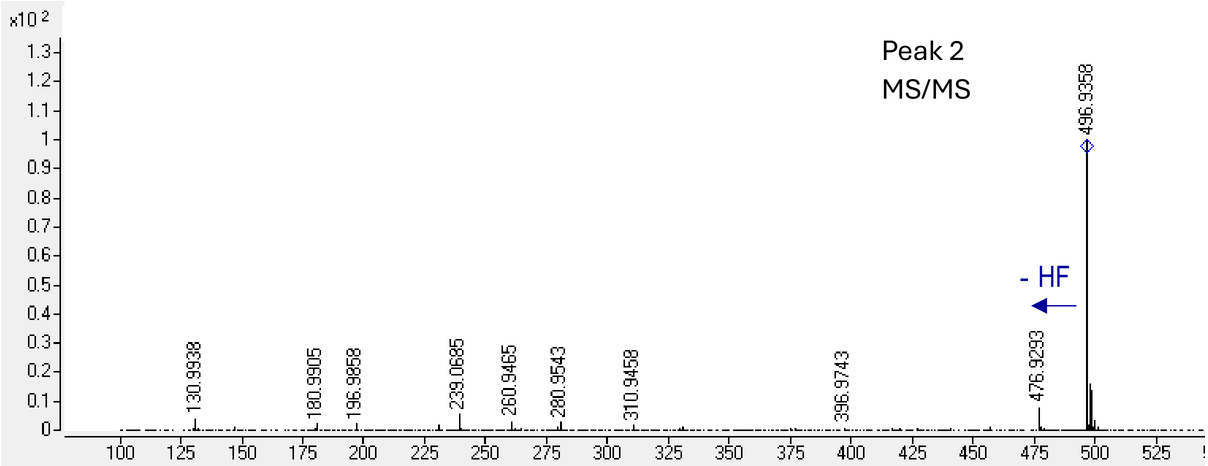
x10 2
Peak 2
MS/MS
- HF
496.9358
476.9293
396.9743
310.9458
280.9543
260.9465
239.0685
196.9858
180.9905
130.9938

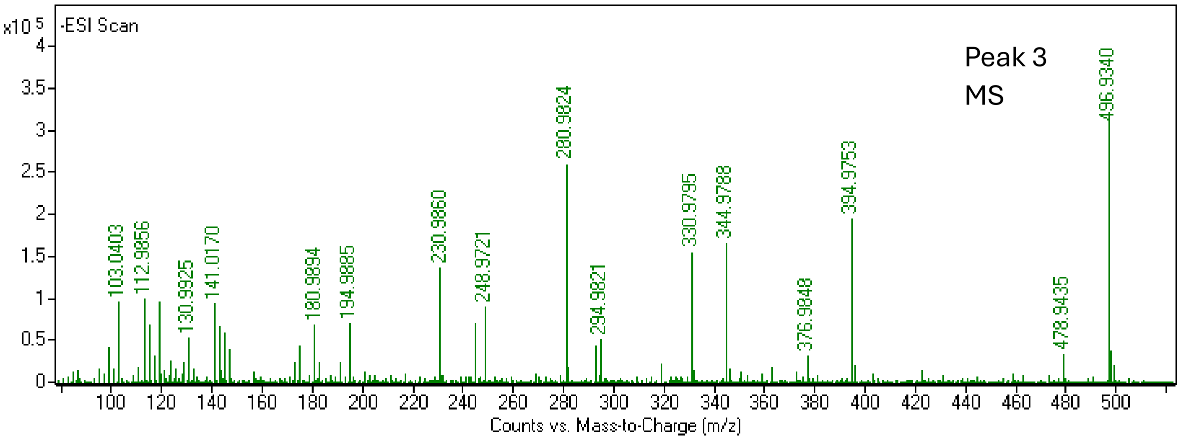
x10 5
-ESI Scan
Peak 3
MS
496.9340
280.9824
394.9753
230.9860
330.9795
344.9788
103.0403
112.9856
130.9925
141.0170
180.9894
194.9885
248.9721
294.9821
376.9848
478.9435
Counts vs. Mass-to-Charge (m/z)

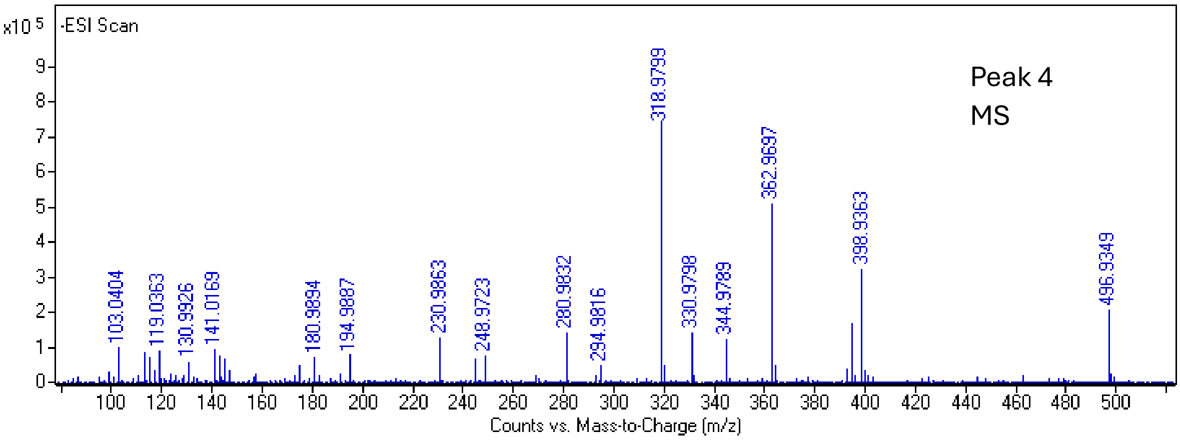
x10 5
-ESI Scan
Peak 4
MS
318.9799
362.9697
398.9363
496.9349
103.0404
119.0363
130.9926
141.0169
180.9894
194.9887
230.9863
248.9723
280.9832
294.9816
330.9798
344.9789
Counts vs. Mass-to-Charge (m/z)

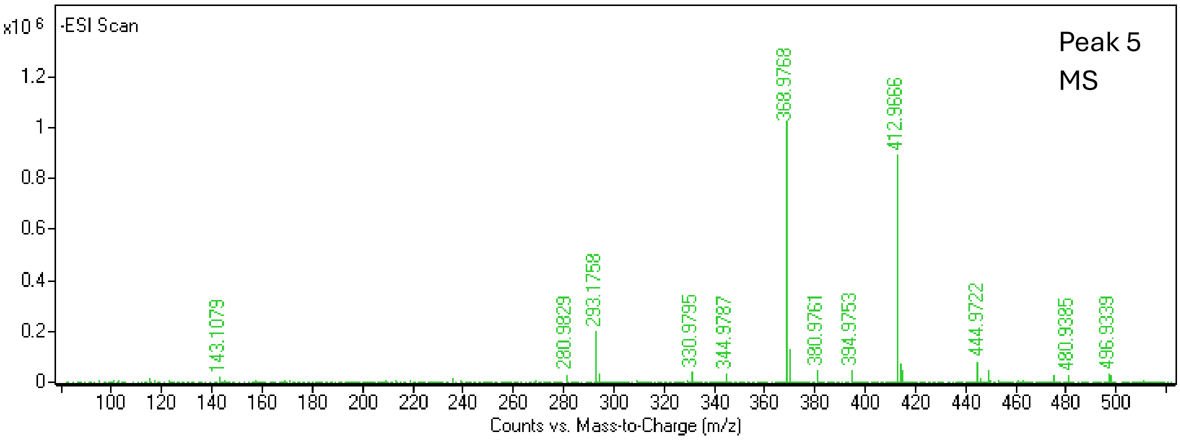
x10 6
-ESI Scan
Peak 5
MS
368.9768
412.9666
143.1079
280.9829
293.1758
330.9795
344.9787
380.9761
394.9753
444.9722
480.9385
496.9339
Counts vs. Mass-to-Charge (m/z)

Figure S20. EIC, MS and MS spectra of m/z 380.9460

$C_6F_{12}H$-SO_3^-

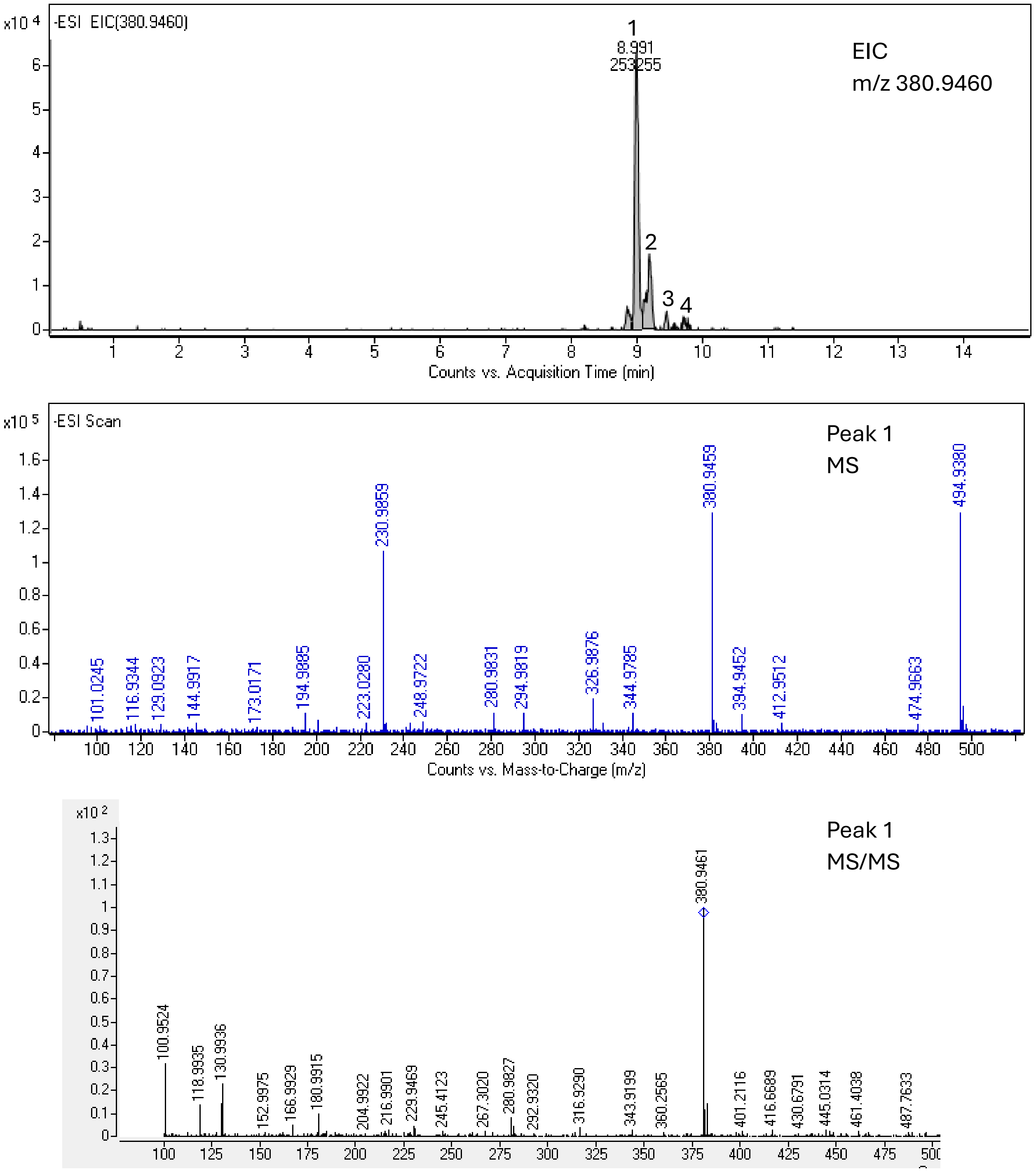

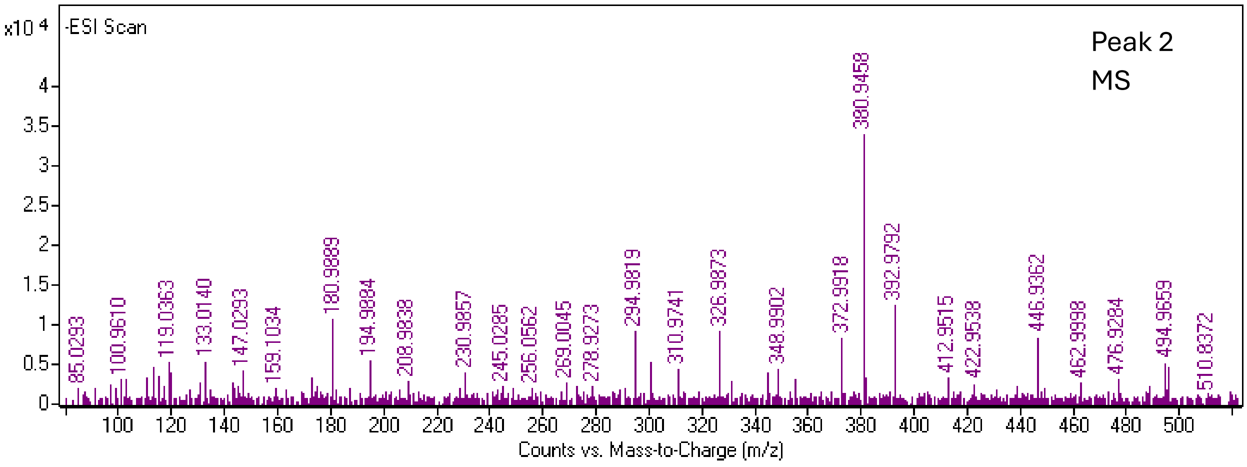
x10 4
-ESI Scan
Peak 2
MS
85.0293
100.9610
119.0363
133.0140
147.0293
159.1034
180.9889
194.9884
208.9838
230.9857
245.0285
256.0562
269.0045
278.9273
294.9819
310.9741
326.9873
348.9902
372.9918
380.9458
392.9792
412.9515
422.9538
446.9362
462.9998
476.9284
494.9659
510.8372
Counts vs. Mass-to-Charge (m/z)

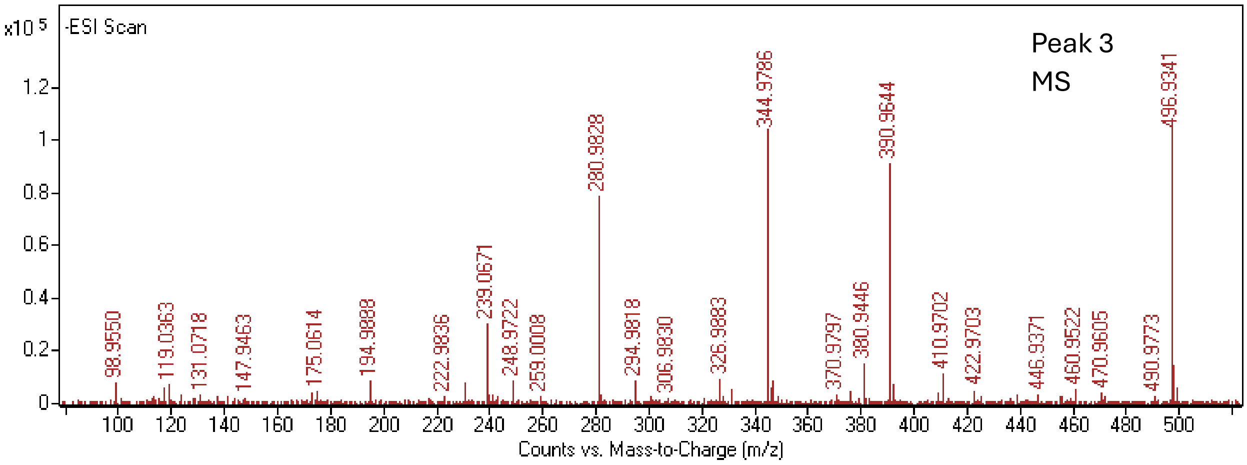
x10 5
-ESI Scan
Peak 3
MS
98.9550
119.0363
131.0718
147.9463
175.0614
194.9888
222.9836
239.0671
248.9722
259.0008
280.9828
294.9818
306.9830
326.9883
344.9786
370.9797
380.9446
390.9644
410.9702
422.9703
446.9371
460.9522
470.9605
490.9773
496.9341
Counts vs. Mass-to-Charge (m/z)

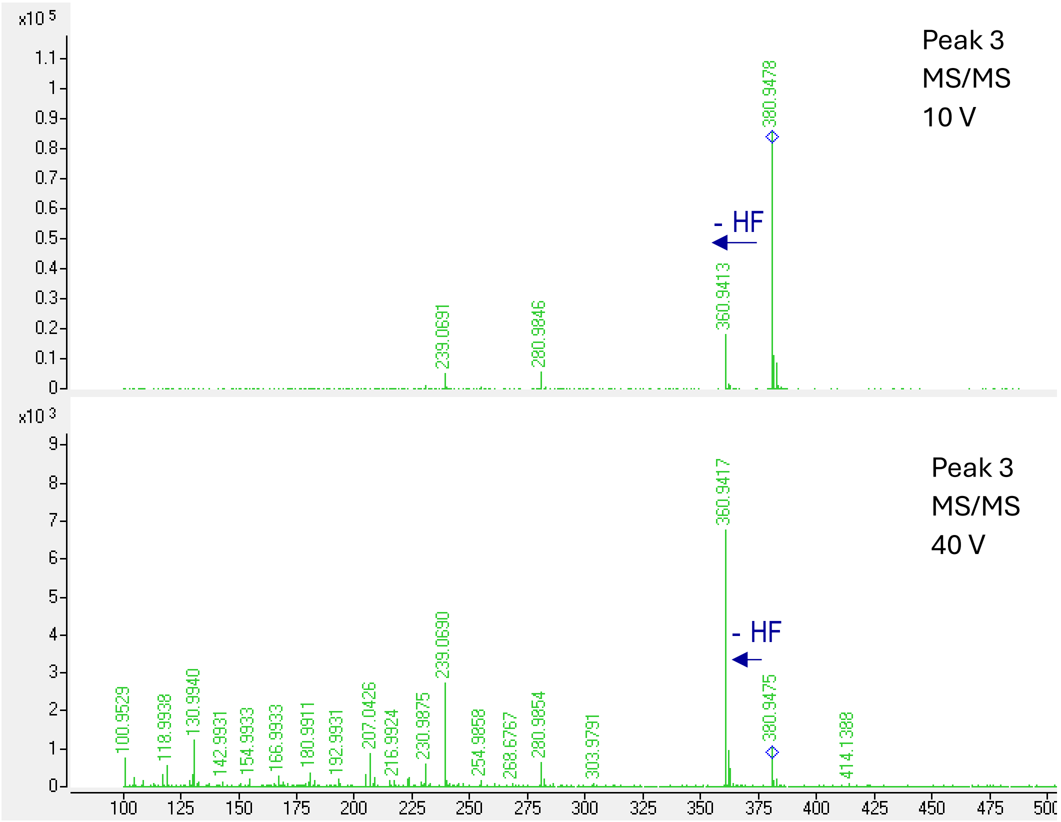
x10 5
Peak 3
MS/MS
10 V
239.0691
280.9846
360.9413
380.9478
- HF
x10 3
Peak 3
MS/MS
40 V
100.9529
118.9938
130.9940
142.9931
154.9933
166.9933
180.9911
192.9931
207.0426
216.9924
230.9875
239.0690
254.9858
268.6767
280.9854
303.9791
360.9417
380.9475
414.1388
- HF

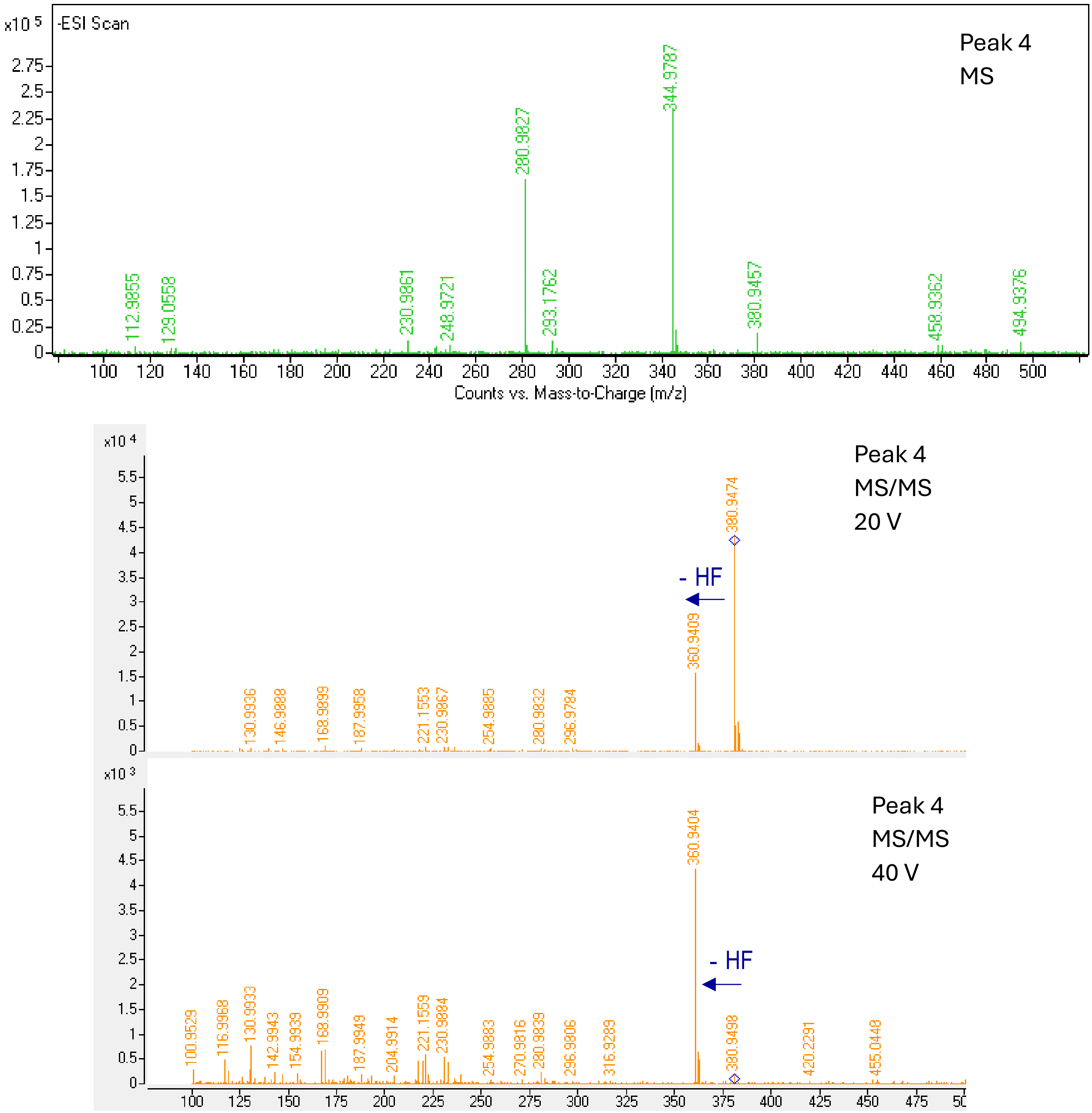
-ESI Scan
x10 5
Peak 4
MS
112.9855
129.0558
230.9861
248.9721
280.9827
293.1762
344.9787
380.9457
458.9362
494.9376
Counts vs. Mass-to-Charge (m/z)
x10 4
Peak 4
MS/MS
20 V
380.9474
- HF
360.9409
130.9936
146.9888
168.9899
187.9958
221.1553
230.9867
254.9885
280.9832
296.9784
x10 3
Peak 4
MS/MS
40 V
360.9404
- HF
100.9529
116.9968
130.9933
142.9943
154.9939
168.9909
187.9949
204.9914
221.1559
230.9884
254.9883
270.9816
280.9839
296.9806
316.9289
380.9498
420.2291
455.0449

Figure S21. EIC, MS and MS spectra of m/z 280.9520

$C_4F_8H\text{-}SO_3^-$

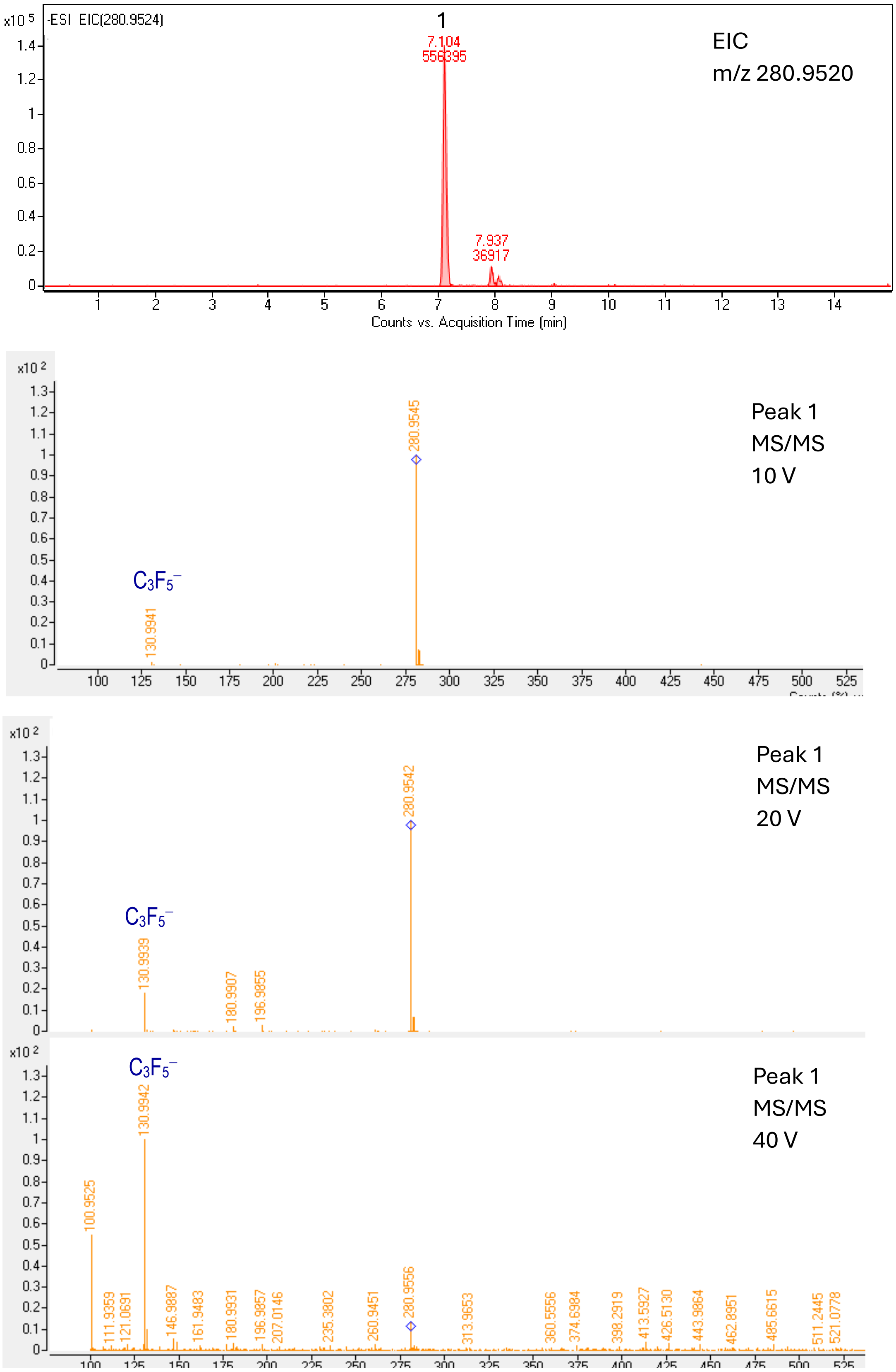

Figure S22. EIC, MS and MS spectra of m/z 230.9556

$C_3F_6H\text{-}SO_3^-$

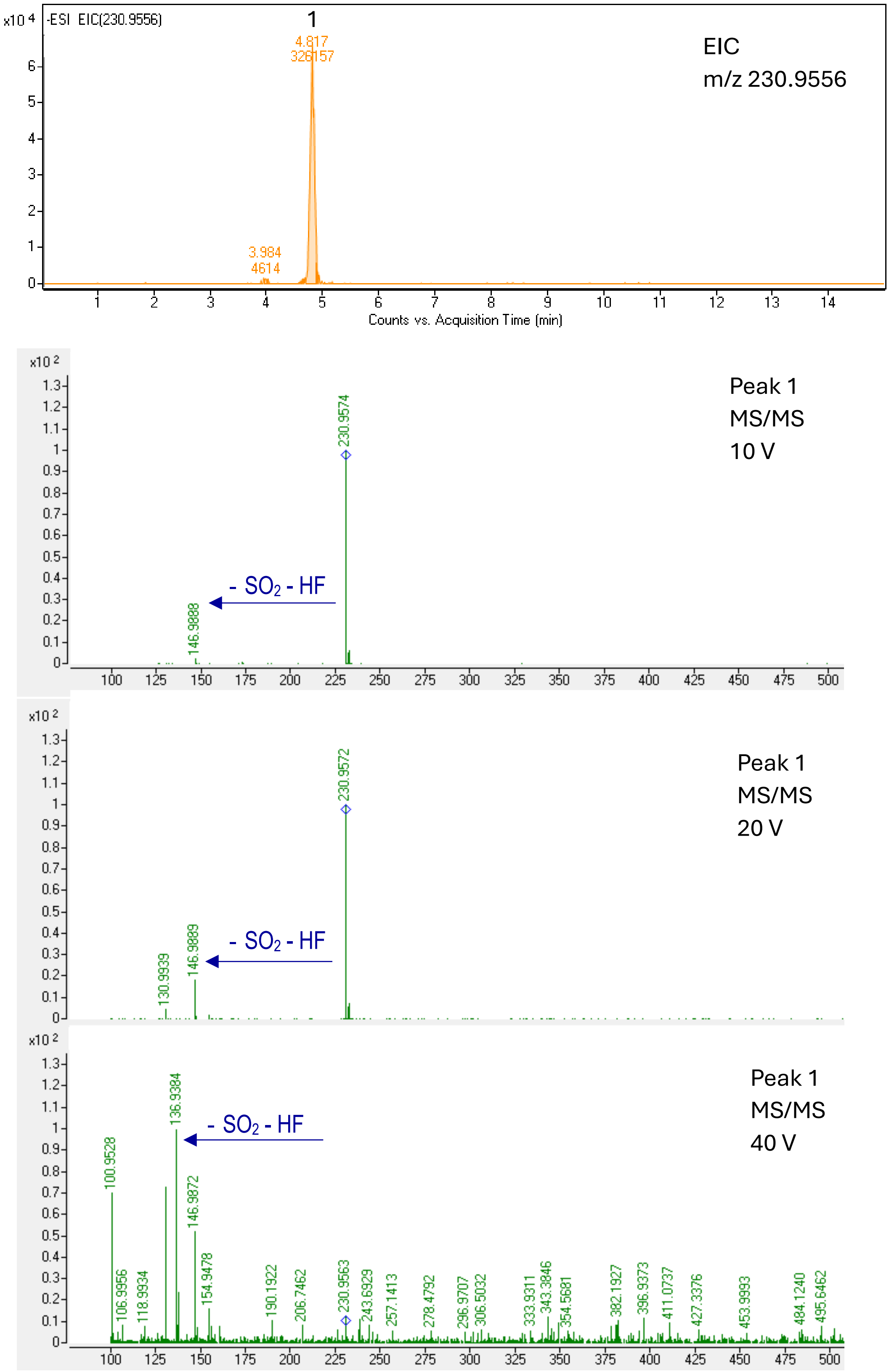

Figures S23-S25. Hypothetical structures and fragmentation mechanisms of some substitution products.

Some hypothetical structures and fragmentation mechanisms of the substitution products are shown below.

Figure S23. Hypothesis of structure and fragmentation mechanism for a $C_7F_{13}(OH)_2$-COO^- isomer, exemplary case for $C_nF_{2n+1}(OH)_2$-COO^- (n = 5-7) substitution products.

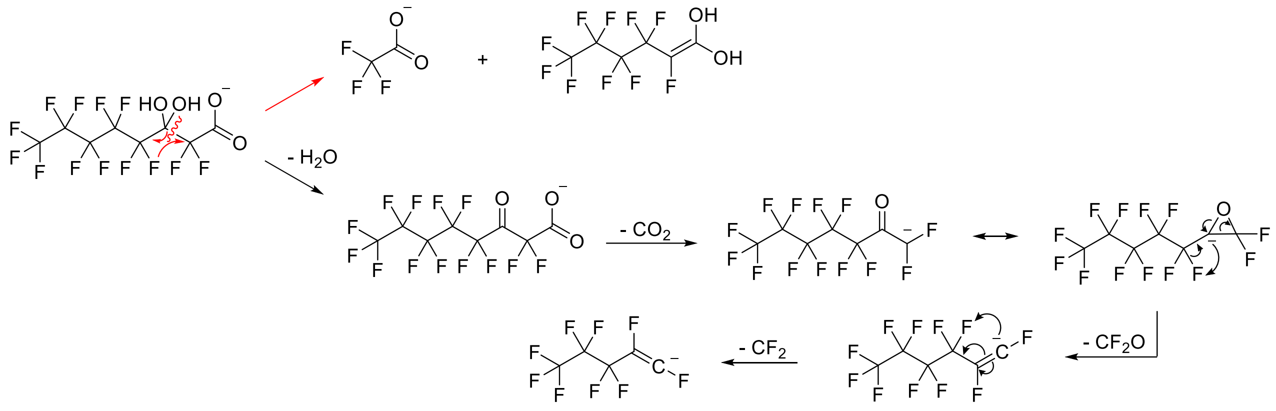

Figure S24. Hypothesis of structure and fragmentation mechanism for a $C_7F_{13}H(OH)$-COO^- isomer, exemplary case for some $C_nF_{2n+1}H(OH)$-COO^- (n = 4-7) substitution products.

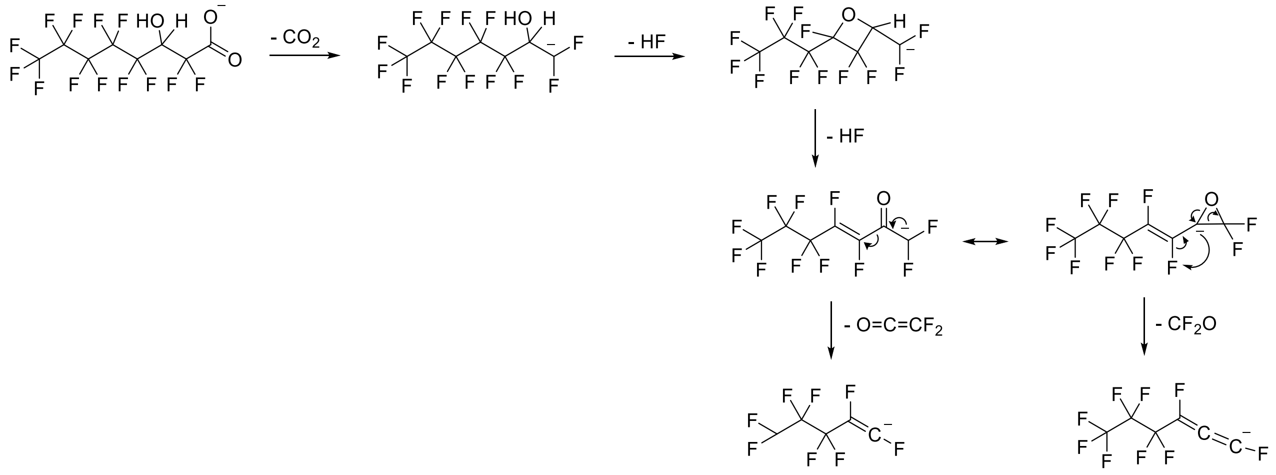

Figure S25. Hypothesis of structure and fragmentation mechanism for a $C_7F_{14}H$-COO^- isomer, exemplary case for $C_nF_{2n}H$-COO^- (n = 4-7) substitution products.

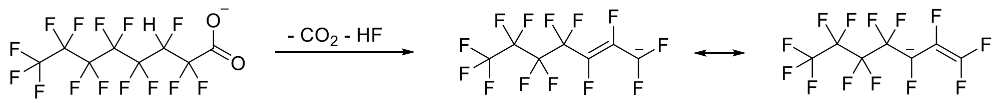

Figures S26-S27. Examples of EIC before and after SPE using the first two tested procedures described in the text.

Figure S26. (a) EIC of a hydroxy-defluorination product ($C_7F_{13}(OH)_2COO^-$, m/z 409) in the original sample and (b) after SPE using the first procedure. (c) Comparison between EIC of PFBA (C4) before and (d) after the SPE.

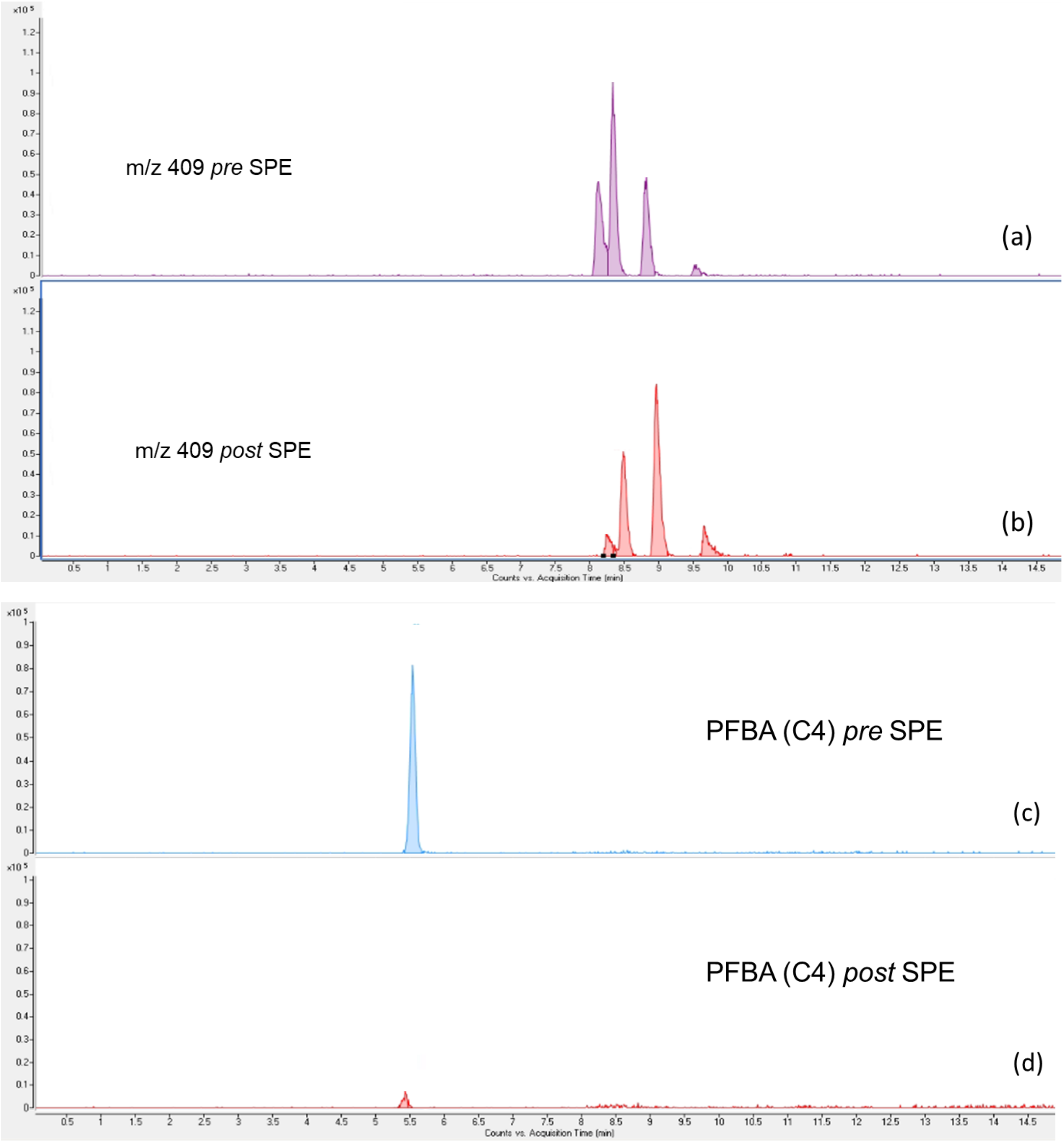

Figure S27. a) EIC of a polyfluorodihydroxy carboxylic acid ($C_7F_{13}(OH)_2COO^-$, m/z 409) in the original sample and b) after SPE using the second procedure. c) Comparison between EIC of PFBA (C4) before and (d) after the SPE.

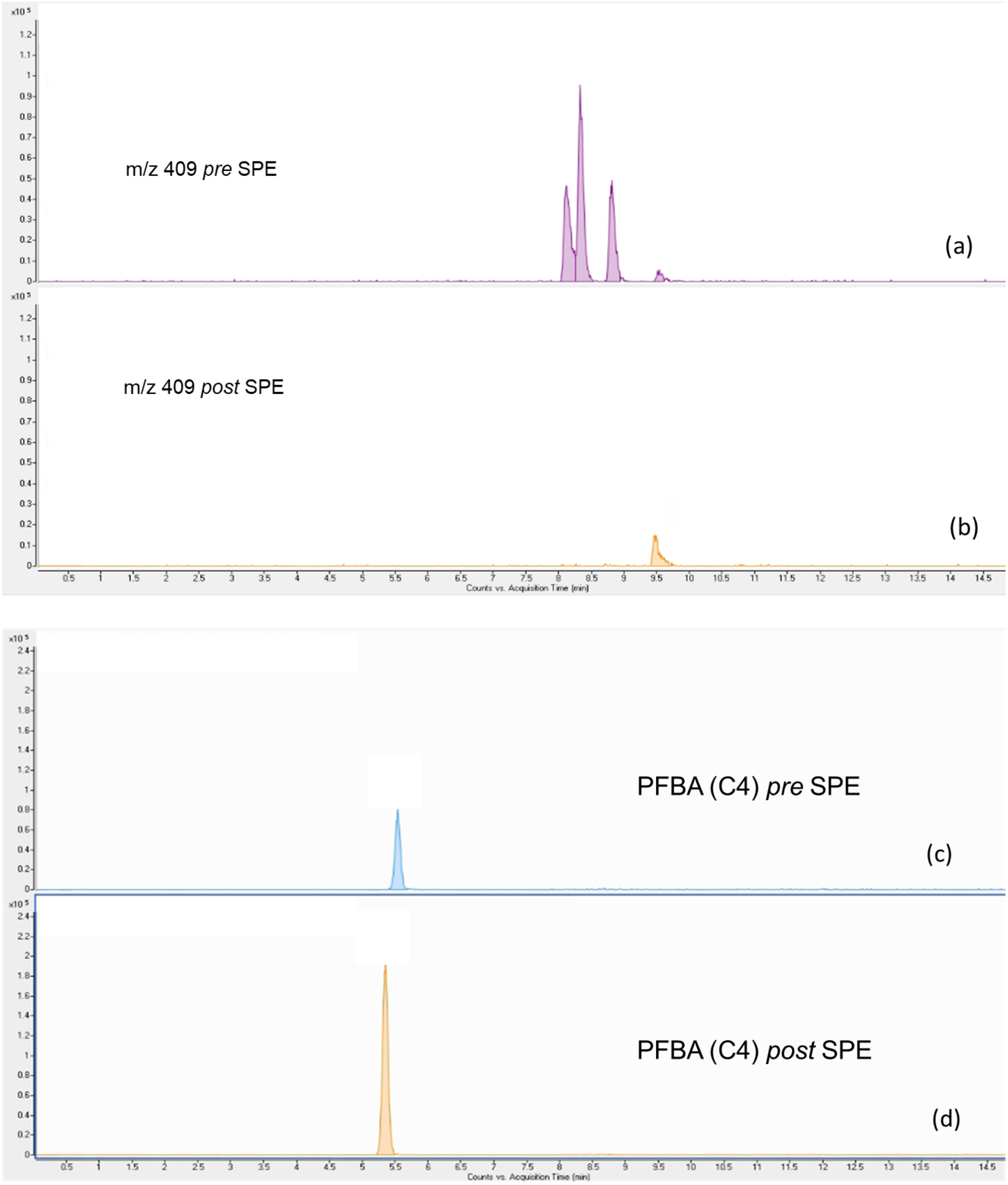

Figures S28-S30. Concentration of PFCAs and substitution products in the untreated groundwater (0 min) and after 15, 30 and 60 minutes of non-thermal plasma treatment.

Figure S28. Concentration of perfluorinated carboxylic acids detected in the untreated groundwater (0 min) and after 15, 30 and 60 minutes of non-thermal plasma treatment.

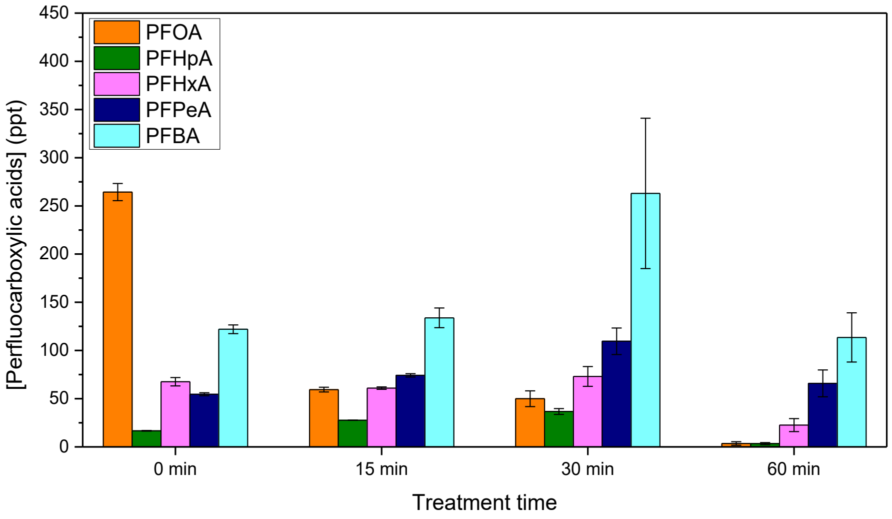

Figure S29. Concentration of hydro-defluorinated substitution products distinguished based on the number of carbon atoms (Cx-C are carboxylic acids, Cx-S are sulfonic detected in the untreated groundwater (0 min) and after 15, 30 and 60 minutes of non-thermal plasma treatment.

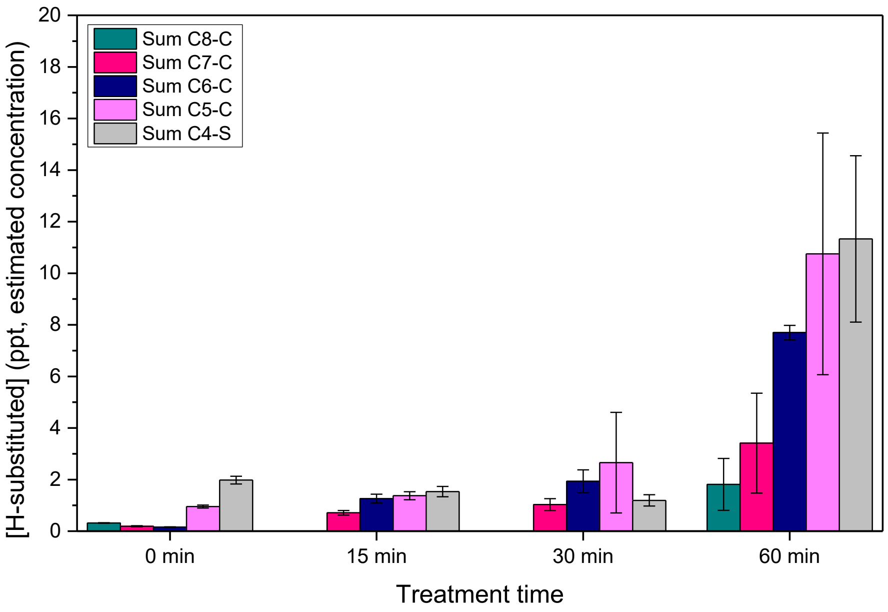

Figure S30. Concentration of hydroxy-defluorined substitution products distinguished based on the number of carbon atoms detected in the untreated groundwater (0 min) and after 15, 30 and 60 minutes of non-thermal plasma treatment.

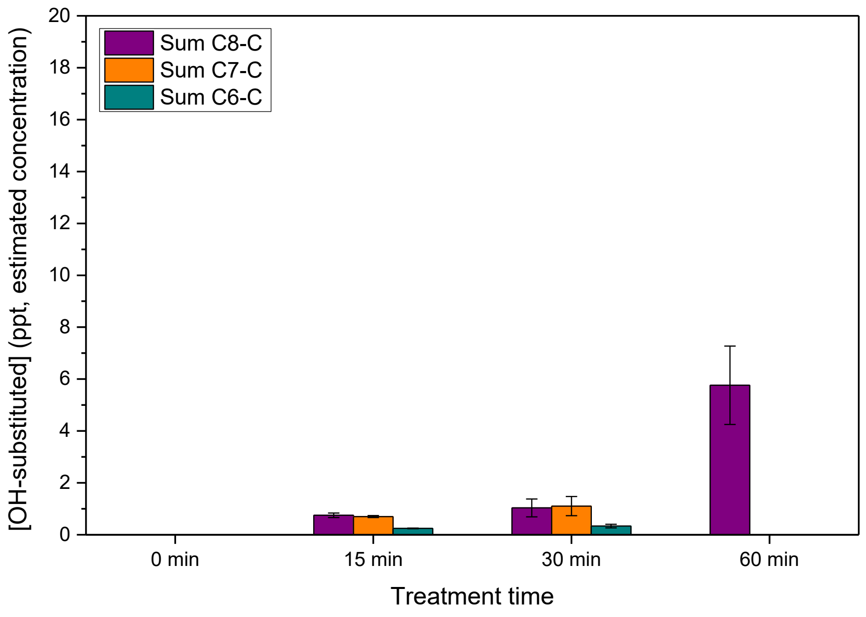

Supplement: Supplementary file 1 [file es5c01886_si_001.pdf]
